# Long Noncoding RNA-Enriched Vesicles Secreted by Hypoxic Cardiomyocytes Drive Cardiac Fibrosis

**DOI:** 10.1016/j.omtn.2019.09.003

**Published:** 2019-09-13

**Authors:** Franziska Kenneweg, Claudia Bang, Ke Xiao, Chantal M. Boulanger, Xavier Loyer, Stephane Mazlan, Blanche Schroen, Steffie Hermans-Beijnsberger, Ariana Foinquinos, Marc N. Hirt, Thomas Eschenhagen, Sandra Funcke, Stevan Stojanovic, Celina Genschel, Katharina Schimmel, Annette Just, Angelika Pfanne, Kristian Scherf, Susann Dehmel, Stella M. Raemon-Buettner, Jan Fiedler, Thomas Thum

**Affiliations:** 1 Institute of Molecular and Translational Therapeutic Strategies (IMTTS), Hannover Medical School, Hannover, Germany; 2 INSERM UMR-970, Paris Cardiovascular Research Center, Université Paris Descartes Hȏpital Européen Georges, Paris, France; 3 Department of Cardiology, CARIM School for Cardiovascular Diseases, Maastricht University, Maastricht, the Netherlands; 4 Institute of Experimental Pharmacology and Toxicology, University Medical Center Hamburg-Eppendorf, Hamburg, Germany; 5 DZHK (German Centre for Cardiovascular Research), partner site Hamburg/Kiel/Lübeck, Germany; 6 Department of Anesthesiology, Center of Anesthesiology and Intensive Care Medicine, University Medical Center Hamburg-Eppendorf, Hamburg, Germany; 7 Fraunhofer Institute for Toxicology and Experimental Medicine (ITEM), Hannover, Hannover, Germany; 8 REBIRTH Excellence Cluster, Hannover Medical School, Hannover, Germany

**Keywords:** lncRNA, hypoxia, myocardial infarction, extracellular vesicles, *Neat1*

## Abstract

Long non-coding RNAs (lncRNAs) have potential as novel therapeutic targets in cardiovascular diseases, but detailed information about the intercellular lncRNA shuttling mechanisms in the heart is lacking. Here, we report an important novel crosstalk between cardiomyocytes and fibroblasts mediated by the transfer of lncRNA-enriched extracellular vesicles (EVs) in the context of cardiac ischemia. lncRNA profiling identified two hypoxia-sensitive lncRNAs: *ENSMUST00000122745* was predominantly found in small EVs, whereas lncRNA *Neat1* was enriched in large EVs *in vitro* and *in vivo*. Vesicles were taken up by fibroblasts, triggering expression of profibrotic genes. In addition, lncRNA *Neat1* was transcriptionally regulated by P53 under basal conditions and by HIF2A during hypoxia. The function of *Neat1* was further elucidated *in vitro* and *in vivo*. Silencing of *Neat1 in vitro* revealed that *Neat1* was indispensable for fibroblast and cardiomyocyte survival and affected fibroblast functions (reduced migration capacity, stalled cell cycle, and decreased expression of fibrotic genes). Of translational importance, genetic loss of *Neat1 in vivo* resulted in an impaired heart function after myocardial infarction highlighting its translational relevance.

## Graphical Abstract

**Figure:**
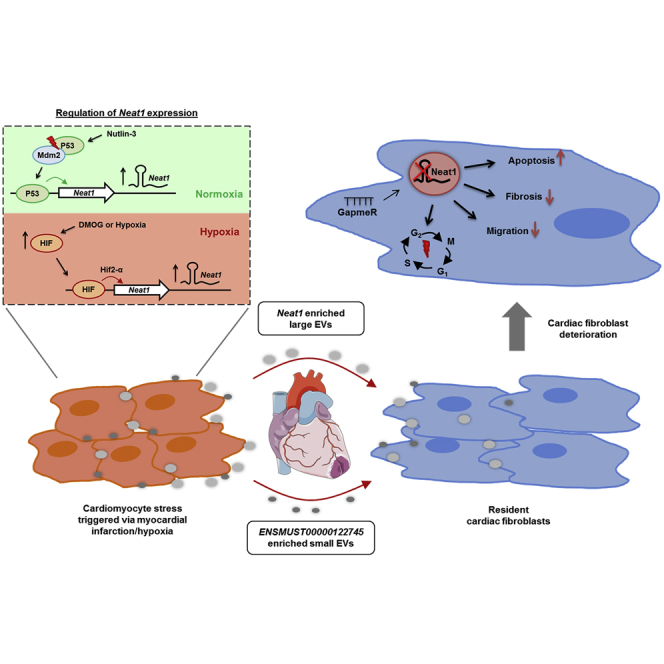

## Introduction

Cardiovascular diseases (CVDs), including myocardial infarction (MI), lead to an adverse remodeling process in the heart. This is typically initiated by different stress factors such as cardiac ischemia as well as pressure and/or volume overload, resulting in maladaptive responses to maintain the cardiac function and, ultimately, contributing to heart failure.^1^, ^2^, ^3^ There is intercellular crosstalk between main cell types of the heart such as cardiac fibroblasts, cardiomyocytes, and endothelial cells to coordinate the initiation and progression of cardiac remodeling.^4^, ^5^ Since the discovery that non-coding RNAs (ncRNAs) such as microRNAs and long non-coding RNAs (lncRNAs) are also present in human body fluids, partly via the inclusion into extracellular vesicles (EVs),^6^, ^7^ they have emerged as paracrine effectors by which cardiac cell types can communicate with each other and respond to stress conditions and are considered to serve as novel clinical biomarkers. In the context of ncRNAs/vesicle-mediated communication mechanisms in the heart, previous studies identified that cardiac fibroblasts are able to release exosomes enriched in microRNAs that are taken up by cardiomyocytes, contributing to the development of cardiomyocyte hypertrophy.^8^ However, whether lncRNA-enriched EVs may also serve as cell-cell communicators during cardiac ischemia is not well known so far. In this study, we characterized a novel intercellular communication route between hypoxic cardiomyocytes and fibroblasts via the transfer of lncRNA-enriched EVs and studied their potential biological function in ischemic heart disease including *in vitro*, *in vivo*, and human cell/tissue studies.

## Results

### Ischemia Drives Extracellular Vesicle Secretion by Cardiomyocytes

**Figure 1:**
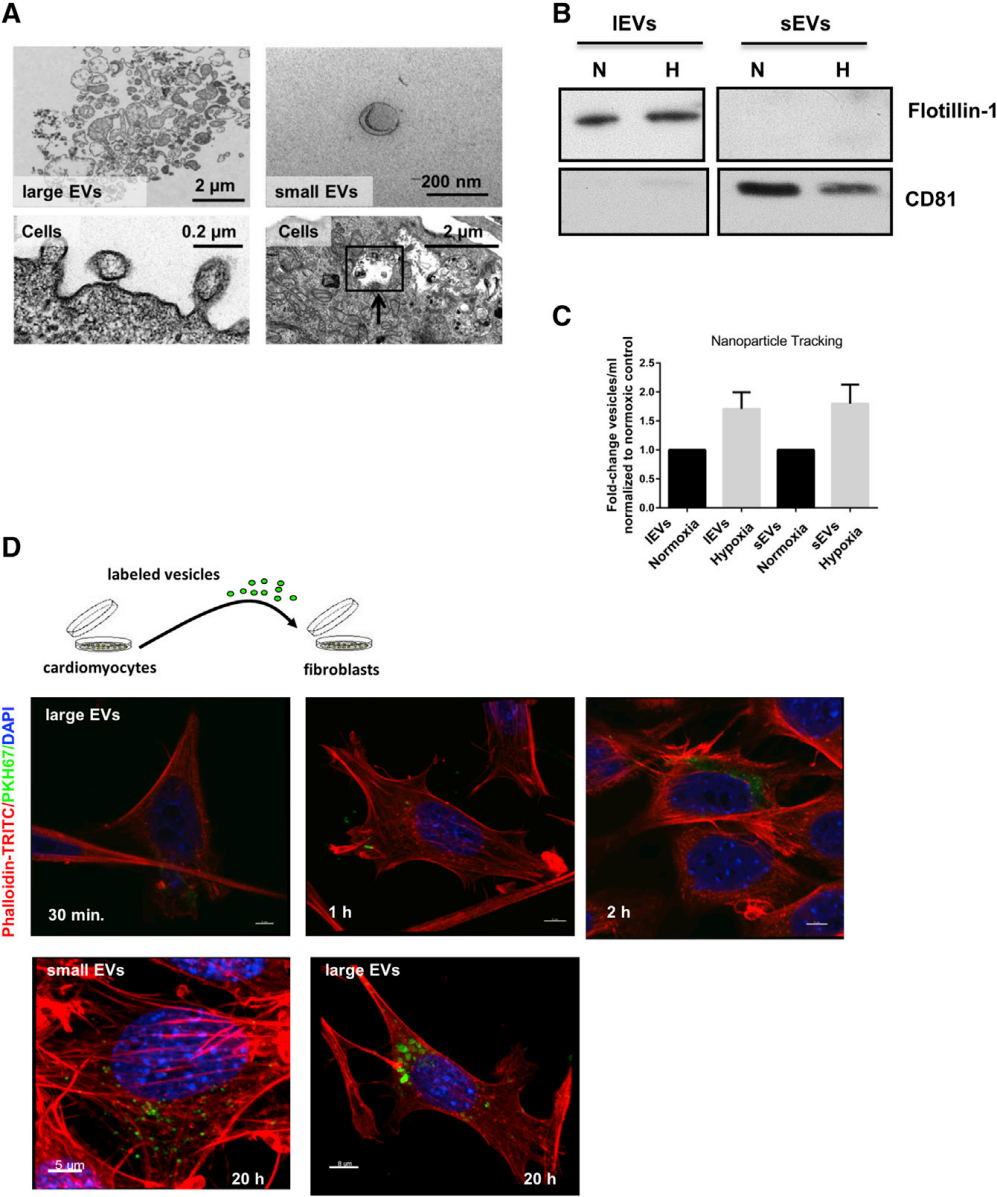
Cardiomyocytes Produce and Secrete EVs (A) Electron microscopy images of cardiomyocytes and purified cardiomyocyte-derived vesicles. Cytoplasm of cardiomyocytes with MVBs. The membrane of the MVB invaginated inward (black arrow), resulting in the formation of intraluminal vesicles. Outward budding of the plasma membrane led to the release of lEVs. The sEVs showed a cup-shaped structure with an approximate size of 100 nm, whereas the morphology of lEVs was rather diverse. (B) Western blot of isolated EVs derived from cardiomyocytes that were exposed to hypoxic (H) conditions for 24 h following 4 h reoxygenation or normoxic (N) conditions for 28 h. (C) Measurement of particle concentration using nanoparticle tracking analysis. n = 3 independent experiments, one-column t test. (D) EV uptake experiment. Isolated cardiomyocyte-derived lEVs were labeled with a green fluorescent dye (PKH67); co-cultured with fibroblasts for 30 min, 1 h, and 2 h at 37°C; and confocal images were taken. Fibroblasts were stained with DAPI (blue) and phalloidin-TRITC (red). After 20 h, sEVs and lEVs were internalized and located in the cytoplasm of fibroblasts. Scale bars, 5 μm. n = 3 independent experiments. Cardiomyocytes = cells.

Ischemia is well known to drive cardiac remodeling after MI. To understand a potential crosstalk between cardiomyocytes and fibroblasts via EVs and to mimic the clinical situation of ischemia/reperfusion *in vitro*, cardiomyocytes were exposed to hypoxic conditions followed by reoxygenation, and EVs—large EVs (lEVs) and small EVs (sEVs)—were isolated (nomenclature was according to Théry et al.^9^). By electron microscopy, we observed the formation of lEVs originating through the outward budding of the plasma membrane and the release of sEVs into the extracellular fluid (Figure 1A). We identified the appearance of typical multivesicular bodies (MVBs) enriched with intraluminal vesicles inside the cytoplasm, which were derived through the inward invagination of the MVB membrane (Figure 1A, black arrow). Further characterization of isolated EVs from the conditioned medium of cardiomyocytes demonstrated that sEVs were rounded in shape, with an approximate size of 100 nm in diameter, as shown by electron microscopy (Figure 1A) and expressed the marker protein CD81 (Figure 1B). In contrast, the lEV fraction displayed a multifaceted morphology and the presence of the characteristic marker Flotillin-1 (Figure 1B). Moreover, nanoparticle tracking analysis validated that hypoxia stimulated secretion of EVs (Figure 1C).

### Hypoxic Cardiomyocyte-Derived Vesicles Are Transferred to Fibroblasts, Leading to a Profibrotic Phenotype

Next, we investigated whether cardiomyocyte-derived vesicles would be transferred to and finally incorporated by fibroblasts. Secreted EVs—both small and large vesicles—were isolated, and subsequently, fluorescently labeled vesicles were incubated with fibroblasts. Uptake of vesicles into fibroblasts was analyzed by confocal imaging, revealing that both vesicle subtypes are internalized and located in the cytoplasm of fibroblasts in a time-dependent manner (Figure 1D; Figure S1). In addition, 3D reconstructions of the confocal image *Z* stacks confirmed that vesicles are taken up into fibroblasts and are not only attached to the cell surface (Video S1).

Video S1

**Figure 2:**
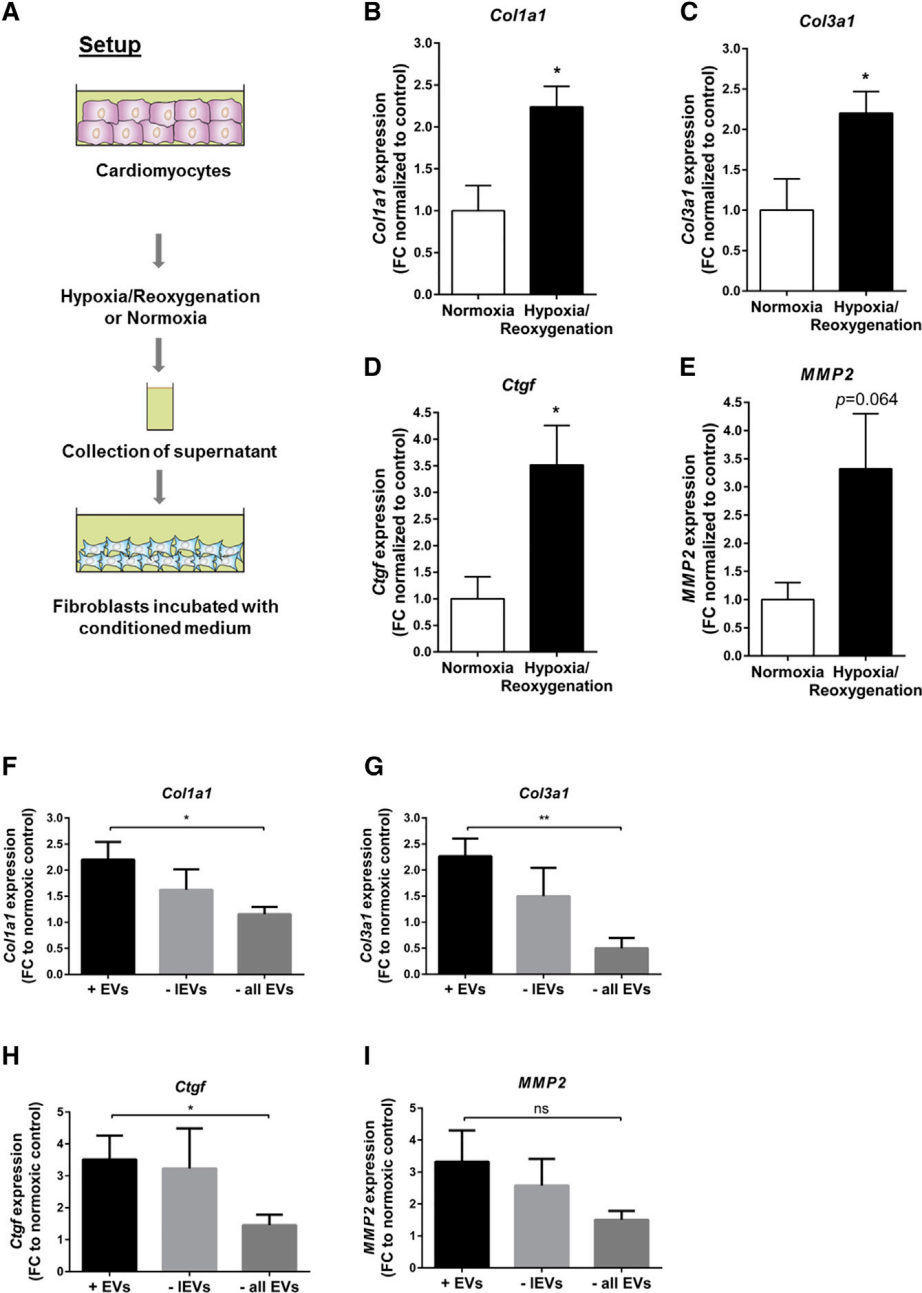
Cardiomyocyte-Derived Vesicles Are Taken Up into Fibroblasts, Leading to Profibrotic Transcriptome Changes (A) Setup to evaluate a potential vesicle-mediated crosstalk between hypoxic cardiomyocytes and fibroblasts. (B–E) Expression levels of fibrosis-related genes *Col1a1* (B), *Col3a1* (C), *Ctgf* (D), and *MMP2* (E) in fibroblasts after culture with hypoxic or normoxic conditioned medium. (F–I) Stepwise depletion of large vesicles (− lEVs) or all extracellular vesicles (−all EVs) out of the conditioned medium abolished the profibrotic phenotype: (F) *Col1a1*; (G) *Col3a1*; (H) *Ctgf*; and (I) *MMP2*. Data are presented as mean ± SEM. n = 4–5 independent experiments. *p ≤ 0.05; **p ≤ 0.01; ns, not significant; Student’s t test. FC, fold change to normoxic control.

Next to confocal imaging, a potential vesicle-mediated crosstalk between hypoxic cardiomyocytes and fibroblasts was further studied. The conditioned medium of cardiomyocytes was collected and incubated with fibroblasts (Figure 2A). We observed increased expression levels of the profibrotic marker genes *Col1a1*, *Col3a1*, *Ctgf*, and *MMP2* when fibroblasts were cultured with hypoxic conditioned medium compared to normoxic conditioned medium (Figures 2B–2E). Stepwise depletion of vesicles out of the medium abolished the profibrotic response in fibroblasts (Figures 2F–2I), indicating that cardiomyocyte-derived vesicles are a major factor in the fibrotic response.

### In Response to Hypoxia/Reoxygenation, lncRNAs Are Enriched in Cardiomyocyte-Derived Small and Large Vesicles

**Figure 3:**
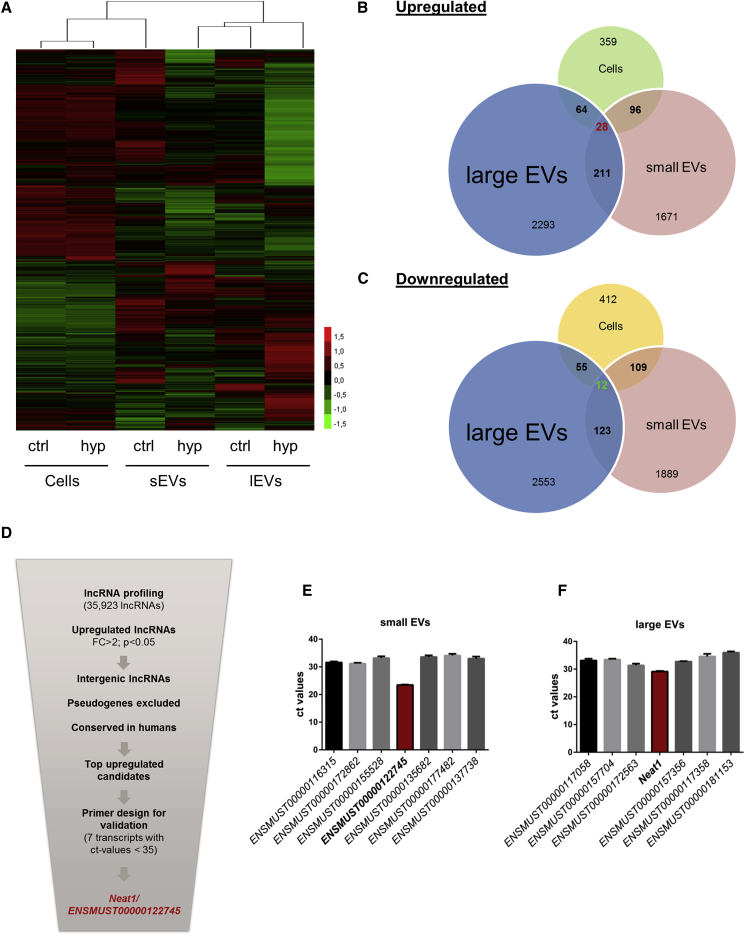
Deregulated lncRNAs in Extracellular Vesicles and Cardiomyocytes during Hypoxia/Reoxygenation (A) Heatmap of lncRNA mouse array in cardiomyocytes, sEVs, and lEVs exposed to hypoxic conditions for 24 h following 4 h of reoxygenation or normoxic conditions for 28 h. n = 3 independent experiments. (B and C) Different lncRNAs are (B) upregulated and (C) downregulated during hypoxia/reoxygenation in lEVs, sEVs, and cells, and only 12 (green, downregulated) or 28 (red, upregulated) are shared by all. (D–F) Scheme of the selection strategy (D) to identify lncRNA candidates in lEVs and sEVs leading to 2 candidates: Ensembl: *ENSMUST00000122745* in sEVs (E) and *Neat1* in lEVs (F). n = 3–6 independent experiments. Cells = cardiomyocytes; ctrl = normoxia; hyp = hypoxia/reoxygenation.

Non-coding RNAs have been shown to be crucial for the crosstalk between fibroblasts and cardiomyocytes.^10^ Here, we investigated changes of the vesicle-based lncRNA transcriptome during normoxia and hypoxia/reoxygenation in cardiomyocytes, as well as their secreted lEVs and sEVs. lncRNA profiling revealed that in both vesicle subtypes and cardiomyocytes, a large amount of lncRNAs were differentially expressed during hypoxia/reoxygenation (Figure 3A). Interestingly, when comparing regulated lncRNAs, only a small amount of lncRNAs—28 upregulated (indicated in red) and 12 downregulated (indicated in green)—showed a shared overlap between cardiomyocytes and both vesicle subtypes (Figures 3B and 3C). Suprisingly, only 135 downregulated or 239 upregulated lncRNAs are deregulated in both sEVs and lEVs, indicating a selective packaging and sorting mechanism of lncRNAs into specific vesicle subtypes. After *in silico* filtering according to stringent selection criteria, such as focus on upregulated lncRNAs which have intergenic localization and human conservation to translate findings in the future to patient data (Figure 3D), only lncRNAs with an abundancy over a certain threshold were considered for further analysis. We identified 7 deregulated lncRNA candidates in large vesicles as well as 7 potential candidates in the sEV fraction (Figures 3E and 3F). Subsequent studies were focused on two lncRNA candidates (Ensembl: *ENSMUST00000122745* and *Neat1*), since these lncRNAs exhibited the highest abundance in cardiac vesicles, suggesting a possible paracrine function.

**Figure 4:**
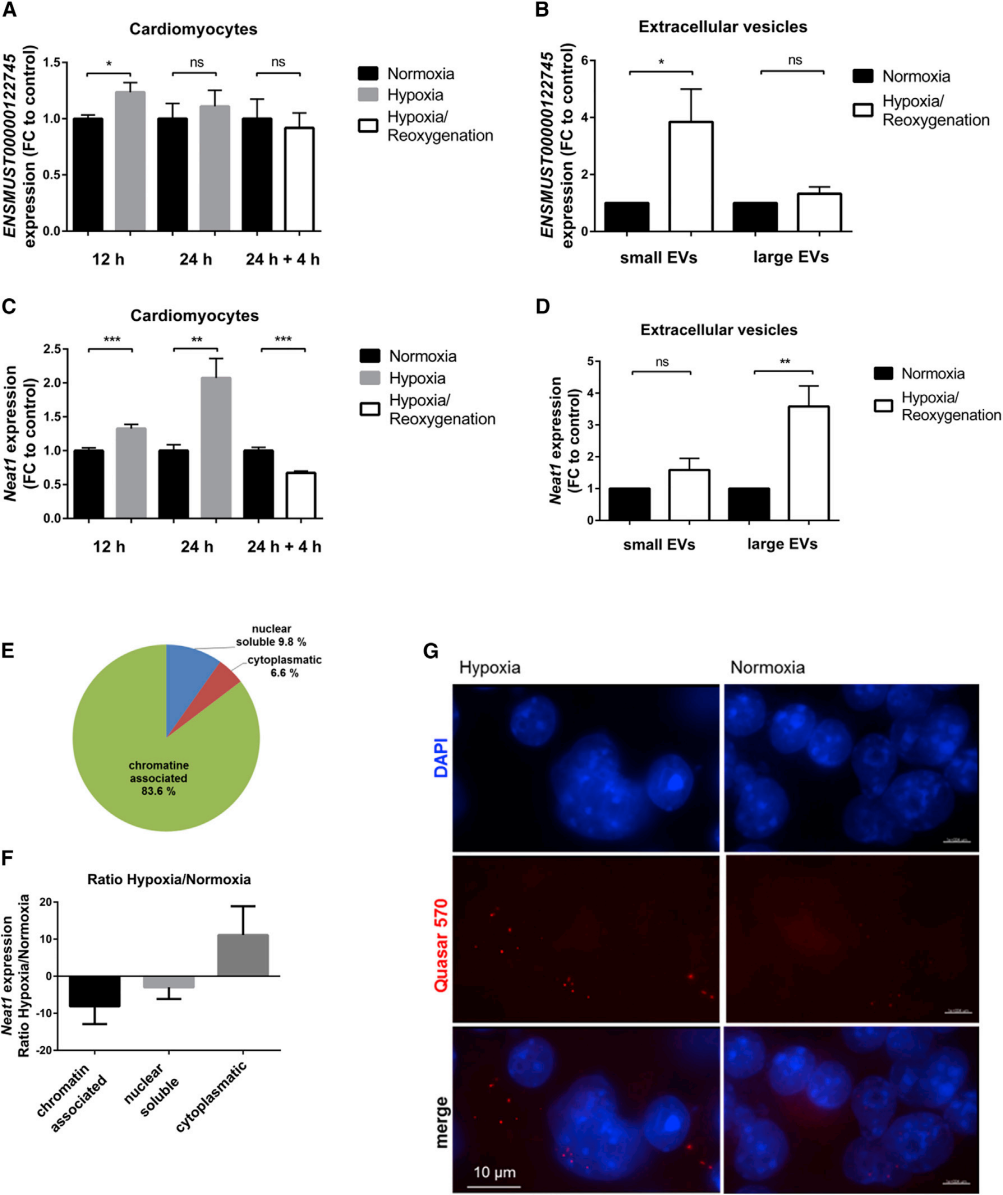
Characterization of Vesicle-Enriched lncRNAs (A and C) Gene expression levels of lncRNAs *ENSMUST00000122745* (A) and *Neat1* (C) in cardiomyocytes after 12 h and 24 h of hypoxia and 24 h of hypoxia following 4 h of reoxygenation. Data are presented as mean ± SEM; n = 3 independent experiments with 2–3 replicates per experiment. (B) *ENSMUST00000122745* is enriched in cardiomyocyte-derived small vesicles. Data are presented as mean ± SEM; n = 7–12 independent experiments. (D) Hypoxia/reoxygenation triggered the release of *Neat1* via internalization in mostly large vesicles. Data are presented as mean ± SEM; n = 6–7 independent experiments. (E) Distribution of *Neat1* expression levels in subcellular compartments of cardiomyocytes. Data represent percent distribution calculated to the complete amount of transcript in qRT-PCR analysis ± SEM (n = 3 independent experiments). (F) Ratio of *Neat1* expression levels in subcellular fractions of hypoxic and normoxic cardiomyocytes. n = 3 independent experiments. (G) Representative image of RNA-FISH of normoxic and hypoxic cardiomyocytes. Nuclei are stained with DAPI (blue); *Neat1* is stained with Stellaris FISH probe Quasar 570 (red). Scale bars, 10 μm. n = 3 independent experiments. *p ≤ 0.05; **p ≤ 0.01; ***p ≤ 0.001; ns, not significant, Student’s t test. FC, fold change of normoxic control.

After validation via qPCR, we measured the gene expression of these two selected candidates in hypoxic cardiomyocytes. We identified both lncRNAs to be hypoxia responsive. In particular, qRT-PCR experiments revealed increased expression levels of *ENSMUST00000122745* after 12 h of hypoxia in cardiomyocytes, whereas this effect was attenuated following reoxygenation (Figure 4A). In contrast, the expression level of *ENSMUST00000122745* was significantly increased in cardiomyocyte-derived sEVs after hypoxia/reoxygenation (Figure 4B), indicating that this lncRNA might be transported out of the cell via vesicle transfer, leading to reduced intracellular levels. Subcellular fractionation of cardiomyocytes showed that *ENSMUST00000122745* is not exclusively expressed in one specific compartment (Figure S2A), indicating various functional roles at different target sites. In addition, even though *ENSMUST00000122745* is expressed in the heart, it is also present in other organs, supporting this hypothesis (Figure S2B). *In vivo*, we confirmed the enrichment of *ENSMUST00000122745* in cardiac sEVs and depicted elevated expression levels in cardiac sEVs originated from infarcted hearts compared to sham control hearts (Figure S2C). As overexpression of this lncRNA had no impact on fibroblast function such as apoptosis, migration, or proliferation, we focused our study on a second lncRNA candidate, *Neat1*.

The lncRNA *Neat1* is composed of different transcript variants. First experiments indicated no changes in expression level between the different isoforms; thus, we decided to analyze total *Neat1* (data not shown). We identified *Neat1* to be hypoxia responsive in cardiomyocytes (Figure 4C). We detected significantly decreased expression levels following a short time of reoxygenation. In contrast, hypoxia/reoxygenation led to elevated *Neat1* levels in cardiac lEVs compared to normoxic vesicles and their secreting cells (Figure 4D). In addition, we found *Neat1* almost exclusively chromatin associated under baseline conditions (Figure 4E), pointing to a putative role as a transcriptional regulator of gene expression also in cardiac context. When comparing the amount of *Neat1* in subcellular fractions of hypoxic and normoxic cardiomyocytes, *Neat1* was shuttled to the cytoplasm during hypoxia, which could be indicative of a different function at the post-transcriptional level during hypoxic conditions (Figure 4F). Consistent with these findings, RNA-FISH (fluorescence *in situ* hybridization) analysis of cardiomyocytes confirmed a large amount of *Neat1* in the cytoplasm of the cell during hypoxia (Figure 4G), compared to normoxic control cells (Figure 4G). Additionally, we found *Neat1* ubiquitously expressed in different mouse organs, suggesting also extracardiac effects (Figure S3).

### *Neat1* Is Regulated by P53 and HIF2A

**Figure 5:**
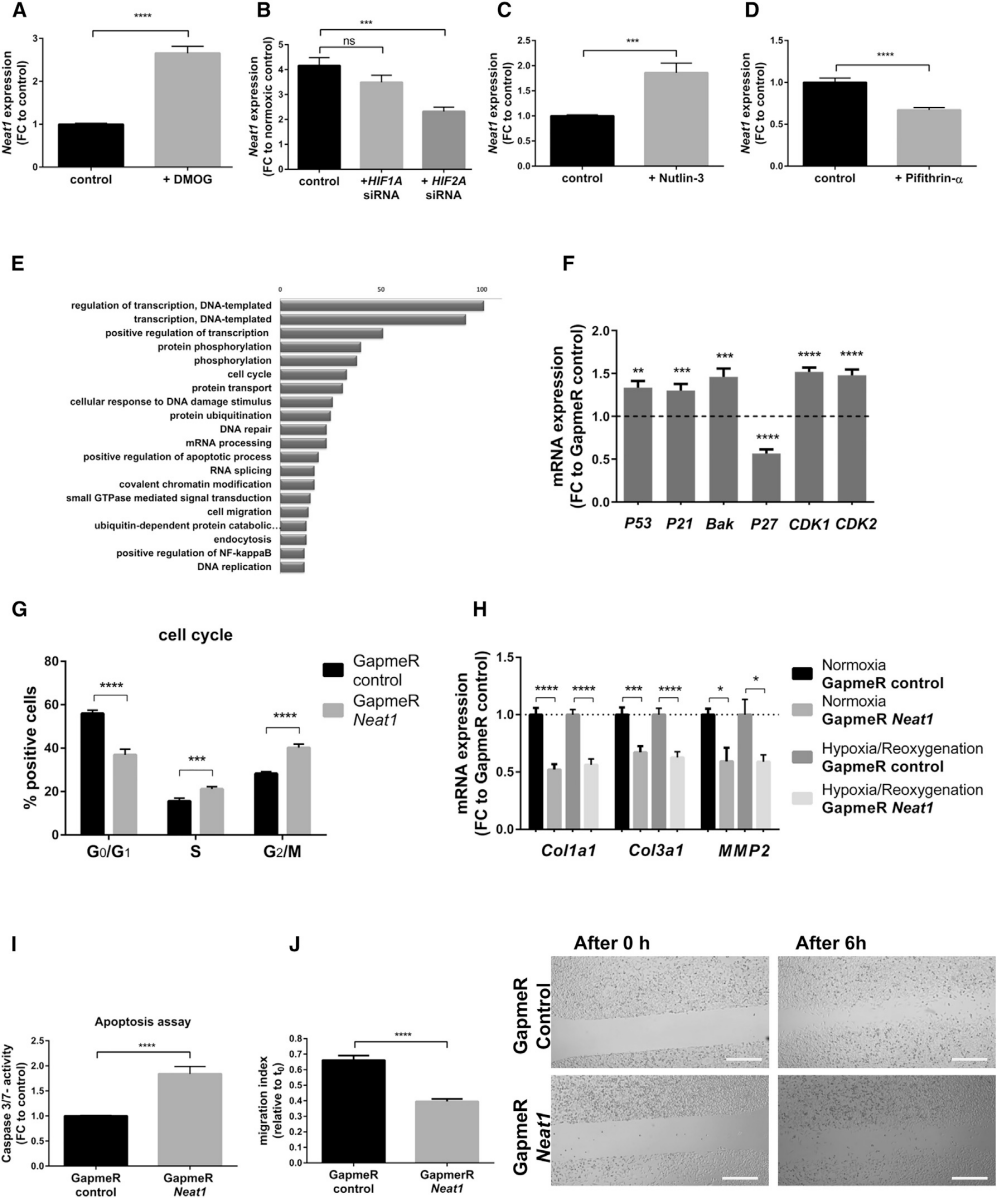
*Neat1* Inhibition Affects Fibroblast Function Regulation of *Neat1* expression under normoxic and hypoxic conditions in cardiomyocytes. (A) Expression level of *Neat1* in cardiomyocytes after treatment with 1 mM DMOG. (B) Gene expression of *Neat1* following siRNA-mediated silencing of *HIF1A* or *HIF2A* under hypoxic conditions in cardiomyocytes. Cells treated with a control siRNA served as controls. (C) Increased *Neat1* expression following treatment of cardiac fibroblasts with 10 μM Nutlin-3 (activator of P53). (D) Treatment of fibroblasts with a P53 inhibitor (Pifithrin-α, 10 μM) resulted in a significant decrease of *Neat1* expression levels. Downstream effects on target cells. (E) Gene set enrichment analysis of the significantly deregulated genes (adjusted p < 0.05) identified by RNA-seq. The Top 20 functional terms are displayed and sorted according to the gene counts belonging to a GOTERM annotation. (F) Expression levels of *P53*, *P21*, *Bak*, *P27*, *CDK1*, and *CDK2* mRNA in fibroblasts treated with *Neat1* GapmeR or control GapmeR. Data are presented as fold change to cells treated with GapmeR control. (G) Propidium iodide staining of fibroblasts treated for 48 h with GapmeR *Neat1* and GapmeR control to analyze the cell cycle using FACS. Plot indicates percentage of positive cells in the G_0_/G_1_ phase, S phase and G_2_/M phase. (H) Expression levels of profibrotic genes *Col1a1*, *Col3a1*, and *MMP2* in fibroblasts transfected with GapmeR control and GapmeR *Neat1* after exposure to hypoxic conditions for 24 h following 2 h reoxygenation or 26 h normoxic conditions. (I) Caspase-3/caspase-7 activity after treatment of 3T3 cells with GapmeR *Neat1* and GapmeR control for 48 h. (J) Representative pictures of fibroblasts treated with GapmeR *Neat1* or GapmeR control and scratched to determine migration capacity. Images show time points 0 h and 6 h after scratching. Left: migration index was calculated according to: (area [0 h] − area [6 h])/area (0 h). Scale bars, 500 μm. Data are presented as mean ± SEM. n = 3 independent experiments with 3 biological replicates per independent experiment (exception: *Neat1* expression after GapmeR treatment. n = 3 different wells). *p < 0.05; **p < 0.01; ***p ≤ 0.001; ****p ≤ 0.0001, Student’s t test (for A–D, F, and H–J) or one-way ANOVA for three groups with post hoc Tukey's multiple comparison test (for G). FC, fold change to control.

Because of its dominant role in hypoxic signaling, we tested a potential contribution of the hypoxia-inducible factor (HIF) in the ischemia-derived induction of *Neat1*. Previous reports described the human homolog of *Neat1* induced during hypoxia in human breast cancer cells and to be regulated by HIF-2A.^11^ However, the role of HIF in regulating *Neat1* expression in cardiac cells remains unknown. Treatment of cardiomyocytes with the HIF stabilizer dimethyloxalylglycine (DMOG) resulted in increased levels of *Neat1* **(**Figure 5A). We also transfected cardiac cells with small inhibitory RNAs (siRNAs) against different subunits of HIF and exposed the cells to normoxic and hypoxic conditions. The knockdown of *HIF1A* had no significant effect on *Neat1* expression, whereas inhibition of *HIF2A* resulted in decreased *Neat1* levels (Figure 5B). Besides the HIF-mediated regulation during hypoxia, *Neat1* is also highly abundant under normoxic conditions. Although HIF levels are very low under basal conditions, we examined a potential HIF-dependent regulation during normoxic conditions. Neither knockdown of *HIF1A* nor *HIF2A* isoform showed significant changes in *Neat1* expression levels (Figure S4). Recent studies revealed that the expression of human *Neat1* is regulated by P53.^12^ To evaluate whether P53 might regulate the expression during normoxic conditions, cells were treated first with Nutlin-3, an inducer of P53, which led to an increased *Neat1* expression. In contrast, a decrease in *Neat1* was observed after treatment of cardiomyocytes with an inhibitor of P53 (Pifithrin-α) **(**Figures 5C and 5D). These results confirm that P53 can transcriptionally regulate *Neat1* expression in cardiac cells.

### Silencing of *Neat1* Stalled Cell Cycle and Induced Apoptosis

We further characterized downstream effects of *Neat1* in fibroblasts. GapmeR-mediated knockdown of *Neat1* led to an ∼80% reduction in expression levels **(**Figure S5A). As we observed a strong effect of cell loss after *Neat1* knockdown *in vitro*, we decided to investigate apoptotic pathways in cardiac cells first. Indeed, *Neat1* inhibition increased caspase-3/caspase-7 activity in both fibroblasts (Figure 5I) and cardiomyocytes (Figure S6). Thus, we hypothesized an involvement of *Neat1* in signaling pathways during apoptosis events. Next to these observations, a transcriptome analysis of fibroblasts after *Neat1* silencing was performed to gain insights into genetic changes mediated by *Neat1* knockdown. We detected a massive deregulation of transcripts, and Gene ontology (GO) term enrichment analysis revealed multiple pathways fitting into the picture that *Neat1* is a central regulator of DNA damage and repair as well as cell cycle and apoptotic pathways (Figures 5E and S7). To assess an effect on the transcriptional regulation of apoptosis-related genes, expression of *P53* and P53 targets (*Bak* and *P21*) was analyzed and revealed increased levels after *Neat1* silencing (Figure 5F). As activated P53 has pleiotrophic effects such as cell-cycle signaling pathways and RNA sequencing (RNA-seq) showed an involvement in cell-cycle regulation, we tested a potential effect of *Neat1* silencing on cell-cycle progression. Fluorescence-activated cell sorting (FACS)-based detection validated the decreased level of cells in G_0_/G_1_ phase and increased levels in S and G_2_/M phase following *Neat1* inhibition, indicating impaired mitosis (Figure 5G). To obtain information on regulatory genes, mRNA levels of cell-cycle regulators such as *CDK1*, *CDK2*, and *P27* were measured. *Neat1* silencing resulted in decreased levels of the cell-cycle inhibitor *P27* and increased levels of cyclin-dependent kinase (CDK) *CDK1* and *CDK2* mRNA levels involved in cell-cycle progression (Figure 5F). To validate whether *Neat1* functions as a downstream effector of P53 and whether P53 inhibition can rescue the pro-apoptotic effect, we conducted *Neat1* modulation experiments with p53-deficient mouse embryonic fibroblasts (MEFs). In this cellular model, *Neat1* is also highly abundant, and its expression is, therefore, not entirely dependent on P53 abundance. Additionally, P53 deficiency did not completely attenuate apoptosis induction after *Neat1* inhibition, indicating that this is, in part, independent of P53 abundance in fibroblasts (Figure S8). In addition to genetic loss-of-function experiments, we targeted P53 with Pifithrin-α at the pharmacological level. This intervention also did not completely rescue the apoptotic effects (Figure S9) but underlined that therapeutic P53 targeting could mildly compensate for *Neat1* loss. Taken together, the data show that *Neat1* modulated the expression of P53 target genes, cell-cycle regulators and promoted cellular survival.

### *Neat1* Inhibition Decreased Profibrotic Genes

The key role of fibroblasts is to maintain the homeostasis of the extracellular matrix (ECM) and to control the production of ECM components.^13^ Global RNA-seq experiments indicated crucial participation of *Neat1* in fibrosis development, as we found deregulated levels of fibrotic-related genes such as *AKT3*, *collagens*, *matrix metalloproteinases*, *Timp2*, *transforming growth factor* (*TGF*)-*beta receptor*, *Lox*, or *Thrombospondin 2*. Thus, we investigated putative changes in the expression of fibrotic-related markers in fibroblasts after *Neat1* inhibition and knockdown of *Neat1* led to a significant decrease in the expression levels of *Col1a1*, *Col3a1*, and *MMP2*, indicating that *Neat1* is essential for fibrosis development (Figure 5H). Another prominent feature of fibroblasts is an endogenous migration capacity involved in wound healing. Applying a scratch assay revealed decreased migration capacity after *Neat1* inhibition (Figure 5J).

### *Neat1* Is Shuttled via Cardiomyocyte-Derived lEVs *In Vivo*

**Figure 6:**
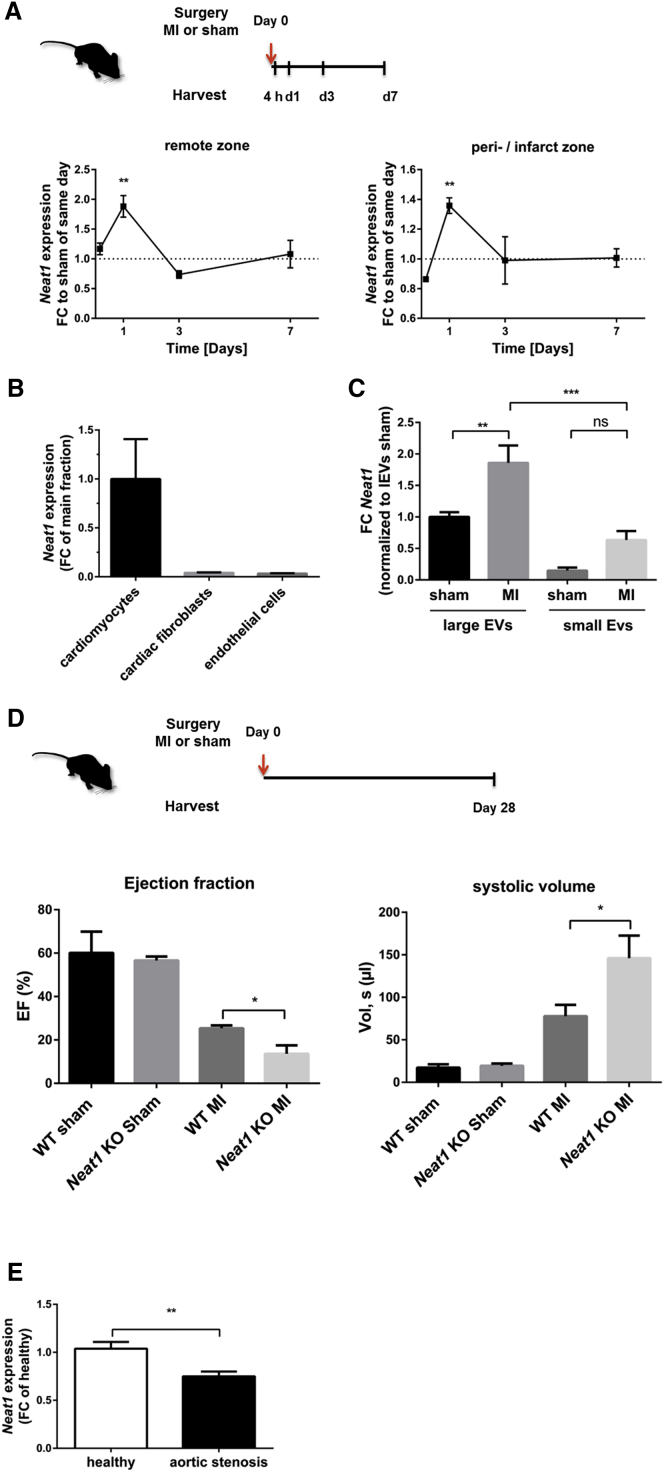
*Neat1* Is Transported via Large Vesicles *In Vivo* (A) Murine hearts from C57BL6/N mice were removed 4 h to 7 days after permanent left anterior descending artery (LAD) ligation and dissected into the remote and the peri-/infarct zones. Expression level of *Neat1* was measured in both zones. Data are presented as fold change (FC) to sham-operated mice of the same day ± SEM. n = 5–6 animals per group. (B) *Neat1* expression levels in fractionated hearts from C57BL6/N mice (n = 6). Data are presented as FC of main fraction ± SEM. (C) Expression level of *Neat1* in lEVs and sEVs isolated from mouse hearts 15 h after MI. n = 3–5 animals per group. Data are presented as fold change normalized to lEVs derived from sham hearts. (D) Murine hearts of *Neat1* KO or WT mice were removed 28 days after permanent LAD ligation or sham operation, and echocardiographic parameters were assessed. n = 3–5 animals per group. EF, ejection fraction. (E) Gene expression of *Neat1* in human heart tissue of aortic stenosis patients. n = 23. Healthy patients served as controls (n = 23). *p ≤ 0.05; **p ≤ 0.01; ***p ≤ 0.001; ns, not significant, Student’s t test.

To translate our *in vitro* findings, we studied *Neat1* abundance in different rodent models. We found *Neat1* highly enriched in cardiac myocytes (Figure 6B) and analyzed alterations in *Neat1* expression *in vivo* in a mouse model of MI. Consistent with our *in vitro* findings, *Neat1* is also deregulated in infarcted mouse hearts with a peak induction after a short time of ischemia (Figure 6A). Moreover, we confirmed that *Neat1* is also enriched in lEVs mainly originating from cardiomyocytes^14^ *in vivo* and upregulated in cardiac lEVs originated from infarcted hearts compared to sham-operated conditions (Figure 6C).

### *In Vivo* Relevance of *Neat1*

Next to the profiling of *Neat1* expression during cardiac ischemia, we studied the effects of a genetic *Neat1* loss in a murine setting of MI. Echocardiographic parameters in *Neat1* knockout (KO) mice in the context of MI underlined that knockdown of *Neat1* led to an impaired cardiac function with reduced ejection fraction and increased volume, presumably due to cardiomyocyte dysfunction 28 days (Figure 6D), as well as 14 days (Figure S10), post-MI. In addition, we assessed the percentages of fibrotic area in *Neat1* KO versus WT mice 28 days post-MI as well as sham-operated mice via Picrosirius red staining of left ventricles. We observed a strong trend toward higher fibrosis levels in *Neat1*-deficient mice compared to those in wild-type (WT) animals (Figure S11A). Supporting this finding, gene expression of fibrosis genes such as *Col1a1*, *Col3a1*, and *CTGF* was increased (Figures S11B–S11D). In agreement with these observations, we found that knockdown of *Neat1* displayed reduced contractile force recovery after hypoxia/reoxygenation in a humanized *ex vivo* model of engineered heart tissue (Figure S12).

Finally, we investigated whether human *Neat1* is of clinical relevance and measured its expression in hypertrophic myocardial tissue of aortic stenosis patients and showed that *Neat1* is significantly decreased compared to its expression in healthy hearts, suggesting that *Neat1* may serve as a future cardiovascular therapeutic target (Figure 6E).

## Discussion

Within the past years, small, nanosized vesicles (extracellular vesicles) have been identified as a new facet of microcommunication between different organs or cells.^10^, ^15^ Those vesicles gain more and more attention as paracrine effectors and are considered to serve both as novel clinical biomarkers and as novel targets for therapeutic drug development. Evidence is accumulating that EVs not only contain cellular degradation products but also are able to shuttle cell-specific signature cargoes such as ncRNAs to target cells under various physiological and pathological conditions, which highlights even more their therapeutic potential in various disease settings.^16^, ^17^, ^18^ Remarkably, a number of studies suggested that the unique cargo of proteins and microRNAs secreted extracellularly is required for the initiation and progression of cardiac remodeling and is capable of improving heart function. In addition, many studies reported that lncRNAs are actively secreted into the circulation during cardiac remodeling.^19^, ^20^, ^21^ Whether or not such lncRNAs are actively carried by myocardial secreted vesicles in the context of cardiac ischemia remains unknown.

In the present study, we identified a novel intercellular communication route between cardiomyocytes and fibroblasts mediated by the release of EVs in the context of hypoxia. This study also demonstrates that long non-coding RNAs are incorporated into vesicles and shuttled between cardiomyocytes and fibroblasts post-infarction and identifies an indispensable role for lncRNA *Neat1* in cardiac fibroblasts. These findings strengthen future perspectives for EVs to serve as endogenous carriers for therapeutic drugs or molecules such as lncRNAs enabling personalized treatment regimes.

Specifically, we showed that cardiomyocytes are able to secrete small and larger EVs and that hypoxia triggered the release of EVs. LncRNA profiling unraveled a large amount of dysregulated lncRNAs in cardiomyocytes, lEVs, and sEVs in response to hypoxia/reoxygenation. Notably, only a small amount was packed in both large and small vesicles, indicating a selective and not random sorting process of a specific lncRNA pattern into different vesicle populations. Finally, we identified two lncRNAs (*Neat1* and *ENSMUST00000122745*) enriched in different vesicle subtypes originating from cardiomyocytes. Whereas *ENSMUST00000122745* was rather sorted into small EVs, *Neat1* was almost exclusively transported via lEVs. In line with our *in vitro* results, we also evaluated that both lncRNAs are deregulated *in vivo* in infarcted mouse hearts and confirmed that, *in vivo*, *Neat1* is also shuttled via lEVs and that *ENSMUST00000122745* is shuttled via small vesicle transfer. Although evidence is accumulating that lncRNAs can be incorporated into EVs, the mechanism for how cells select specific lncRNAs for extracellular release remains unclear.

*Neat1* is a well-characterized lncRNA and was proven to play a major role in the formation of nuclear paraspeckles.^22^ In addition, several studies showed altered expression levels in human malignant diseases, including gastric cancer, lung cancer, and breast cancer.^11^, ^23^ As *Neat1* induction is associated with cellular stress conditions, it has been speculated that *Neat1* is involved in the cellular stress response. In our study, we identified *Neat1* to be a hypoxia-sensitive lncRNA in cardiomyocytes, in agreement with studies of human *Neat1* in cancer cells. Recent studies have identified a large number of lncRNAs such as *H19* and *HOTAIR* to be regulated by hypoxia, specifically by the transcription factor HIF.^24^, ^25^ In line with our results, a recent study reported *Neat1* regulation by HIF2A.^11^ Mechanistically, we could show here that *Neat1* is a downstream target of HIF2A under hypoxic conditions. Although both isoforms have the same binding site in the *Neat1* promoter region and are binding together in a complex to the HIF response elements, the hypoxic induction of *Neat1* is predominantly regulated by HIF2A. At the basal level, *Neat1* is also highly abundant. Recently, some reports explored a P53 dependency of *Neat1* expression for the human homolog and provided evidence for the binding of P53 to the promoter region.^12^, ^26^ Consistent with these studies, we observed that inactivation of P53 decreased *Neat1* expression and activation of elevated *Neat1* levels in murine cardiac cells. Some studies already provided evidence that lncRNAs not only are activated or suppressed by P53 but can also mediate the downstream effects of P53 by transcriptional regulation of target genes in the P53 pathways.^27^, ^28^ Therefore, we speculated on participation in P53 signaling routes. Indeed, we found higher levels of apoptosis in fibroblasts and cardiomyocytes as well as an increased gene expression of the proapoptotic factor *Bak* after *Neat1* knockdown. We next evaluated whether P53 deficiency could attenuate the intrinsic apoptotic signaling induced by *Neat1* loss. However, P53 loss did not completely rescue for the phenotype, indicating also independent regulatory mechanisms. Besides the pro-apoptotic consequences after *Neat1* silencing, we identified an involvement in cell-cycle progression. Cardiac cells arrest in the G_2_/M phase and do not complete or enter into mitosis, presumably via higher expression of cell-cycle regulators such as *CDK1* and *CDK2*.

We also evaluated the function of *Neat1* in fibroblasts via RNA-seq and confirmed that its inhibition strongly affects key fibroblast features such as response to DNA damage, DNA repair, apoptosis signaling, and fibrosis. We further validated the main characteristics and found that fibroblasts lose their ability to migrate and repress fibrotic-related genes upon silencing *Neat1*, suggesting that *Neat1* is essential for the expression of ECM components and the maintenance of heart function. In accordance with our results, recent studies observed that *Neat1* affects ECM protein secretion in mesangial cells^29^ and collagen expression in liver fibrosis.^30^ In-depth analysis of RNA-seq data revealed a key cell regulator, Akt, which can also be connected to fibrosis, as one of the top 10 deregulated genes after *Neat1* silencing. In line with this, we also detected several other genes that can be linked to AKT pathways to be deregulated, including phosphatidylinositol 3-kinase (PI3K) components and PTEN, indicating that *Neat1* controls subsequent downstream responses including cell survival, growth, fibrosis, proliferation, and migration via modulation of AKT activation. In agreement with this hypothesis, a recent work demonstrated that *Neat1* repression led to decreased proliferation and fibrosis in diabetic nephropathy via activation of the Akt/mTOR signaling pathway.^31^

To further investigate whether cardiomyocyte-derived vesicles are crucially involved in the paracrine action, we validated that the conditioned medium of hypoxic cardiomyocytes triggered a profibrotic response and that lncRNA-enriched cardiomyocyte-derived vesicles are taken up into fibroblasts. Whether the uptake and subsequent release of EVs to the cytoplasm contribute to cytoplasmic NEAT1 function still needs to be determined.

Finally, we evaluated the systemic function of *Neat1 in vivo* in a mouse model of MI. Cardiomyocyte apoptosis is a major event directly after MI, and, indeed, *Neat1* loss-of-function caused cardiomyocyte apoptosis *in vitro*. In line with this, *Neat1* expression was mainly increased 24 h after MI, indicating an involvement in both apoptosis and necrosis. Because we observed global impact on cardiac remodeling, including alteration of cardiomyocyte and fibroblast biology, we suggest studying a cardiomyocyte-specific KO for EV generation and outcome. Recently, it was also shown that *Neat1* can modulate immune cell function post-MI,^32^ highlighting another aspect of cardiac remodeling. Genetic loss of *Neat1* triggered a worsening of heart function in *Neat1* KO animals post-MI. A future therapeutic strategy would not be lowering cardiac *Neat1* level but, rather, overexpressing this lncRNA and applying *Neat1*-enriched EVs to the heart in a model of MI. Despite the fact that the research field of EVs has emerged in previous years from initial *in vitro* studies and pre-clinical reports to early clinical trials, we are currently still in the infancy of understanding the precise mechanism and their exact role. In addition, several milestones have to be overcome before entering clinical settings, such as optimizing isolation techniques, heart-specific delivery, and finding a suitable source of vesicles for large-scale production. However, as EVs have been shown to be capable of improving heart function as well as heart regeneration and are released into the circulation in patients, the use of vesicles as biomarkers or the therapeutic modulation by, e.g., drug loading into vesicles is very promising. In addition, several studies reported that *Neat1* can serve as a predictor of poor clinical outcome and might be an important prognostic biomarker in different cancer types.^33^, ^34^ We identified that human *Neat1* is of interest in clinical scenarios of cardiovascular diseases, as we detected decreased levels in aortic stenosis patients. Despite these findings, one has to keep in mind that these studies are only performed in a small cohort and have to be validated in larger settings. It would be also of great importance to determine the expression during the progression of the disease in order to evaluate the potential to serve as a predictor of poor survival or overall clinical outcome.

Collectively, this study provides a new concept of a paracrine cardiac communication system during hypoxic stress conditions mediated by lncRNA-enriched vesicles and opens a wide range of future diagnostic and therapeutic options.

## Materials and Methods

A detailed description of methods can be found in the Supplemental Materials and Methods.

### Cell-Culture Experiments

HL-1 cells, a murine atrial cardiac muscle cell line, were cultured in Claycomb medium (Sigma Aldrich, Munich, Germany) supplemented with 10% fetal bovine serum (FBS) (Sigma Aldrich, Munich, Germany), 1% penicillin/streptomycin (100 U/mL; 100 μg/mL; Sigma Aldrich), 0.1 mM norepinephrine (Sigma Aldrich, Munich, Germany), and 2 mM L-glutamine (Sigma Aldrich, Munich, Germany). NIH 3T3 mouse fibroblasts were cultured in DMEM supplemented with 10% FBS (Sigma Aldrich, Munich, Germany) and 1% penicillin/streptomycin (100 U/mL; 100 μg/mL; Sigma Aldrich, Munich, Germany). The cells were cultured in a humidified incubator with 21% O_2_ and 5% CO_2_ at 37°C. For loss-of-function studies, LNA GapmeRs (Exiqon, part of QIAGEN, Venlo, the Netherlands) against *Neat1* (5′-TACCATCAGCCTTTAG-3′) or a negative control (5′-AACACGTCTATACGC-3′) were used. NIH 3T3 cells were transiently transfected with 50 nM GapmeR using X-tremeGENE HP Transfection Reagent (Sigma-Aldrich, Munich, Germany). In order to evaluate the upstream mechanisms of *Neat1* expression, HL-1 cells were transfected with siRNA against HIF1A (Santa Cruz Biotechnology, Santa Cruz, CA, USA, # sc-35562), HIF2A (Thermo Fisher Scientific, #4390771, Darmstadt, Germany), or the corresponding negative controls using Lipofectamine 2000 (Invitrogen, Karlsruhe, Germany). For hypoxia experiments, the HL-1 cells were cultured in media supplemented with 5% exosome-depleted FBS after incubation in fully supplemented medium (10% FBS) for 24 h. To induce hypoxia, the cells were grown in a humidified incubator with 5% CO_2_ and 0.2% O_2_ at 37°C for 24 h following 4 h reoxygenation under normoxic conditions with 21% O_2_, if not indicated otherwise. The cells that served as a control group were cultured for the same time period at normoxic conditions.

### Extracellular Vesicle Isolation

The supernatant of HL-1 cells was collected, and extracellular vesicles were purified by differential centrifugation steps and ultracentrifugation. In brief, the conditioned medium was centrifuged at 300 × *g* for 10 min at 4°C to remove cellular debris, 2,000 × *g* for 20 min at 4°C to isolate apoptotic bodies, and 16,500 × *g* for 20 min at 4°C to purify lEVs. After the isolation of lEVs, the remaining supernatant was filtered through a 0.22-μm filter. For small EV isolation, the supernatant was additionally ultracentrifuged at 100,000 × *g* for 70 min. Protein content was measured using the MicroBCA protein assay (Thermo Fisher Scientific, Darmstadt, Germany). The lEVs and sEVs were analyzed by western blot as previously described, and nanoparticle tracking analysis was carried out using the LM10 unit (Nanosight). For uptake experiments, EVs were labeled with the PKH67 Green Fluorescent Cell Linker Kit (Sigma-Aldrich, Munich, Germany) according to the manufacturer’s protocol, with minor modifications. Briefly, 7.5 μg of an EV subtype was mixed with 1 mL diluent C. Then, 4 μL PKH67 dye was added to 1 mL diluent C (2× dye solution) and gently mixed with the EV/diluent C solution and incubated for 4 min. Subsequently, 1 mL 0.5% BSA in PBS was added and incubated for 5 min to allow binding of the dye. The solution was centrifuged for 70 min at 100,000 × *g* (for sEVs) or 20 min at 16,500 × *g* (for lEVs), and the pellet was diluted in cell-culture medium. 7.5 μg EVs were used for further experiments.

### Animal Studies

Animal studies were performed in accordance with the relevant guidelines and regulations of the responsible authorities (governmental animal ethics committee LAVES). For all animal experiments, we used 8- to 10-week-old male C57Bl6 mice. *Neat1* KO have been described previously.^35^

### Permanent Ligation of LAD (Myocardial Infarction)

Mice were anesthetized by 2%–3% isoflurane mixed with O_2_ in an induction chamber. The neck and chest area was shaved and disinfected with betadine and alcohol. Mice were intubated via intratracheal cannula and fixed in the supine position to a heating pad (temperature was maintained at 37°C) and under an operating microscope. The trachea cannula was then attached to a small animal respirator, and the animal was ventilated at 100/min with a 150-μL stroke volume. After adequate analgesia (Torbugesic Vet [Butorphanol] and Novalgin [Metamizol]), a horizontal skin incision approximately 0.5–1.0 cm in length was made laterally over the second and third ribs. After thoracotomy, the thyroid and lung were retracted, allowing for visualization of the anterior wall of the left ventricle under low-power magnification. A 7/0 silk suture was inserted in the myocardium and passed under the left anterior descending branch of the left anterior descending artery (LAD), and the suture was tied around. Significant color changes at the ischemic area were considered indicative of successful coronary occlusion. The sham procedure was identical, except that the coronary vessel was not ligated.

### Statistics

All experiments were performed as described in the corresponding figure legends. In general, *in vitro* experiments were performed in 3 independent experiments with 3 biological replicates/wells per independent experiment (n = 3) unless stated otherwise. Data are presented as mean of independent experiments/independent samples ± SEM. GraphPad Prism 6 (GraphPad Software) was used to perform statistical analysis. For statistical comparison of two groups, unpaired two-tailed Student’s t test was used. For comparison of three or more groups, a one-way ANOVA followed by Tukey’s post-test was applied. A p value of 0.05 or lower was considered to be significant in all experiments.

### Human Tissue Sampling

RNA was isolated from human cardiac tissue of the left ventricle from patients subjected to aortic valve replacement or from healthy donor hearts. For this study, expression of the gene of interest was measured in 23 aortic stenosis patients (male:female, 15:8; mean age, 69.65 ± 17.48) and 23 healthy patients (male:female, 14:9; mean age, 38.61 ± 12.96). Approval of the study was given by the institutional committees of the University of Würzburg, Würzburg, Germany, and the University of Hamburg, Hamburg, Germany.

## Author Contributions

T.T., J.F., and C.B.: conception, design and interpretation of the data, and revision of the manuscript; F.K.: conception, data analysis and interpretation, and drafting the manuscript; K.X., C.M.B., X.L., B.S., S.H.-B., A.F., M.N.H., T.E., S.F., C.G., K.S., A.J., A.P., S.D., S.M.R.-B., S.S., S.M., and K.S.: analysis and interpretation of data and final approval of the manuscript.

## Conflict of Interests

T.T. is a founder of and holds shares in Cardior Pharmaceuticals. The other authors report no competing interests.

## Supplemental Information

Document S1. Figures S1–S12 and Supplemental Materials and Methods

Document S2. Article plus Supplemental Information

## References

[1] Dobaczewski M., Gonzalez-Quesada C., Frangogiannis N.G. (2010). The extracellular matrix as a modulator of the inflammatory and reparative response following myocardial infarction. J. Mol. Cell. Cardiol..

[2] Frantz S., Bauersachs J., Ertl G. (2009). Post-infarct remodelling: contribution of wound healing and inflammation. Cardiovasc. Res..

[3] Burchfield J.S., Xie M., Hill J.A. (2013). Pathological ventricular remodeling: mechanisms: part 1 of 2. Circulation.

[4] Takeda N., Manabe I. (2011). Cellular interplay between cardiomyocytes and nonmyocytes in cardiac remodeling. Int. J. Inflamm..

[5] Kakkar R., Lee R.T. (2010). Intramyocardial fibroblast myocyte communication. Circ. Res..

[6] Kuwabara Y., Ono K., Horie T., Nishi H., Nagao K., Kinoshita M., Watanabe S., Baba O., Kojima Y., Shizuta S. (2011). Increased microRNA-1 and microRNA-133a levels in serum of patients with cardiovascular disease indicate myocardial damage. Circ. Cardiovasc. Genet..

[7] Cheng Y., Wang X., Yang J., Duan X., Yao Y., Shi X., Chen Z., Fan Z., Liu X., Qin S. (2012). A translational study of urine miRNAs in acute myocardial infarction. J. Mol. Cell. Cardiol..

[8] Bang C., Batkai S., Dangwal S., Gupta S.K., Foinquinos A., Holzmann A., Just A., Remke J., Zimmer K., Zeug A. (2014). Cardiac fibroblast-derived microRNA passenger strand-enriched exosomes mediate cardiomyocyte hypertrophy. J. Clin. Invest..

[9] Théry C., Witwer K.W., Aikawa E., Alcaraz M.J., Anderson J.D., Andriantsitohaina R., Antoniou A., Arab T., Archer F., Atkin-Smith G.K. (2018). Minimal information for studies of extracellular vesicles 2018 (MISEV2018): a position statement of the International Society for Extracellular Vesicles and update of the MISEV2014 guidelines. J. Extracell. Vesicles.

[10] Bang C., Antoniades C., Antonopoulos A.S., Eriksson U., Franssen C., Hamdani N., Lehmann L., Moessinger C., Mongillo M., Muhl L. (2015). Intercellular communication lessons in heart failure. Eur. J. Heart Fail..

[11] Choudhry H., Albukhari A., Morotti M., Haider S., Moralli D., Smythies J., Schödel J., Green C.M., Camps C., Buffa F. (2015). Tumor hypoxia induces nuclear paraspeckle formation through HIF-2α dependent transcriptional activation of NEAT1 leading to cancer cell survival. Oncogene.

[12] Blume C.J., Hotz-Wagenblatt A., Hüllein J., Sellner L., Jethwa A., Stolz T., Slabicki M., Lee K., Sharathchandra A., Benner A. (2015). p53-dependent non-coding RNA networks in chronic lymphocytic leukemia. Leukemia.

[13] Kong P., Christia P., Frangogiannis N.G. (2014). The pathogenesis of cardiac fibrosis. Cell. Mol. Life Sci..

[14] Loyer X., Zlatanova I., Devue C., Yin M., Howangyin K.Y., Klaihmon P., Guerin C.L., Kheloufi M., Vilar J., Zannis K. (2018). Intra-cardiac release of extracellular vesicles shapes inflammation following myocardial infarction. Circ. Res..

[15] Adamiak M., Sahoo S. (2018). Exosomes in myocardial repair: advances and challenges in the development of next-generation therapeutics. Mol. Ther..

[16] Viereck J., Thum T. (2017). Circulating noncoding RNAs as biomarkers of cardiovascular disease and injury. Circ. Res..

[17] Valadi H., Ekström K., Bossios A., Sjöstrand M., Lee J.J., Lötvall J.O. (2007). Exosome-mediated transfer of mRNAs and microRNAs is a novel mechanism of genetic exchange between cells. Nat. Cell Biol..

[18] Sahoo S., Losordo D.W. (2014). Exosomes and cardiac repair after myocardial infarction. Circ. Res..

[19] Kumarswamy R., Bauters C., Volkmann I., Maury F., Fetisch J., Holzmann A., Lemesle G., de Groote P., Pinet F., Thum T. (2014). Circulating long noncoding RNA, LIPCAR, predicts survival in patients with heart failure. Circ. Res..

[20] Vausort M., Wagner D.R., Devaux Y. (2014). Long noncoding RNAs in patients with acute myocardial infarction. Circ. Res..

[21] Yang Y., Cai Y., Wu G., Chen X., Liu Y., Wang X., Yu J., Li C., Chen X., Jose P.A. (2015). Plasma long non-coding RNA, CoroMarker, a novel biomarker for diagnosis of coronary artery disease. Clin. Sci. (Lond).

[22] Hirose T., Virnicchi G., Tanigawa A., Naganuma T., Li R., Kimura H., Yokoi T., Nakagawa S., Bénard M., Fox A.H., Pierron G. (2014). NEAT1 long noncoding RNA regulates transcription via protein sequestration within subnuclear bodies. Mol. Biol. Cell.

[23] Ma Y., Liu L., Yan F., Wei W., Deng J., Sun J. (2016). Enhanced expression of long non-coding RNA NEAT1 is associated with the progression of gastric adenocarcinomas. World J. Surg. Oncol..

[24] Wu W., Hu Q., Nie E., Yu T., Wu Y., Zhi T., Jiang K., Shen F., Wang Y., Zhang J., You Y. (2017). Hypoxia induces H19 expression through direct and indirect Hif-1α activity, promoting oncogenic effects in glioblastoma. Sci. Rep..

[25] Zhou C., Ye L., Jiang C., Bai J., Chi Y., Zhang H. (2015). Long noncoding RNA HOTAIR, a hypoxia-inducible factor-1α activated driver of malignancy, enhances hypoxic cancer cell proliferation, migration, and invasion in non-small cell lung cancer. Tumour Biol..

[26] Adriaens C., Standaert L., Barra J., Latil M., Verfaillie A., Kalev P., Boeckx B., Wijnhoven P.W., Radaelli E., Vermi W. (2016). p53 induces formation of NEAT1 lncRNA-containing paraspeckles that modulate replication stress response and chemosensitivity. Nat. Med..

[27] Huarte M., Guttman M., Feldser D., Garber M., Koziol M.J., Kenzelmann-Broz D., Khalil A.M., Zuk O., Amit I., Rabani M. (2010). A large intergenic noncoding RNA induced by p53 mediates global gene repression in the p53 response. Cell.

[28] Chaudhary R., Gryder B., Woods W.S., Subramanian M., Jones M.F., Li X.L., Jenkins L.M., Shabalina S.A., Mo M., Dasso M. (2017). Prosurvival long noncoding RNA *PINCR* regulates a subset of p53 targets in human colorectal cancer cells by binding to Matrin 3. eLife.

[29] Wang X., Xu Y., Zhu Y.C., Wang Y.K., Li J., Li X.Y., Ji T., Bai S.J. (2019). LncRNA NEAT1 promotes extracellular matrix accumulation and epithelial-to-mesenchymal transition by targeting miR-27b-3p and ZEB1 in diabetic nephropathy. J. Cell. Physiol..

[30] Yu F., Jiang Z., Chen B., Dong P., Zheng J. (2017). NEAT1 accelerates the progression of liver fibrosis via regulation of microRNA-122 and Kruppel-like factor 6. J. Mol. Med. (Berl.).

[31] Huang S., Xu Y., Ge X., Xu B., Peng W., Jiang X., Shen L., Xia L. (2019). Long noncoding RNA NEAT1 accelerates the proliferation and fibrosis in diabetic nephropathy through activating Akt/mTOR signaling pathway. J. Cell. Physiol..

[32] Gast M., Rauch B., Haghikia A., Nakagawa S., Haas J., Stroux A., Schmidt D., Schumann P., Weiss S., Jensen L. (2019). Long noncoding RNA NEAT1 modulates immune cell functions and is suppressed in early onset myocardial infarction patients. Cardiovasc. Res..

[33] Ning L., Li Z., Wei D., Chen H., Yang C. (2017). LncRNA, NEAT1 is a prognosis biomarker and regulates cancer progression via epithelial-mesenchymal transition in clear cell renal cell carcinoma. Cancer Biomark..

[34] Wu Y., Yang L., Zhao J., Li C., Nie J., Liu F., Zhuo C., Zheng Y., Li B., Wang Z., Xu Y. (2015). Nuclear-enriched abundant transcript 1 as a diagnostic and prognostic biomarker in colorectal cancer. Mol. Cancer.

[35] Nakagawa S., Naganuma T., Shioi G., Hirose T. (2011). Paraspeckles are subpopulation-specific nuclear bodies that are not essential in mice. J. Cell Biol..

