# Supplementary material for: Long Noncoding RNA-Enriched Vesicles Secreted by Hypoxic Cardiomyocytes Drive Cardiac Fibrosis
Source: Mol Ther Nucleic Acids. 2019 Sep 13;18:363–74. doi: 10.1016/j.omtn.2019.09.003 (PMC6807307; doi:10.1016/j.omtn.2019.09.003)
Supplement: Document S1. Figures S1–S12 and Supplemental Materials and Methods [file mmc1.pdf]

OMTN, Volume *18*

Supplemental Information

Long Noncoding RNA-Enriched Vesicles Secreted by Hypoxic Cardiomyocytes Drive Cardiac Fibrosis

Franziska Kenneweg, Claudia Bang, Ke Xiao, Chantal M. Boulanger, Xavier Loyer, Stephane Mazlan, Blanche Schroen, Steffie Hermans-Beijnsberger, Ariana Foinquinos, Marc N. Hirt, Thomas Eschenhagen, Sandra Funcke, Stevan Stojanovic, Celina Genschel, Katharina Schimmel, Annette Just, Angelika Pfanne, Kristian Scherf, Susann Dehmel, Stella M. Raemon-Buettner, Jan Fiedler, and Thomas Thum

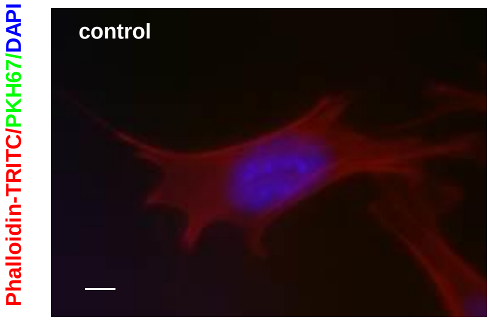

Figure 1: Microscope image of fibroblasts. Vehicle control (PBS) was stained with a green fluorescent dye (PKH67), co-cultured with fibroblasts for 20h at 37 °C and images were taken. Fibroblasts were stained with DAPI (blue) and Phalloidin-TRITC (red). Scale bar=5 µm

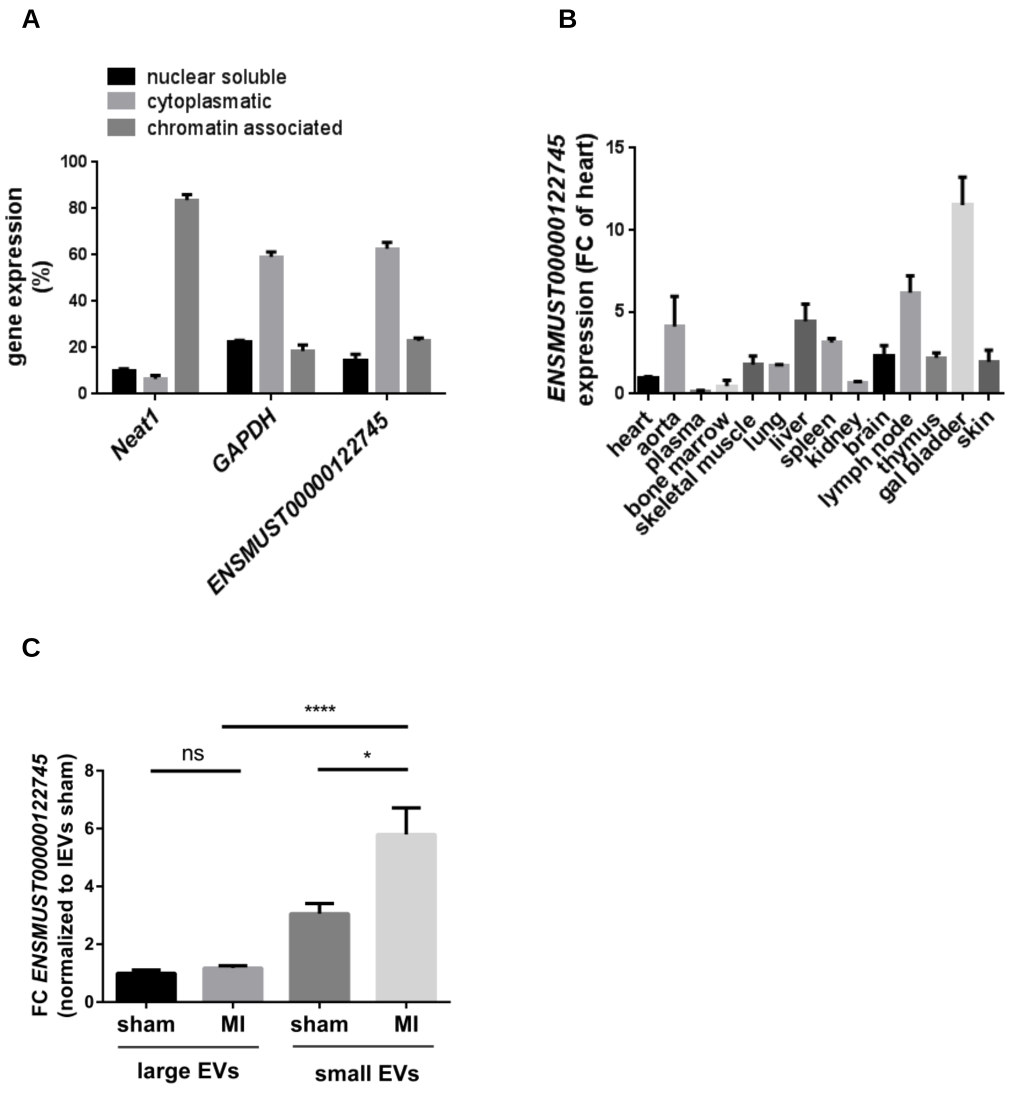

Figure 2: Characterization of lncRNA *ENSMUST00000122745*

(**A**) Distribution of *ENSMUST00000122745* expression levels in subcellular compartments of cardiomyoctes. Data are % distribution calculated to the complete amount of transcript in qRT-PCR analysis ± SEM (n=3 independent experiments). (**B**) Gene expression of *ENSMUST00000122745* in murine organs of C57BL6 mice. Data are presented as mean ±SEM. N=3 animals. (**C**) Gene expression of *ENSMUST00000122745* in large and small Evs isolated from mouse hearts 15 h after myocardial infarction (MI) was quantified by qRT-PCR. n=3 to 5 animals per group. Data are presented as fold change normalized to microvesicles derived from sham hearts. FC=foldchange * $p \leq 0.05$; **** $p \leq 0.0001$ Student´s t-test

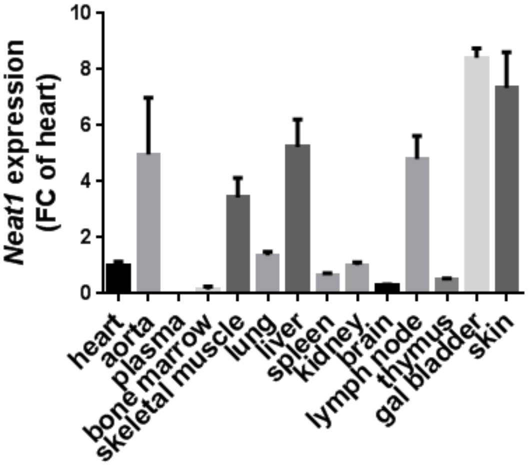

Figure 3: Characterization of *Neat1*

Distribution of *Neat1* in murine organs of C57BL6 mice. Data are presented as mean ±SEM. N=3 animals. FC=fold change of heart

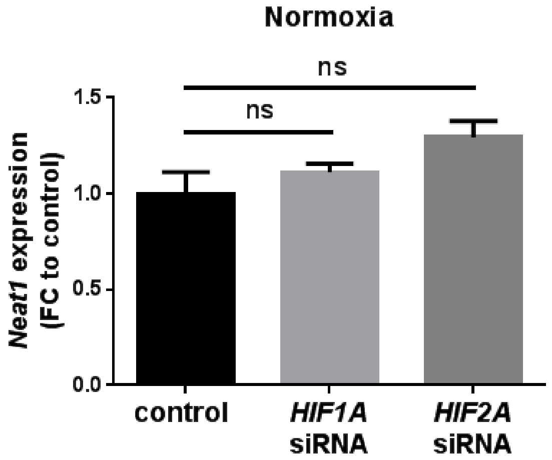

Figure 4: Regulation of *Neat1* expression

Gene expression level of *Neat1* following siRNA-mediated silencing of HIF1A or HIF2A under normoxic conditions (21% O_2) in cardiomyocytes. Data are presented as mean ±SEM. N=3

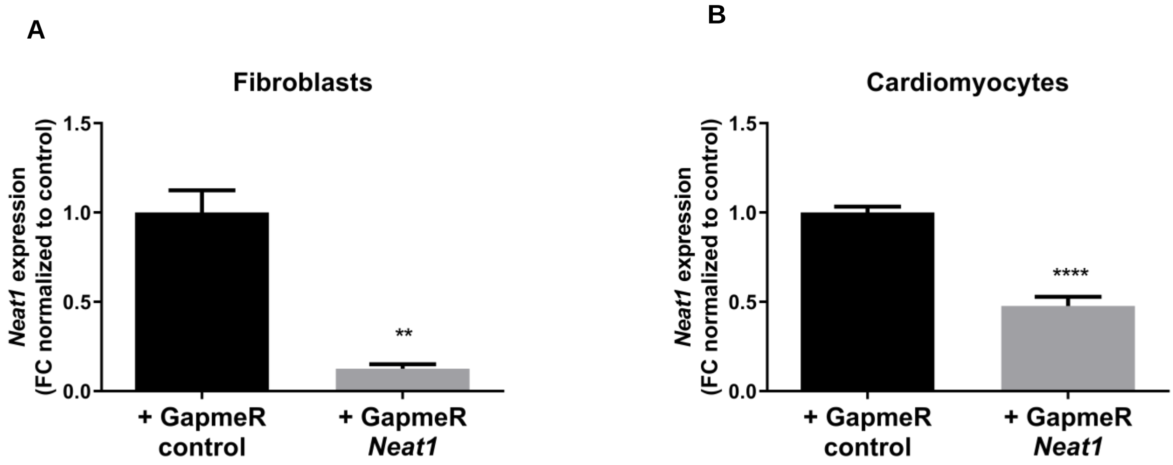

Figure 5: Knockdown efficiency of *Neat1*. Expression levels of Neat1 in cardiomyocytes (A) and fibroblasts (B) treated with GapmeR *Neat1* or control GapmeR. Data are presented as mean fold change ±SEM. N=3; ** $p \leq 0.01$; **** $p \leq 0.0001$ Student´s t-test

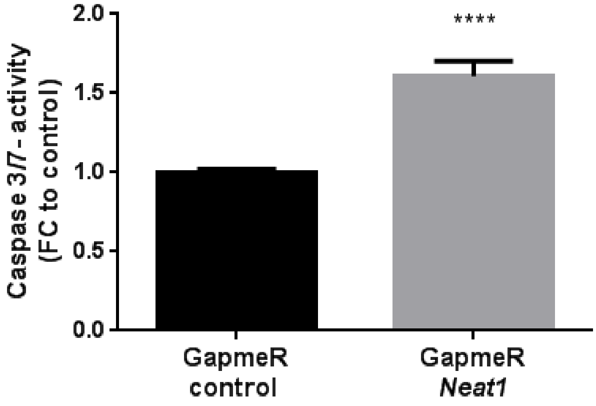

Figure 6: Apoptosis levels in cardiomyocytes
Caspase 3/7-activity was measured after treatment of cardiomyocytes with GapmeR *Neat1* and GapmeR control. Data are presented as mean ±SEM. N=3. **** p≤0.0001 Student´s t-test

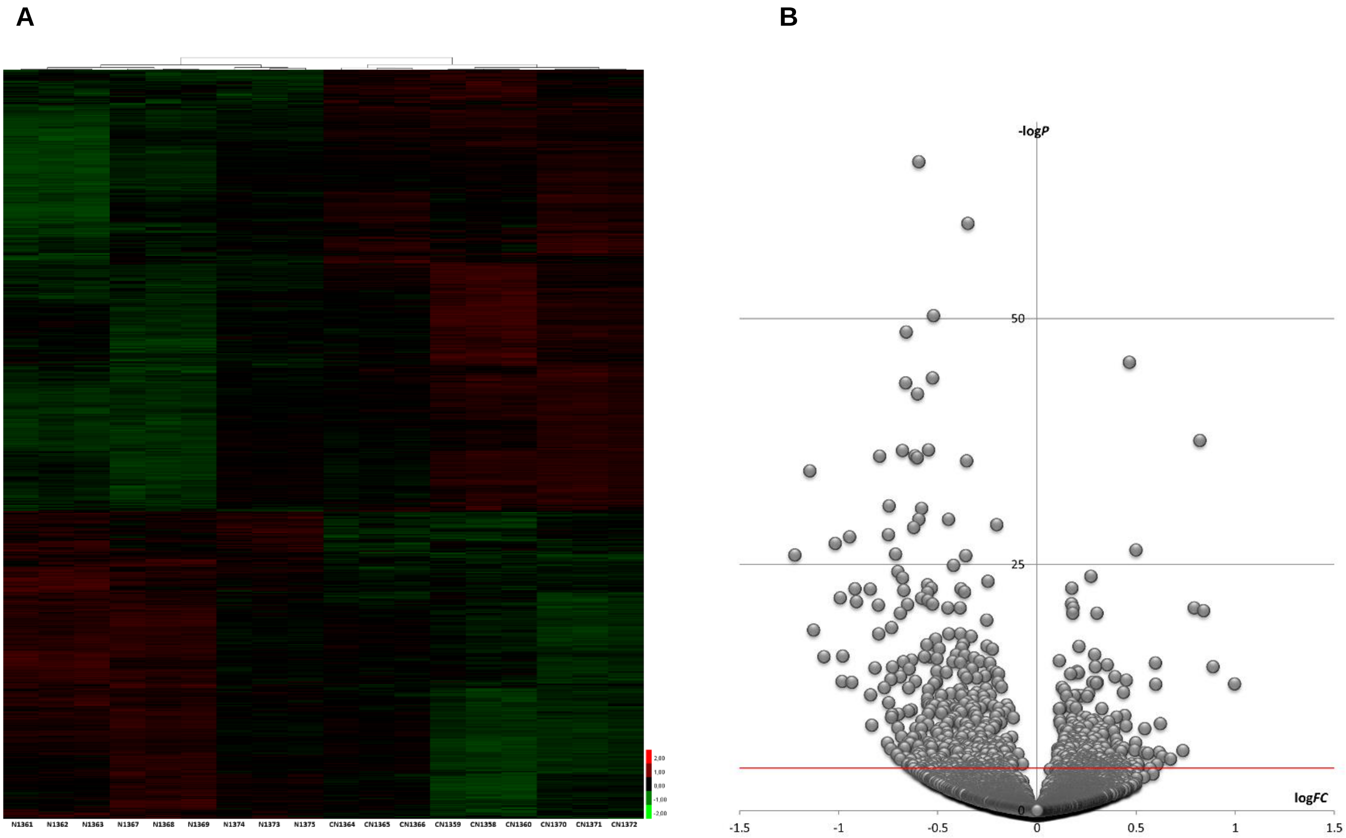

Figure 7: RNA-Sequencing data.

(A) Heatmap of the normalized expression level of the significantly regulated genes (padj<0.05) in fibroblasts treated with GapmeR control or GapmeR *Neat1*. n=3 independent experiment with 3 biological replicates. (**B**) Volcano plot of the differential expression analysis results from RNA-seq data. X-axis: log transformed fold change of the gene expression level between Gapmer *Neat1* treated samples and GapmeR controls. Y-axis: log transformed adjusted p-value.

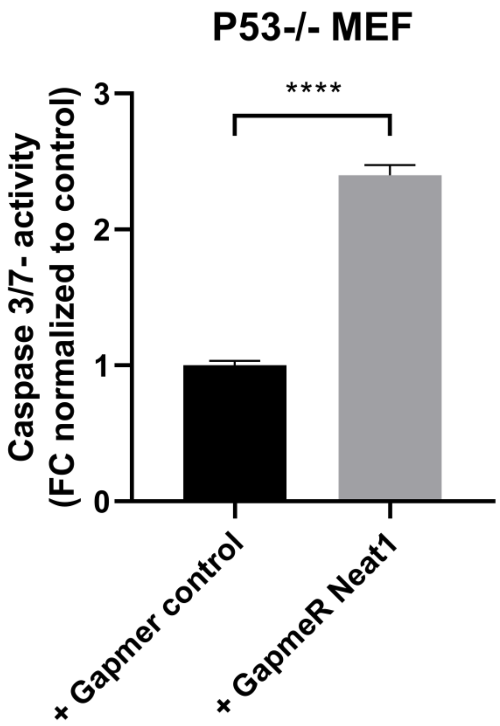

Figure 8: Apoptosis assessment in P53-/- MEFs

Caspase 3/7-activity was measured after treatment of P53-deficient mouse embryonic fibroblasts (MEF) with GapmeR *Neat1* and GapmeR control. Data are presented as mean ±SEM. N=3 independent experiments with 3 biological replicates. **** p≤0.0001; Student´s t-test

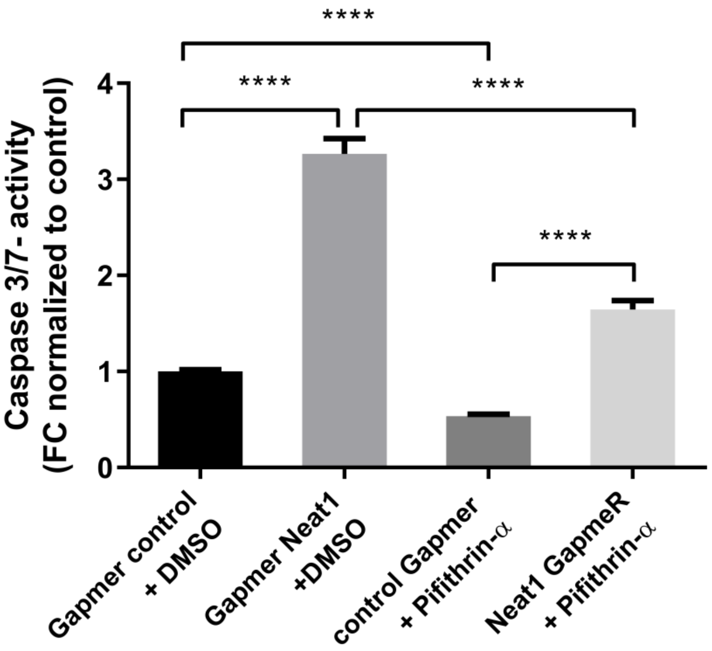

Figure 9: Apoptosis induction by *Neat1* loss is in part P53-independent

Caspase-3/7 activity was measured after treatment of 3T3 cells with GapmeR control and dimethyl sulphoxide (DMSO)/Pifithrin-α and GapmeR *Neat1* and DMSO/Pifithrin-α. Data are presented as mean ±SEM. N=3 independent experiments with 3 biological replicates. **** $p \leq 0.0001$; One-way ANOVA

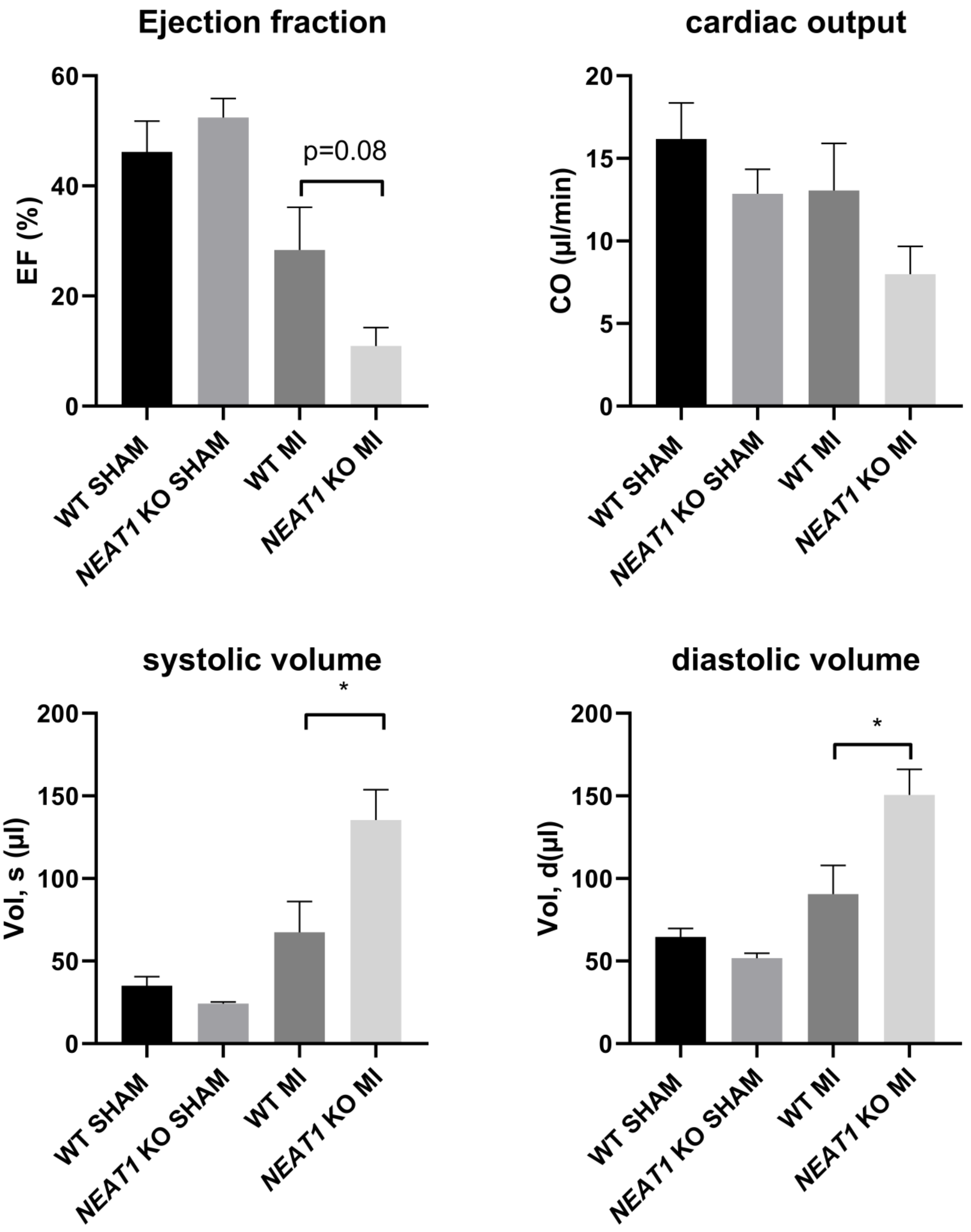

Figure 10: ***Neat1*** **deficiency leads to impaired heart function after myocardial infarction.**
Echocardiographic parameters of *Neat1* KO or wildtype (WT) mice were assessed 14 days after permanent LAD ligation or sham operation. EF=ejection fraction, CO=cardiac output. N=3-5 animals per group; Data are presented as mean ±SEM. * $p \leq 0.05$; Student´s t-test

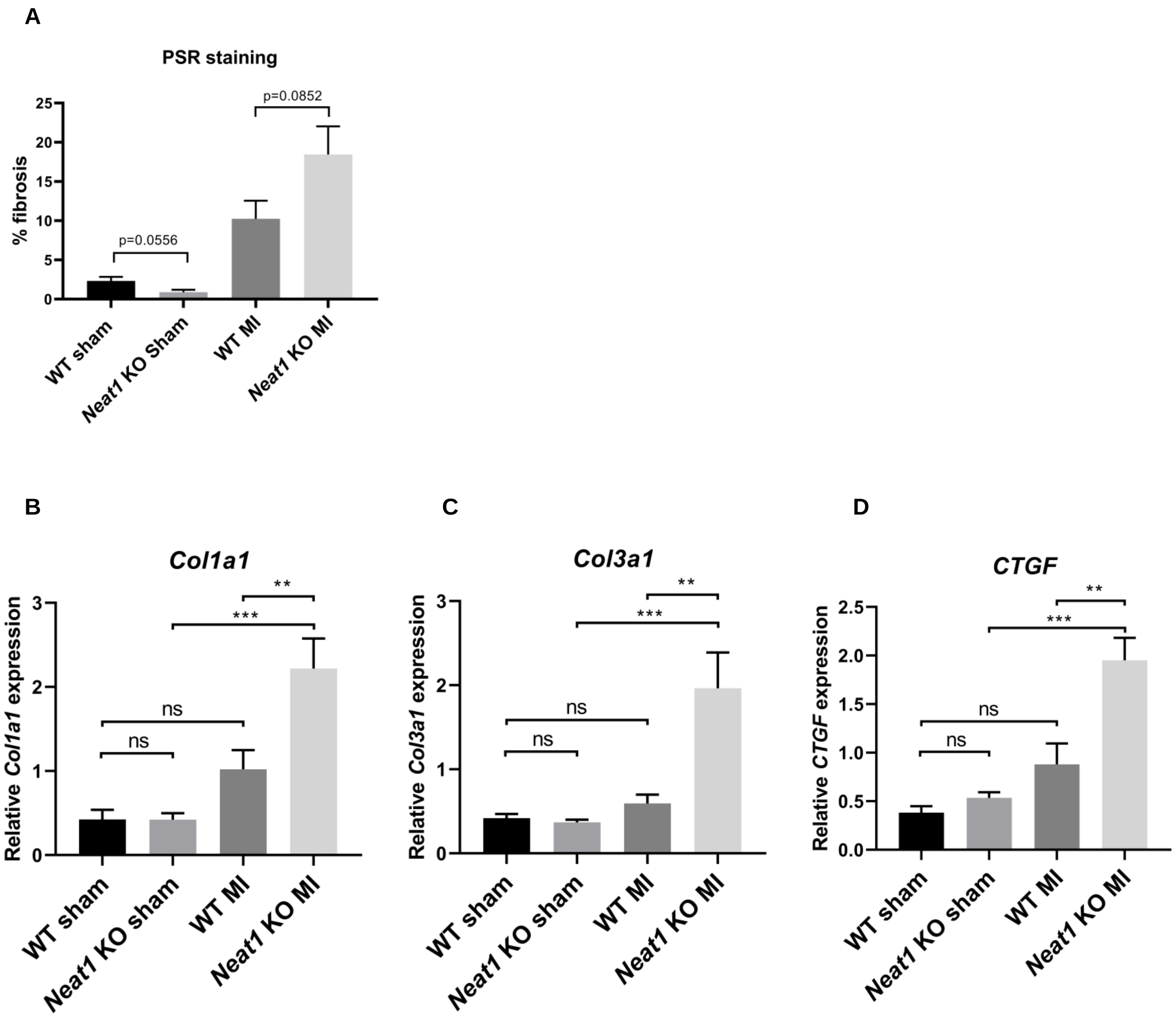

Figure 11: ***Neat1*** **knockout elevates fibrosis.**
(A) Picrosirius red staining (PSR) of left ventricles in *Neat1* knockout (KO) or wildtype (WT) mice 28 days after permanent left anterior descending artery (LAD) ligation (MI) or sham operation. **(B-D)** Murine hearts from *Neat1* KO or wildtype mice were removed 28 days LAD ligation and dissected into the remote and the peri-/infarct zone. Expression level of fibrotic markers *Col1a1*, *Col3a1* and *CTGF* was measured in the remote zone. of N=3-5 animals per group; Data are presented as mean ±SEM. ** $p \leq 0.01$;*** $p \leq 0.001$; ns=not significant. One-way ANOVA with Tukey´s multiple comparison test

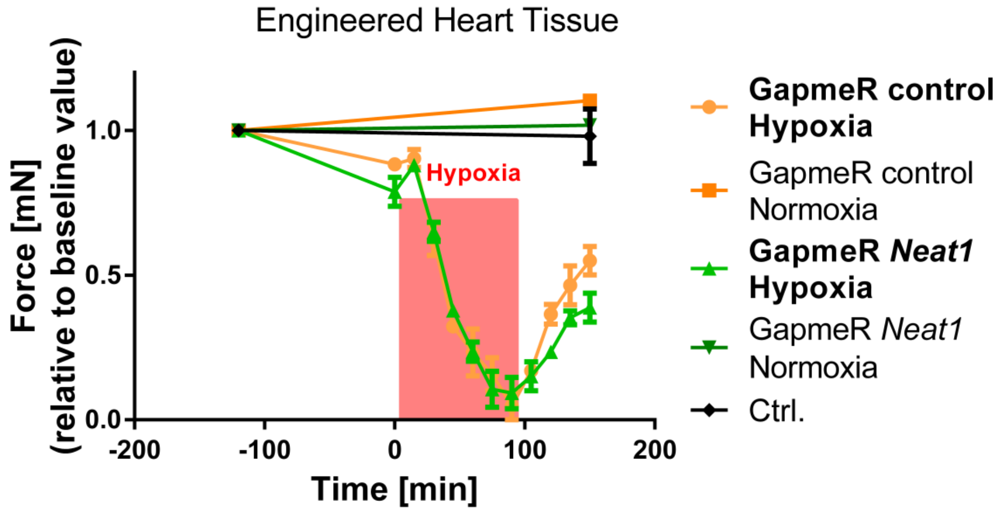

Figure 12: ***Neat1*** **influences force recovery in an hypoxia/reoxygenation** ***ex vivo*** **model**
Measurement of force in human engineered heart tissue under basal and hypoxic/reoxygenation conditions after treatment with GapmeR *Neat1* or GapmeR control. n=4

Supplements:

Methods:

Treatment of cells

To prevent HIF degradation, cells were treated with an inhibitor for the prolyl-hydroxylase which normally degrades the HIF protein. Therefore HL-1 cells were grown to a confluence of ~80 % and then incubated in medium supplemented with 1 mM DMOG (Dimethyloxalylglycine, Sigma) for 24h.

To deactivate P53 activity, cells were treated with Pifithrin-α (Enzo Life Sciences). Therefore cells were cultured in DMEM supplemented with 10 % FBS, 1 % P/S and 10 µM Pifithrin-α for 48h. As Pifithrin-α is dissolved in DMSO, the same volume of DMSO was used for the control group.

To induce P53 activity, cells were treated with 10 µM Nutlin-3 (Sigma Aldrich) for 48 h. As Nutlin-3 is dissolved in methanol, the same volume of methanol was used for the control group.

Preparation of EV production medium

To avoid the influence of exosomes present in the serum, FBS has to be pre-depleted of the contaminating exosomes. Therefore, FBS was ultracentrifuged over night for at least 10 h at 100,000xg and 4 °C.

Co-culture assay

For co-cultivation experiments, HL-1 cells were exposed to hypoxic (5 % CO_2, 0.2 % O_2 and 37 °C) or normoxic (5 % CO_2, 21 % O_2 and 37 °C) conditions for 24 h following 4 h reoxygenation and conditioned medium was collected. Next, 3T3 fibroblasts were incubated with HL-1 cardiomyocyte conditioned medium containing extracellular vesicles (in a ratio 1:3 fibroblast medium + HL-1 supernatant).

Scratch assay

NIH 3T3 cells were cultured in a 6-well plate and after 24 h transiently transfected with 50 nM GapmeR Neat1 or GapmeR control. On the next day, cells were trypsinized and 70 µl of cell suspension was transferred into each IBIDI insert (Thermo Fisher Scientific #80209). After additional 24 h, a horizontal scratch was performed, inserts were removed and images were immediately taken (=starting point t0). The migration of the cells was further tracked by taking images after 4-6 h. Afterwards, the area between the scratch was measured at each timepoint and the migration index was calculated according to: (area t0h-area t6h) / area t0.

Caspase3/7 assay

To assess the level of apoptosis, a Caspase 3/7 assay (Promega) was performed according to the manufacturer´s recommendations. Briefly, 48 h after the desired treatment, the same amount of Caspase-Glo 3/7 reagent as the culture medium was added to each well of a 96-well plate and carefully mixed by pipetting up and down several times. Afterwards, the plate was kept at room temperature in the dark for 1 h. Then, the supernatant (180 µl) was transferred to a white-walled 96-well plate and the developed luminescence was acquired in a luminometer (Synergy HT Reader, BioTek). Cells treated with 2 µM staurosporin for 3h served as a positive control.

Cell cycle assay

For analysis of the distribution of cells in cell cycle phases the Guava cell cycle flow cytometry based assay (Millipore) was used. Therefore, the cells were treated with the desired method, the medium was collected in a falcon and the cells harvested using the normal trypsinization protocol. Then, the cells were washed with PBS, transferred to the falcon containing the medium and centrifuged for 5 min at 300xg and 4 °C. After discarding the supernatant, the pellets were resuspended in 200 µl PBS and the suspension was transferred to a 96-well U-bottom plate before being centrifuged at 450xg for 5 min at RT with the brake on low. The supernatant was carefully removed and the remaining pellet was washed with 200 µl PBS and centrifuged as above. The supernatant was again carefully discarded leaving a small drop of PBS to resuspend the cells before adding dropwise 200 µl ice-cold 70 % ethanol to fixate the cells. Then, the plate was kept for at least 1 h at 4 °C. In order to stain the cells with propidium iodide (PI), which intercalates into nucleic acids and therefore gives a correlation with the DNA content of the cells, the cells were first again centrifuged at 450xg for 5 min at RT with the brake on low. After removing the supernatant the remaining pellet was resuspended in 200 µl PBS and incubated for 1 min at RT before being centrifuged as above. The supernatant was carefully discarded and 200 µl Guava Cell Reagent (containing PI to stain the cells) was added and mixed by pipetting up and down. The suspension was transferred to a new tube and incubated for 30 min at RT in the dark. Afterwards, the mixture was analyzed by flow cytometry measurements with Guava easyCyte (EMD Millipore).

RNA Isolation

The isolation of total RNA was performed using the TriFast (Peqlab) method according to manufacturer's instructions. Briefly, cells were washed with PBS and samples were homogenized directly from cell culture plates or flasks using 1 ml TriFast. After transferring to an Eppendorf tube, shaking vigorously and incubation for 5 min at RT for the complete dissociation of nucleoproteins, 200 µl chloroform was added to the samples. After inverting the tube and incubation for 3-10 min at RT, the different phases were separated by centrifugation at 12000 x g at RT for 5 min. 500 µl of the upper phase was transferred to a new tube and mixed with 500 µl Isopropanol and incubated for 10 min on ice to precipitate the RNA. A centrifugation step at 12000 x g for 10 min was followed, the supernatant was removed and the RNA pellet was washed twice with 1 mL of 75 % (v/v) ethanol by inverting the tube for 15 s. The RNA pellet was dried and dissolved in usually 20-30 µl RNase-free water and stored at -80 °C. The RNA-concentration was measured at 260 nm and 280 nm and the ratio was calculated using the Synergy HT multi-mode Reader (Biotek).

RNA Isolation of EVs

For the isolation of total RNA of EVs, supernatant, RNA microarray analysis and subcellular fractionation experiments the miRNeasy Mini Kit (Qiagen) was applied according to manufacturer's instructions. Briefly, samples were homogenized using 5 volumes of QIAzol Lysis reagent by vortexing. After an incubation time of 5 min, 5 µl of 1 fmol/µl spike-In-RNA (cel-miR-39 (Sigma-Aldrich)) was added followed by addition of 200 µl chloroform and 15 s of vortexing for immediate phase separation. The samples were incubated for 2–3 min and centrifuged for 15 min at 12,000xg at 4 °C. After transferring the upper aqueous phase (300 µl) into a new tube, 450 µl of 100 % ethanol was added and the samples were mixed by inverting the tubes. 700 µl of the sample were pipetted into an RNeasy Mini spin column in a 2 ml collection tube and centrifuged at 9600xg for 15 s at RT. Then the

flow-through was discarded and 700 µl Buffer RWT was added to the column, followed by 15 s of centrifugation at 9600xg at RT. Again the flow-through was discarded and the column was washed two times with 500 µl Buffer RPE and centrifuged at 9600xg for 15 s at RT. After placing the RNeasy Mini spin columns into a new 2 ml collection tube, the columns were again centrifuged at full speed for 2 min at RT to dry the spin column membrane and to ensure that no ethanol is carried over during RNA elution. The RNeasy Mini spin column was placed into a new 1.5 ml collection tube and 15 µl RNase-free water was added directly onto the RNeasy Mini spin column membrane and incubated for 5 min at RT and then centrifuged at 9600xg for 1 min at RT. To obtain a higher RNA concentration this step was repeated. The samples were stored at -80° C.

DNAse digestion

For detection of Neat1 or subcellular fractionation experiments total RNA was DNase treated with the on-column RNase-free DNase Set (Qiagen) prior to elution of RNA. After washing the column with RWT buffer, the flow-through was discarded and 10 µl DNase I stock solution (2.7 U/µl) was mixed with 70 µl RDD buffer and directly pipetted onto the column. After an incubation time of 15 min at RT 500 µl Buffer RWT (prepared with isopropanol) were added and the samples were centrifuged for 15 s at 9600xg. The flow-through was discarded and the protocol was continued according to section 4.2.2 with adding buffer RPE into the column.

Reverse transcription

For gene expression analysis, cDNA synthesis was performed using iScript select cDNA synthesis Kit (Bio-Rad) according to the manufacturer´s instructions. 5 µl of total RNA (100-1000 ng) was reverse transcribed using 2 µl Oligo (dT)20 or random primer, 4 µl iScript reaction mix and 1 µl iScript reverse transcriptase. The reaction mixture was filled up to 20 µl with dH20. For the cDNA synthesis using Oligo (dT)20 primers the protocol was the following: 42 °C for 90 min, followed by inactivation at 85 °C for 5 min. Reverse transcription using random primer reaction mix was performed at 25 °C for 5 min, 42 °C for 30 min and 85 °C for 5 min. The samples were stored at -20 °C.

Quantitative Real-Time PCR (qRT-PCR)

Quantification of the amount of specific gene expression was performed via real-time quantitative PCR (qPCR) using iQ SYBR Green Supermix (Bio-Rad). The qPCR was carried out in a 384-well plate using the ViiA™ 7 Real-Time PCR System (Thermo Fisher Scientific). The reaction mixture was the following: 5 µl iQ SYBR Green Supermix (Bio-Rad), 0.5 µl of a primer set (10 µM of each primer) or 1 µl of QuantiTect pimer assay (10x), 2 µl of cDNA (1:3 pre-diluted in nuclease-free water) and 2.5 µl or 2 µl nuclease-free water. The qPCR was run at 95 °C for 3 min to activate Taq polymerase, followed by 45 cycles of denaturation at 95 °C for 15 s, annealing at 60 °C or 58 °C for 30 s and elongation at 72° C for 40 s. After the amplification, a melting curve was generated by measuring fluorescence every 0.5 °C from 95 °C to 55 °C for 10 s to ensure a single PCR product. The qPCR data were analysed and gene expression was calculated using either the ΔΔct method or software-calculated starting quantities (SQ). These values were calculated based on the measurement of a standard curve generated from pooled cDNA samples (undiluted to 1:125 diluted). The relative gene expression was calculated by dividing each SQ mean value of a gene to the respective sample SQ mean value of a reference gene. As reference genes 18S ribosomal RNA (18S) or Gapdh were used.

Subcellular fractionation

In order to measure the subcellular distribution of lncRNAs and mRNAs in cytoplasmic, nuclear-soluble and chromatin-associated RNA fractions, fragmentation of HL-1 cardiomyocytes was performed as previously described 132. Therefore, 1 x 106 cells were harvested and centrifuged at 168xg at room temperature for 5 min. After discarding the supernatant, the resulting cell pellet was lysed in 175 µl cold sample buffer (50 mM Tris-HCl pH 8, 140 mM NaCl, 1.5 mM MgCl2, 0.5% Igepal NP-40, 2 mM Vanadyl Ribonucleoside complex) and incubated for 5 min. on ice. Then, the cell suspension was centrifuged at 300xg at 4 °C and the resulting supernatant (cytoplasmic fraction) was transferred into a new tube and stored on ice. The nuclear pellet was resuspended in 175 µl cold RLN2 solution (50 mM Tris-HCl pH 8, 500 mM NaCl, 1.5 mM MgCl2, 0.5% NP-40, 2 mM Vanadyl Ribonucleoside complex) and incubated for 5 min. on ice. After centrifugation for 2 min. at 16,360xg at 4 °C, the resulting pellet (chromatin-associated fraction) and supernatant (nuclear-soluble fraction) were stored on ice. Afterwards, 1 ml of Qiazol was added to all the fractions and RNA isolation was performed using the miRNeasy Mini Kit (Qiagen) including DNA digestion steps.

LncRNA profiling

For microarray analysis, total RNA was isolated from cardiomyocyte-derived microvesicles and exosomes as well as HL-1 cells using the miRNeasy Mini Kit (n=3) and the RNA quality was assessed using the Agilent Bioanalyzer 2100. These samples were globally profiled using the Arraystar Mouse LncRNA Microarray v 3.0 (Arraystar Inc, Rockville, MD) that allows a simultaneous detection of 35,923 lncRNAs and 24,881 coding transcripts. The microarray was performed according to the Agilent One-Color Microarray-Based Gene Expression Analysis protocol (Agilent Technology). Briefly, ribosomal RNA was separated from mRNA using the mRNA-ONLY™ Eukaryotic mRNA Isolation Kit (Epicentre) before amplifying and transcribing each sample into fluorescent cRNA along the entire length of the transcripts without 3' bias utilizing a random priming method (Arraystar Flash RNA Labeling Kit, Arraystar). Then, the labelled cRNAs were hybridized onto the Mouse LncRNA Array v3.0 (8 x 60K, Arraystar), the slides were washed and the arrays were scanned by the Agilent Scanner G2505C. Agilent Feature Extraction software (version 11.0.1.1) was used to analyze the acquired array images.

After quantile-normalization of the raw data with the GeneSpring GX v12.1 software package (Agilent Technologies), lncRNAs that at least have 6 out of 18 samples flags in Present or Marginal ("All Targets Value") were chosen for further data analysis. Dysregulated lncRNAs were identified through Volcano Plot filtering ($p \leq 0.05$, $FC \leq 0.5$ or ≥ 2).

RNA Seq

Library generation, quality control, and quantification:

500 ng of total RNA per sample were utilized as input for mRNA enrichment procedure with 'NEBNext® Poly(A) mRNA Magnetic Isolation Module' (E7490L; New England Biolabs) followed by stranded cDNA library generation using 'NEBNext® Ultra II Directional RNA Library Prep Kit for Illumina' (E7760L; New England Biolabs). All steps were performed as recommended in user manualE7760 (Version 1.0_02-2017; NEB) except that all reactions were downscaled to 2/3 of initial volumes. Furthermore, one additional purification step was introduced at the end of the standard

procedure, using 1x 'Agencourt® AMPure® XP Beads' (#A63881; Beckman Coulter, Inc.) and fragmentation time was set to 12 minutes.

cDNA libraries were barcoded by single indexing approach, using 'NEBNext Multiplex Oligos for Illumina – Set 1 and 2' (Index Primer 1-12, 18-23). All generated cDNA libraries were amplified with 6 cycles of final pcr.

Fragment length distribution of individual libraries was monitored using 'Bioanalyzer High Sensitivity DNA Assay' (5067-4626; Agilent Technologies). Quantification of libraries was performed by use of the 'Qubit® dsDNA HS Assay Kit' (Q32854; ThermoFisher Scientific).

Library denaturation and Sequencing run:

Equal molar amounts of eighteen individually barcoded libraries were pooled. Accordingly, each analyzed library constitutes 5.6% of overall flowcell capacity. The library pool was denatured with NaOH and was finally diluted to 1.5pM according to the Denature and Dilute Libraries Guide (Document # 15048776 v02; Illumina). 1.3 ml of denatured pool was loaded on an Illumina NextSeq 550 sequencer using a High Output Flowcell for paired-end reads (20024907; Illumina). Sequencing was performed with the following settings: Sequence reads 1 and 2: 81bp each; Index read 1: 6bp.

BCL to FASTQ conversion:

BCL files were converted to FASTQ files using bcl2fastq Conversion Software version v2.20.0.422 (Illumina).

Raw data processing and quality control:

Raw data processing was conducted by use of nfcore/rnaseq (version 1.5dev) which is a bioinformatics best-practice analysis pipeline used for RNA sequencing data at the National Genomics Infrastructure at SciLifeLab Stockholm, Sweden. The pipeline uses Nextflow, a bioinformatics workflow tool. It pre-processes raw data from FastQ inputs, aligns the reads and performs extensive quality-control on the results. The genome reference and annotation data were taken from GENCODE.org (Mus musculus; GRCm38.p6; release M17).

Normalization and differential expression analysis:

Normalization and differential expression analysis was performed with DESeq2 (Galaxy Tool Version 2.11.40.2) with default settings except for "Output normalized counts table" which was set to "Yes".

Gene enrichment analysis

Ensembl gene ID list of the 745 significant genes (padj<0.05) were submitted to DAVID functional annotation tool (1) for gene set enrichment analysis. GOTERM biological process were used as annotation resource.

RNA FISH

In order to detect and localize lncRNAs within the cell, RNA fluorescent in situ hybridization was performed. Therefore, cells were seeded on 18 mm round coverglass slips in a 12-well plate and cultured for 24 h before be exposed to normoxic (21 % O2) or hypoxic (0.1 % O2) condtions for additional 24 h. For fixation, the cells were washed with PBS and 1 ml fixation buffer (3.7 % (v/v) formaldehyde in 1xPBS) was added. After incubation for 10 min at RT, the cells were washed twice with 1xPBS and permeabilized in 1 ml 70 % ethanol for 1 h at 4 °C. The ethanol was removed and 1 ml wash buffer A (10% formamide in Stellaris RNA FISH Wash Buffer A (Biosearch Technologies)) was added. After incubation for 5 min at RT, a 100 µl drop of hybridization buffer (10 % formamide in Stellaris RNA FISH Hybridization Buffer (Biosearch Technologies)) containing the probe (125 nM) was added onto a parafilm. The coverslip was transferred to the drop with cells facing down an incubated in the dark for 16 h at 37 °C in a humidified chamber. On the next day, the coverglass slips were transferred to a new 12-well plate containing 1 ml wash buffer A and incubated in the dark at 37 °C for 30 min. For staining the nuclei, the wash buffer was aspirated and 1 ml DAPI solution (wash Buffer A consisting of 5 ng/ml DAPI) was pipetted in each well and incubated for additional 30 min in the dark at 37 °C. After removing the staining solution, 1 ml wash buffer B (Biosearch Technologies) was added and incubated for 5 min at RT. Afterwards, the coverslips were mounted in Vectashield mounting medium and images were taken.

Electron microscopy

Purified cardiomyocyte-derived vesicles were used for electron microscopic analysis as described previously 61. In brief, EVs were isolated using the above mentioned protocol and resuspended in 50 µl fixation solution (0.1 % glutaraldehyde, 4 % PFA in 0.2 mol HEPES buffer). Then, the fixed EVs were loaded onto formvar carbon-coated grids and dried for 20 min at 40 °C. The loaded grids were again fixed in 1 % glutaraldehyde (Agar Scientific Limited) in HEPES buffer for 5 min and subsequently transferred to a drop of 100 µl distilled water several times. After contrasting the grids in 4 % uranyl-acetate solution for 5 min at RT, the grids were finally embedded in a mixture of uranyl acetate (4 %) and methyl cellulose (2 %) on ice for 5 min. For removing excess fluid, the grids were placed on filter paper and dried for 10 min before they were stored in a grid storage box. Ultra-thin sections of cardiomyocyte-derived vesicles were analyzed with an electron microscope (Morgagni 268, FEI) and micrographs of representative areas were taken by a digital camera (Veleta TEM camera, Olympus Europa Holding GMBH).

Fluorescence confocal microscopy

To investigate a potential vesicle uptake into fibroblasts, immunofluorescence confocal microscopy measurements were performed. Therefore, cover slips were placed into wells of a 24-well plate and fibroblasts were seeded accordingly. On the next day, labelled vesicles were incubated with fibroblasts for 30 min, 2 h and 20 h at 37 °C or 20 h at 4 °C. After washing the cells carefully with PBS, the cells were fixed in 4% paraformaldehyde (PFA) for 15 min at RT. Then, the cells were washed again twice with PBS and permeabilized with 0.1 % Triton X-100 for 10 min at RT following two washing steps. In the next step, the cells were stained with Dapi (1:1000, Sigma) and TRITC-Phalloidin (1:100, Sigma) in 5 % Donkey serum in PBS for 30 min at RT in the dark. After washing with PBS, slides

were transferred to a microscope slide and embedded with Prolong Gold Antifade reagent (life technologies). Confocal imaging was performed with a Zeiss LSM 780 microscope using a Plan-Apochromat 63x/1.40 Oil immersion objective. In all cases z-stacks were taken covering the entire cell volume.

Cardiac cell fractionation

To assess the cardiac cell-specific gene expression of Neat1, cardiac cells were isolated from C57BL/6N mice. Therefore, mice were anaesthetized via inhalation of 3 % isoflurane in a chamber and injected with 100 µl heparin (500 I.E./ml, i.p.). After cutting the skin between two tracheal trabeculae, the thorax was opened and the heart was cannulated through the aorta. The heart was then excised and immediately retrograde perfused with pre-warmed perfusion buffer for 6 min (113 mM NaCl, 4.7 mM KCl, 0.6 mM KH2PO4, 0.6 mM Na2HPO4, 1.2 mM MgSO4-7H2O, 0.032 mM Phenol Red, 12 mM NaHCO3, 10 mM KHCO3, 10 mM HEPES, 30 mM Taurine, 0.1 % Glucose, 10 mM 2,3-Butanedione monoxime) followed by pre-warmed digestion buffer (113 mM NaCl, 4,7 mM KCl, 0.6 mM KH2PO4, 0.6 mM Na2HPO4, 1.2 mM MgSO4-7H2O, 0.032 mM Phenol Red, 12 mM NaHCO3, 10 mM KHCO3, 10 mM HEPES, 30 mM Taurine, 0.1 % Glucose, 10 mM 2,3-Butanedione monoxime, 12.5 µM CaCl2, 700 U/ml Collagenase II) for 20 minutes. After removing the atria, cardiac ventricles were further dissociated mechanically by cutting into small pieces using digestion buffer and shearing through a 1 ml syringe. Collagenase II digestion was stopped by adding stop buffer (perfusion buffer supplemented with 10 % FBS and 12.5 µM CaCl2). The obtained cell suspension was filtered through a 100 µm cell strainer and mixed with AMCF medium containing 1 % FBS (10.8 g MEM, 10 % NaHCO3, 2ng/ml vitamin B12, 100 U/ml penicillin/100 µg streptomycin). To separate cardiomyocytes and non-myocytes, the cell suspension was sedimented for 10 min on ice. CMC pellets were washed in PBS, centrifuged for 5 min at 3000 rpm at 4 °C, frozen in liquid nitrogen and stored at -80 °C. The remaining supernatants containing the non-myocyte fraction were centrifuged at 1500 rpm for 5 min at RT and the resulting cell pellets dissolved in plating medium containing 1 % FBS and pre-plated for 1 h on a 10 cm petri dish in a 1 % CO2 incubator. The attached cells were washed with PBS and harvested with a cell scraper. After centrifugation at 3000 rpm for 5 min at 4 °C, the pellets were frozen in liquid nitrogen and stored at -80 °C. The remaining pre-plating medium, containing non-attached endothelial cells, was then centrifuged at 1500 rpm for 5 min at 4 °C. Afterwards, the resulting pellets were resuspended in MACS buffer (MACS bovine serum albumin stock solution diluted 1:20 in auto-MACS rinsing solution, both from Miltenyi Biotec) and mixed with CD146 MACS beads (Miltenyi Biotec) and incubated for 15 min at 4 °C. Then, additionally MACS buffer was added. After a centrifugation step at 1500 rpm for 5 min at 4 °C, the resulting pellets were suspended in MACS buffer and applied on a MACS separating column. The columns were washed 3 times with MACS buffer and then removed from the magnetic field. The resulting ECs were collected in MACS buffer and centrifuged at 3000 rpm for 5 min at 4 °C. The resulting pellets were frozen in liquid nitrogen and stored at -80 °C.

In vivo vesicle isolation

Cardiac EVs were isolated from the left ventricule of C57Bl6 mice with sham or infarcted hearts by our cooperation partners in Paris (Silvestre, Loyer, Boulanger, Cardiovascular research centre, INSERM) as published recently (2). Those vesicles were isolated between 0 and 14 days after

coronary artery ligature according to the method previously established for ischemic skeletal muscle 133. Briefly, murine hearts were perfused with saline solution, excised and rinsed. The left ventricle was isolated and minced mechanically for 30 s. Homogenates were centrifuged to remove tissue, before pelleting MVs and Exo via sequential centrifugation and filtration steps. The different EV subtypes were further characterized using NTA and the cellular origin of EVs was determined by high sensitive flow cytometry.

References

1. Huang DW, Sherman BT, Lempicki RA. Systematic and integrative analysis of large gene lists using DAVID bioinformatics resources. Nature protocols 2009;4(1):44–57.

2. Loyer X, Zlatanova I, Devue C, et al. Intra-Cardiac Release of Extracellular Vesicles Shapes Inflammation Following Myocardial Infarction. Circulation research 2018;123(1):100–6.
