# Supplementary material for: Long Noncoding RNA-Enriched Vesicles Secreted by Hypoxic Cardiomyocytes Drive Cardiac Fibrosis
Source: Mol Ther Nucleic Acids. 2019 Sep 13;18:363–74. doi: 10.1016/j.omtn.2019.09.003 (PMC6807307; doi:10.1016/j.omtn.2019.09.003)
Supplement: Document S2. Article plus Supplemental Information [file mmc3.pdf]

Molecular Therapy
Nucleic Acids
Original Article

Long Noncoding RNA-Enriched Vesicles Secreted by Hypoxic Cardiomyocytes Drive Cardiac Fibrosis

Franziska Kenneweg,[1] Claudia Bang,[1] Ke Xiao,[1] Chantal M. Boulanger,[2] Xavier Loyer,[2] Stephane Mazlan,[2] Blanche Schroen,[3] Steffie Hermans-Beijnsberger,[3] Ariana Foinquinos,[1] Marc N. Hirt,[4,5] Thomas Eschenhagen,[4,5] Sandra Funcke,[4,5,6] Stevan Stojanovic,[1] Celina Genschel,[1] Katharina Schimmel,[1] Annette Just,[1] Angelika Pfanne,[1] Kristian Scherf,[1] Susann Dehmel,[7] Stella M. Raemon-Buettner,[7] Jan Fiedler,[1,9] and Thomas Thum[1,8,9]

[1]Institute of Molecular and Translational Therapeutic Strategies (IMTTS), Hannover Medical School, Hannover, Germany; [2]INSERM UMR-970, Paris Cardiovascular Research Center, Université Paris Descartes Hôpital Européen Georges, Paris, France; [3]Department of Cardiology, CARIM School for Cardiovascular Diseases, Maastricht University, Maastricht, the Netherlands; [4]Institute of Experimental Pharmacology and Toxicology, University Medical Center Hamburg-Eppendorf, Hamburg, Germany; [5]DZHK (German Centre for Cardiovascular Research), partner site Hamburg/Kiel/Lübeck, Germany; [6]Department of Anesthesiology, Center of Anesthesiology and Intensive Care Medicine, University Medical Center Hamburg-Eppendorf, Hamburg, Germany; [7]Fraunhofer Institute for Toxicology and Experimental Medicine (ITEM), Hannover, Hannover, Germany; [8]REBIRTH Excellence Cluster, Hannover Medical School, Hannover, Germany

Long non-coding RNAs (lncRNAs) have potential as novel therapeutic targets in cardiovascular diseases, but detailed information about the intercellular lncRNA shuttling mechanisms in the heart is lacking. Here, we report an important novel crosstalk between cardiomyocytes and fibroblasts mediated by the transfer of lncRNA-enriched extracellular vesicles (EVs) in the context of cardiac ischemia. lncRNA profiling identified two hypoxia-sensitive lncRNAs: *ENSMUST00000122745* was predominantly found in small EVs, whereas lncRNA *Neat1* was enriched in large EVs *in vitro* and *in vivo*. Vesicles were taken up by fibroblasts, triggering expression of profibrotic genes. In addition, lncRNA *Neat1* was transcriptionally regulated by P53 under basal conditions and by HIF2A during hypoxia. The function of *Neat1* was further elucidated *in vitro* and *in vivo*. Silencing of *Neat1 in vitro* revealed that *Neat1* was indispensable for fibroblast and cardiomyocyte survival and affected fibroblast functions (reduced migration capacity, stalled cell cycle, and decreased expression of fibrotic genes). Of translational importance, genetic loss of *Neat1 in vivo* resulted in an impaired heart function after myocardial infarction highlighting its translational relevance.

INTRODUCTION

Cardiovascular diseases (CVDs), including myocardial infarction (MI), lead to an adverse remodeling process in the heart. This is typically initiated by different stress factors such as cardiac ischemia as well as pressure and/or volume overload, resulting in maladaptive responses to maintain the cardiac function and, ultimately, contributing to heart failure.[1–3] There is intercellular crosstalk between main cell types of the heart such as cardiac fibroblasts, cardiomyocytes, and endothelial cells to coordinate the initiation and progression of cardiac remodeling.[4,5] Since the discovery that non-coding RNAs (ncRNAs) such as microRNAs and long non-coding RNAs (lncRNAs) are also present in human body fluids, partly via the inclusion into extracellular vesicles (EVs),[6,7] they have emerged as paracrine effectors by which cardiac cell types can communicate with each other and respond to stress conditions and are considered to serve as novel clinical biomarkers. In the context of ncRNAs/vesicle-mediated communication mechanisms in the heart, previous studies identified that cardiac fibroblasts are able to release exosomes enriched in microRNAs that are taken up by cardiomyocytes, contributing to the development of cardiomyocyte hypertrophy.[8] However, whether lncRNA-enriched EVs may also serve as cell-cell communicators during cardiac ischemia is not well known so far. In this study, we characterized a novel intercellular communication route between hypoxic cardiomyocytes and fibroblasts via the transfer of lncRNA-enriched EVs and studied their potential biological function in ischemic heart disease including *in vitro*, *in vivo*, and human cell/tissue studies.

RESULTS

Ischemia Drives Extracellular Vesicle Secretion by Cardiomyocytes

Ischemia is well known to drive cardiac remodeling after MI. To understand a potential crosstalk between cardiomyocytes and

Received 20 June 2019; accepted 6 September 2019;
https://doi.org/10.1016/j.omtn.2019.09.003.

[9]These authors contributed equally to this work.

Correspondence: Thomas Thum, MD, PhD, Institute of Molecular and Translational Therapeutic Strategies (IMTTS), Hannover Medical School, OE8886, Carl-Neuberg-Straße 1, 30625 Hannover, Germany.
E-mail: thum.thomas@mh-hannover.de

This is an open access article under the CC BY license (http://creativecommons.org/licenses/by/4.0/).

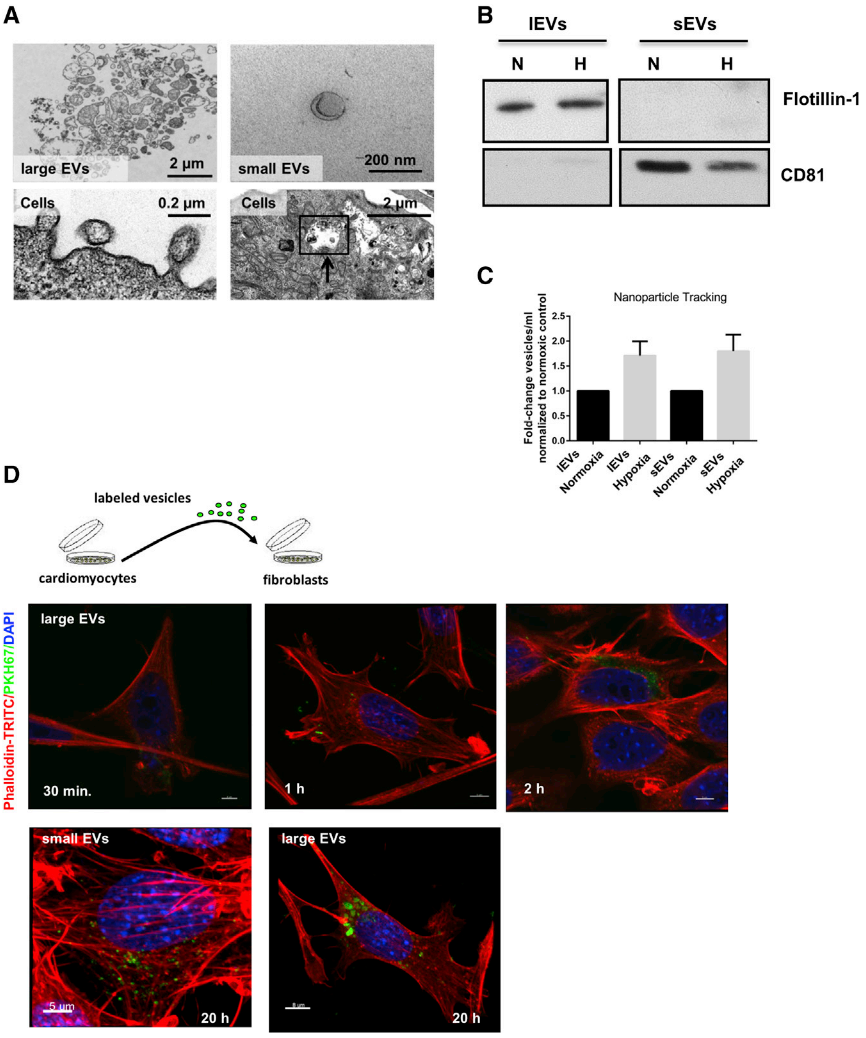

Figure 1. Cardiomyocytes Produce and Secrete EVs

(A) Electron microscopy images of cardiomyocytes and purified cardiomyocyte-derived vesicles. Cytoplasm of cardiomyocytes with MVBs. The membrane of the MVB invaginated inward (black arrow), resulting in the formation of intraluminal vesicles. Outward budding of the plasma membrane led to the release of lEVs. The sEVs showed a cup-shaped structure with an approximate size of 100 nm, whereas the morphology of lEVs was rather diverse. (B) Western blot of isolated EVs derived from cardiomyocytes that were exposed to hypoxic (H) conditions for 24 h following 4 h reoxygenation or normoxic (N) conditions for 28 h. (C) Measurement of particle concentration using nanoparticle tracking analysis. n = 3 independent experiments, one-column t test. (D) EV uptake experiment. Isolated cardiomyocyte-derived lEVs were labeled with a green fluorescent dye (PKH67); co-cultured with fibroblasts for 30 min, 1 h, and 2 h at 37°C; and confocal images were taken. Fibroblasts were stained with DAPI (blue) and phalloidin-TRITC (red). After 20 h, sEVs and lEVs were internalized and located in the cytoplasm of fibroblasts. Scale bars, 5 µm. n = 3 independent experiments. Cardiomyocytes = cells.

fibroblasts via EVs and to mimic the clinical situation of ischemia/reperfusion *in vitro*, cardiomyocytes were exposed to hypoxic conditions followed by reoxygenation, and EVs—large EVs (lEVs) and small EVs (sEVs)—were isolated (nomenclature was according to Théry et al.[9]). By electron microscopy, we observed the formation of lEVs originating through the outward budding of the plasma membrane and the release of sEVs into the extracellular fluid (Figure 1A). We identified the appearance of typical multivesicular bodies (MVBs) enriched with intraluminal vesicles inside the cytoplasm, which were derived through the inward invagination of the MVB membrane (Figure 1A, black arrow). Further characterization of isolated EVs from the conditioned medium of cardiomyocytes demonstrated that sEVs were rounded in shape, with an approximate size of 100 nm in diameter, as shown by electron microscopy (Figure 1A) and expressed the marker protein CD81 (Figure 1B). In contrast, the lEV fraction displayed a multifaceted morphology and the presence of the characteristic marker Flotillin-1 (Figure 1B). Moreover, nanoparticle tracking analysis validated that hypoxia stimulated secretion of EVs (Figure 1C).

Hypoxic Cardiomyocyte-Derived Vesicles Are Transferred to Fibroblasts, Leading to a Profibrotic Phenotype

Next, we investigated whether cardiomyocyte-derived vesicles would be transferred to and finally incorporated by fibroblasts. Secreted EVs—both small and large vesicles—were isolated, and subsequently, fluorescently labeled vesicles were incubated with fibroblasts. Uptake of vesicles into fibroblasts was analyzed by confocal imaging, revealing that both vesicle subtypes are internalized and located in the cytoplasm of fibroblasts in a time-dependent manner (Figure 1D; Figure S1). In addition, 3D reconstructions of the confocal image *Z* stacks confirmed that vesicles are taken up into fibroblasts and are not only attached to the cell surface (Video S1).

Next to confocal imaging, a potential vesicle-mediated crosstalk between hypoxic cardiomyocytes and fibroblasts was further studied. The conditioned medium of cardiomyocytes was collected and incubated with fibroblasts (Figure 2A). We observed increased expression levels of the profibrotic marker genes *Col1a1*, *Col3a1*, *Ctgf*, and *MMP2* when fibroblasts were cultured with hypoxic conditioned medium compared to normoxic conditioned medium (Figures 2B–2E). Stepwise depletion of vesicles out of the medium abolished the profibrotic response in fibroblasts (Figures 2F–2I), indicating that cardiomyocyte-derived vesicles are a major factor in the fibrotic response.

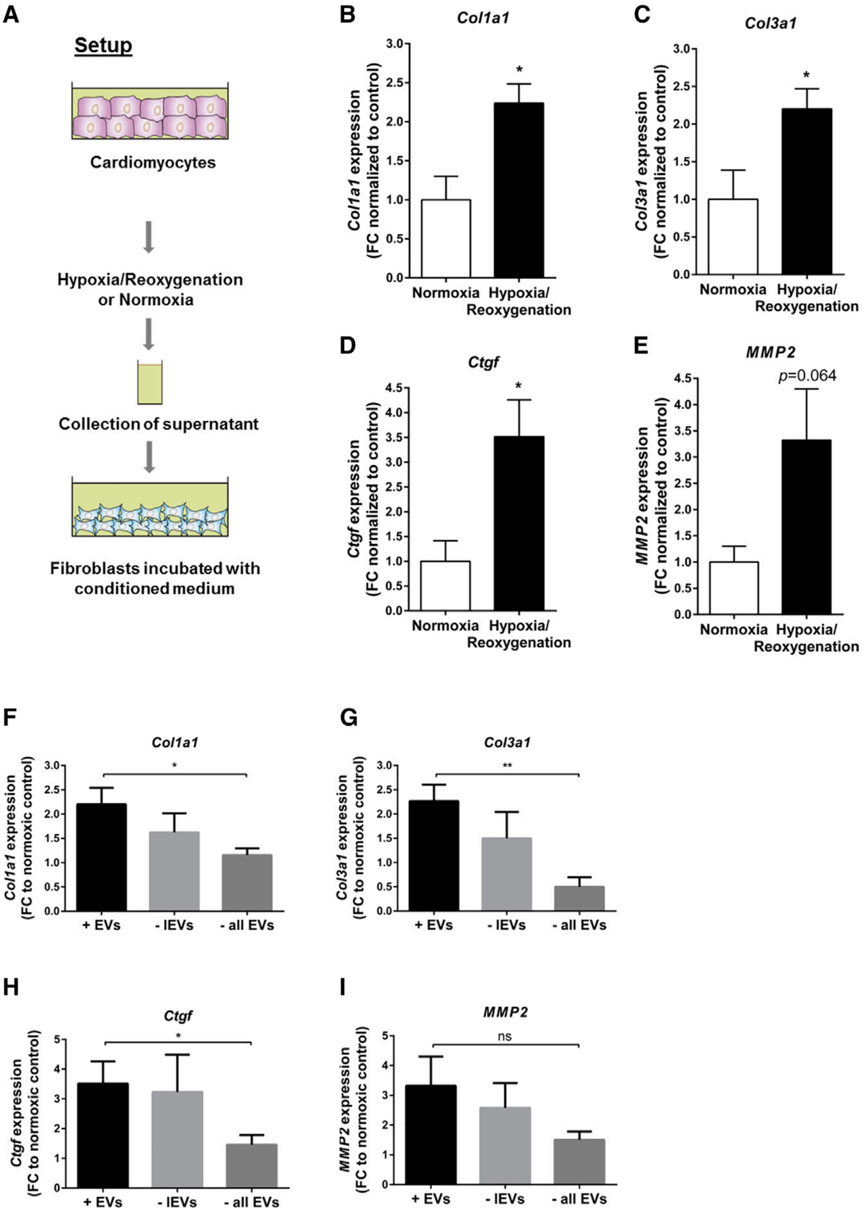

Figure 2. Cardiomyocyte-Derived Vesicles Are Taken Up into Fibroblasts, Leading to Profibrotic Transcriptome Changes

(A) Setup to evaluate a potential vesicle-mediated cross-talk between hypoxic cardiomyocytes and fibroblasts. (B–E) Expression levels of fibrosis-related genes *Col1a1* (B), *Col3a1* (C), *Ctgf* (D), and *MMP2* (E) in fibroblasts after culture with hypoxic or normoxic conditioned medium. (F–I) Stepwise depletion of large vesicles (– lEVs) or all extracellular vesicles (–all EVs) out of the conditioned medium abolished the profibrotic phenotype: (F) *Col1a1*; (G) *Col3a1*; (H) *Ctgf*; and (I) *MMP2*. Data are presented as mean ± SEM. n = 4–5 independent experiments. *$p \leq 0.05$; **$p \leq 0.01$; ns, not significant; Student's t test. FC, fold change to normoxic control.

In Response to Hypoxia/Reoxygenation, lncRNAs Are Enriched in Cardiomyocyte-Derived Small and Large Vesicles

Non-coding RNAs have been shown to be crucial for the crosstalk between fibroblasts and cardiomyocytes.[10] Here, we investigated changes of the vesicle-based lncRNA transcriptome during normoxia and hypoxia/reoxygenation in cardiomyocytes, as well as their secreted lEVs and sEVs. lncRNA profiling revealed that in both vesicle subtypes and cardiomyocytes, a large amount of lncRNAs were differentially expressed during hypoxia/reoxygenation (Figure 3A). Interestingly, when comparing regulated lncRNAs, only a small amount of lncRNAs—28 upregulated (indicated in red) and 12 downregulated (indicated in green)—showed a shared overlap between cardiomyocytes and both vesicle subtypes (Figures 3B and 3C). Suprisingly, only 135 downregulated or 239 upregulated lncRNAs are deregulated in both sEVs and lEVs, indicating a selective packaging and sorting mechanism of lncRNAs into specific vesicle subtypes. After *in silico* filtering according to stringent selection criteria, such as focus on upregulated lncRNAs which have intergenic localization and human conservation to translate findings in the future to patient data (Figure 3D), only lncRNAs with an abundancy over a certain threshold were considered for further analysis. We identified 7 deregulated lncRNA candidates in large vesicles as well as 7 potential candidates in the sEV fraction (Figures 3E and 3F). Subsequent studies were focused on two lncRNA candidates (Ensembl: *ENSMUST00000122745* and *Neat1*), since these lncRNAs exhibited the highest abundance in cardiac vesicles, suggesting a possible paracrine function.

After validation via qPCR, we measured the gene expression of these two selected candidates in hypoxic cardiomyocytes. We identified both lncRNAs to be hypoxia responsive. In particular, qRT-PCR experiments revealed increased expression levels of *ENSMUST00000122745* after 12 h of hypoxia in cardiomyocytes, whereas this effect was attenuated following reoxygenation (Figure 4A). In contrast, the expression level of *ENSMUST00000122745* was significantly increased in cardiomyocyte-derived sEVs after hypoxia/reoxygenation (Figure 4B), indicating that this lncRNA might be transported out of the cell via vesicle transfer, leading to reduced intracellular levels. Subcellular fractionation of cardiomyocytes showed that *ENSMUST00000122745* is not exclusively expressed in one specific compartment (Figure S2A), indicating various functional roles at different target sites. In addition, even though *ENSMUST00000122745* is expressed in the heart, it is also present in other organs, supporting this hypothesis (Figure S2B). *In vivo*,

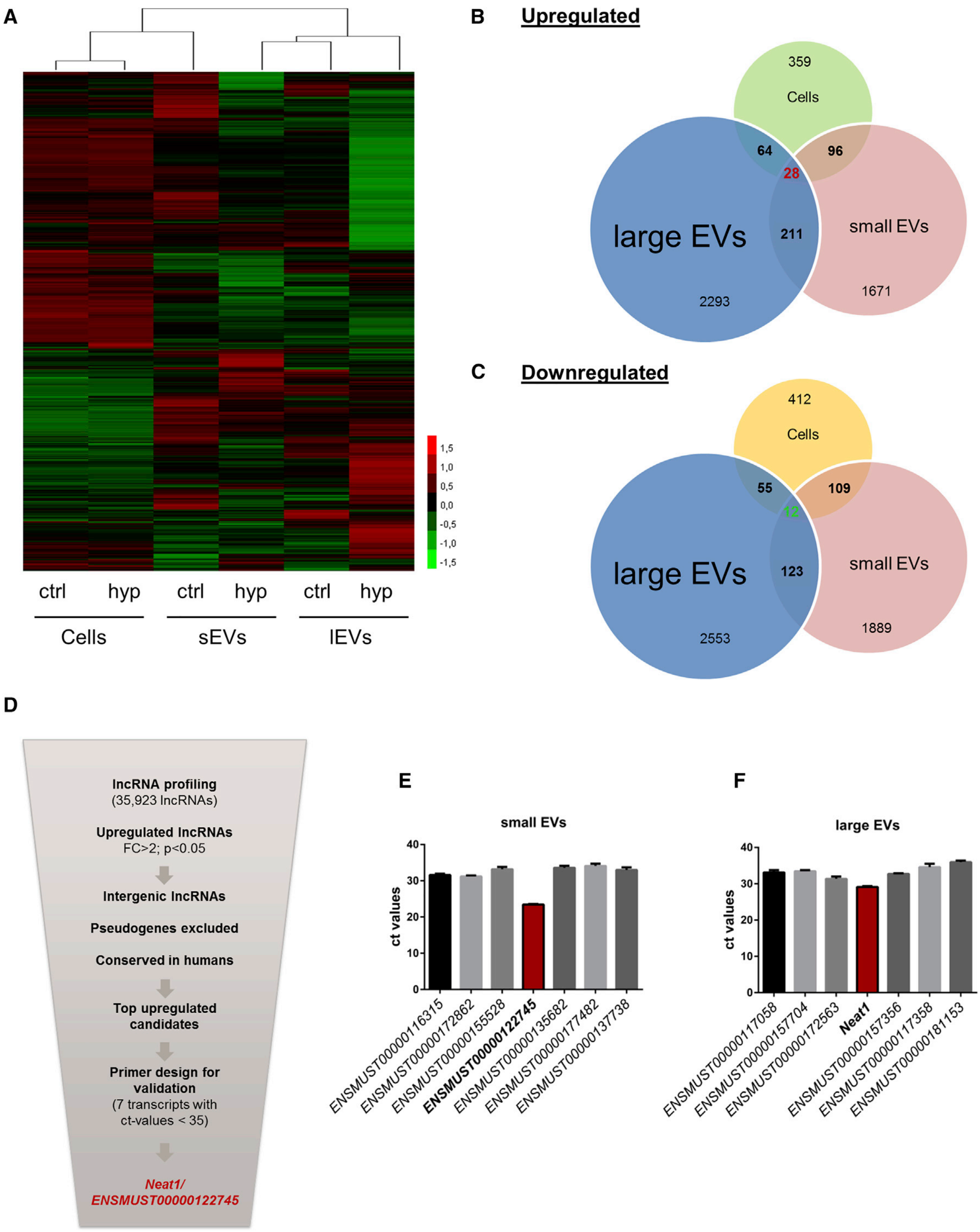

Figure 3. Deregulated lncRNAs in Extracellular Vesicles and Cardiomyocytes during Hypoxia/Reoxygenation

(A) Heatmap of lncRNA mouse array in cardiomyocytes, sEVs, and lEVs exposed to hypoxic conditions for 24 h following 4 h of reoxygenation or normoxic conditions for 28 h. n = 3 independent experiments. (B and C) Different lncRNAs are (B) upregulated and (C) downregulated during hypoxia/reoxygenation in lEVs, sEVs, and cells, and only 12 (green, downregulated) or 28 (red, upregulated) are shared by all. (D–F) Scheme of the selection strategy (D) to identify lncRNA candidates in lEVs and sEVs leading to 2 candidates: Ensembl: *ENSMUST00000122745* in sEVs (E) and *Neat1* in lEVs (F). n = 3–6 independent experiments. Cells = cardiomyocytes; ctrl = normoxia; hyp = hypoxia/reoxygenation.

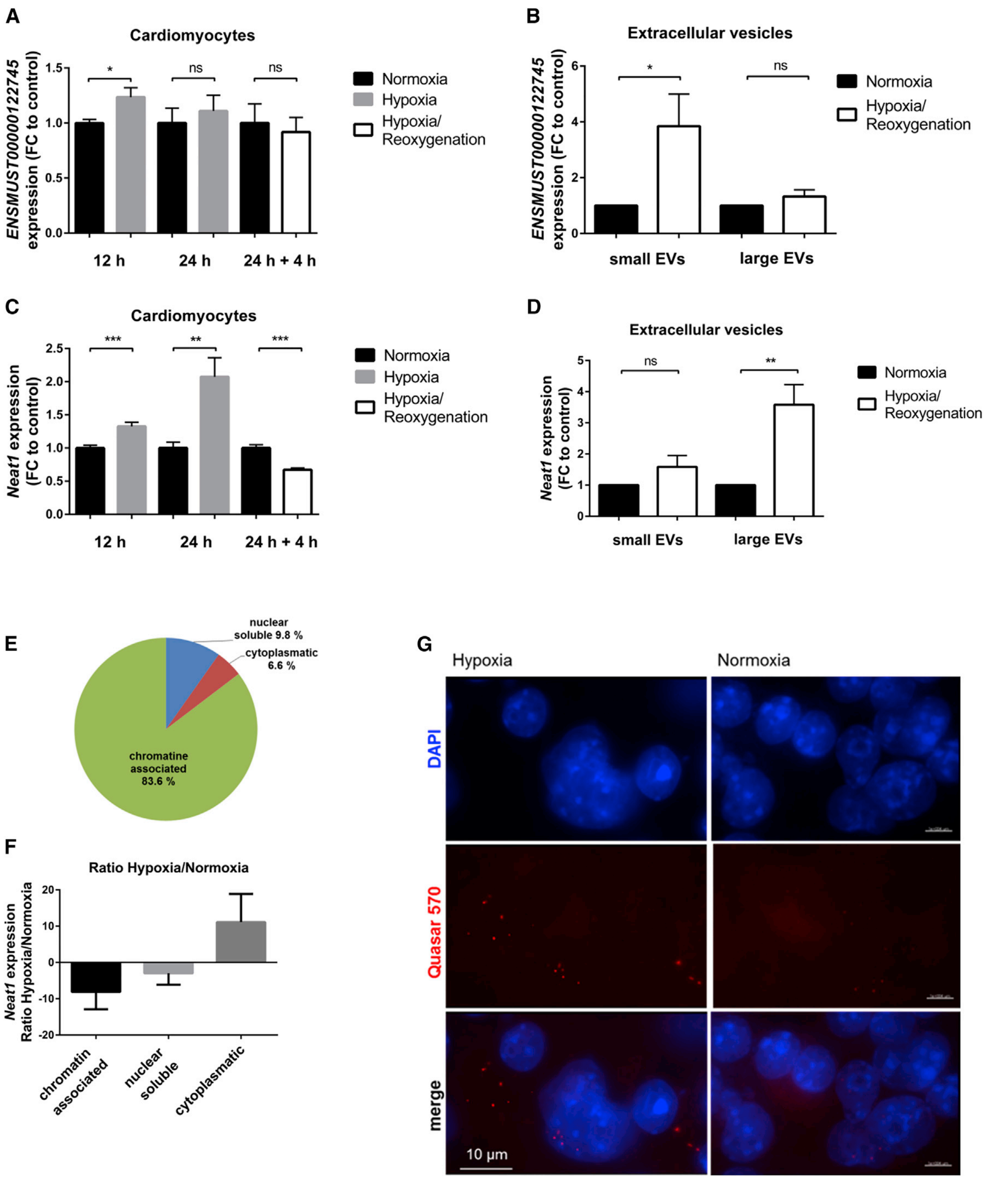

Figure 4. Characterization of Vesicle-Enriched lncRNAs

(A and C) Gene expression levels of lncRNAs *ENSMUST00000122745* (A) and *Neat1* (C) in cardiomyocytes after 12 h and 24 h of hypoxia and 24 h of hypoxia following 4 h of reoxygenation. Data are presented as mean ± SEM; n = 3 independent experiments with 2–3 replicates per experiment. (B) *ENSMUST00000122745* is enriched in cardiomyocyte-derived small vesicles. Data are presented as mean ± SEM; n = 7–12 independent experiments. (D) Hypoxia/reoxygenation triggered the release of *Neat1* via internalization in mostly large vesicles. Data are presented as mean ± SEM; n = 6–7 independent experiments. (E) Distribution of *Neat1* expression levels in subcellular compartments of cardiomyocytes. Data represent percent distribution calculated to the complete amount of transcript in qRT-PCR analysis ± SEM (n = 3 independent experiments). (F) Ratio of *Neat1* expression levels in subcellular fractions of hypoxic and normoxic cardiomyocytes. n = 3 independent experiments. (G) Representative image of RNA-FISH of normoxic and hypoxic cardiomyocytes. Nuclei are stained with DAPI (blue); *Neat1* is stained with Stellaris FISH probe Quasar 570 (red). Scale bars, 10 μm. n = 3 independent experiments. *$p \leq 0.05$; **$p \leq 0.01$; ***$p \leq 0.001$; ns, not significant, Student's t test. FC, fold change of normoxic control.

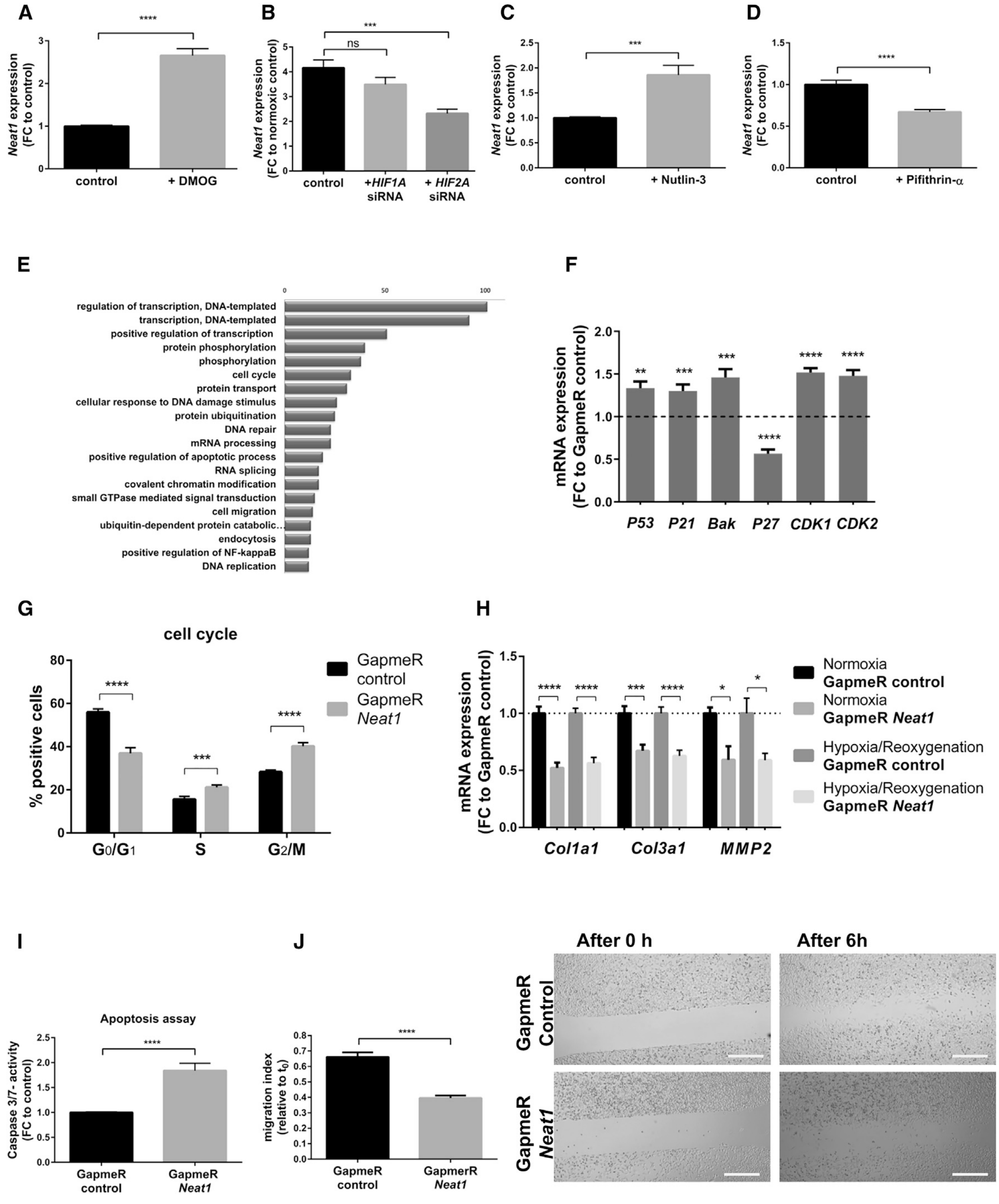

Figure 5. *Neat1* Inhibition Affects Fibroblast Function

Regulation of *Neat1* expression under normoxic and hypoxic conditions in cardiomyocytes. (A) Expression level of *Neat1* in cardiomyocytes after treatment with 1 mM DMOG. (B) Gene expression of *Neat1* following siRNA-mediated silencing of *HIF1A* or *HIF2A* under hypoxic conditions in cardiomyocytes. Cells treated with a control siRNA served as controls. (C) Increased *Neat1* expression following treatment of cardiac fibroblasts with 10 μM Nutlin-3 (activator of P53). (D) Treatment of fibroblasts with a P53 inhibitor (Pifithrin-α, 10 μM) resulted in a significant decrease of *Neat1* expression levels. Downstream effects on target cells. (E) Gene set enrichment analysis of the significantly deregulated genes (adjusted $p < 0.05$) identified by RNA-seq. The Top 20 functional terms are displayed and sorted according to the gene counts belonging to a GOTERM annotation. (F) Expression levels of *P53*, *P21*, *Bak*, *P27*, *CDK1*, and *CDK2* mRNA in fibroblasts treated with *Neat1* GapmeR or control GapmeR. Data are presented as fold change to cells treated with GapmeR control. (G) Propidium iodide staining of fibroblasts treated for 48 h with GapmeR *Neat1* and GapmeR control to analyze the cell cycle using FACS. Plot indicates percentage of positive cells in the G_0/G_1 phase, S phase and G_2/M phase. (H) Expression levels of profibrotic genes *Col1a1*, *Col3a1*, and *MMP2* in

(legend continued on next page)

we confirmed the enrichment of *ENSMUST00000122745* in cardiac sEVs and depicted elevated expression levels in cardiac sEVs originated from infarcted hearts compared to sham control hearts (Figure S2C). As overexpression of this lncRNA had no impact on fibroblast function such as apoptosis, migration, or proliferation, we focused our study on a second lncRNA candidate, *Neat1*.

The lncRNA *Neat1* is composed of different transcript variants. First experiments indicated no changes in expression level between the different isoforms; thus, we decided to analyze total *Neat1* (data not shown). We identified *Neat1* to be hypoxia responsive in cardiomyocytes (Figure 4C). We detected significantly decreased expression levels following a short time of reoxygenation. In contrast, hypoxia/reoxygenation led to elevated *Neat1* levels in cardiac lEVs compared to normoxic vesicles and their secreting cells (Figure 4D). In addition, we found *Neat1* almost exclusively chromatin associated under baseline conditions (Figure 4E), pointing to a putative role as a transcriptional regulator of gene expression also in cardiac context. When comparing the amount of *Neat1* in subcellular fractions of hypoxic and normoxic cardiomyocytes, *Neat1* was shuttled to the cytoplasm during hypoxia, which could be indicative of a different function at the post-transcriptional level during hypoxic conditions (Figure 4F). Consistent with these findings, RNA-FISH (fluorescence *in situ* hybridization) analysis of cardiomyocytes confirmed a large amount of *Neat1* in the cytoplasm of the cell during hypoxia (Figure 4G), compared to normoxic control cells (Figure 4G). Additionally, we found *Neat1* ubiquitously expressed in different mouse organs, suggesting also extracardiac effects (Figure S3).

Neat1 Is Regulated by P53 and HIF2A

Because of its dominant role in hypoxic signaling, we tested a potential contribution of the hypoxia-inducible factor (HIF) in the ischemia-derived induction of *Neat1*. Previous reports described the human homolog of *Neat1* induced during hypoxia in human breast cancer cells and to be regulated by HIF-2A.[11] However, the role of HIF in regulating *Neat1* expression in cardiac cells remains unknown. Treatment of cardiomyocytes with the HIF stabilizer dimethyloxalylglycine (DMOG) resulted in increased levels of *Neat1* (Figure 5A). We also transfected cardiac cells with small inhibitory RNAs (siRNAs) against different subunits of HIF and exposed the cells to normoxic and hypoxic conditions. The knockdown of *HIF1A* had no significant effect on *Neat1* expression, whereas inhibition of *HIF2A* resulted in decreased *Neat1* levels (Figure 5B). Besides the HIF-mediated regulation during hypoxia, *Neat1* is also highly abundant under normoxic conditions. Although HIF levels are very low under basal conditions, we examined a potential HIF-dependent regulation during normoxic conditions. Neither knockdown of *HIF1A* nor *HIF2A* isoform showed significant changes in *Neat1* expression levels (Figure S4). Recent studies revealed that the expression of human *Neat1* is regulated by P53.[12] To evaluate whether P53 might regulate the expression during normoxic conditions, cells were treated first with Nutlin-3, an inducer of P53, which led to an increased *Neat1* expression. In contrast, a decrease in *Neat1* was observed after treatment of cardiomyocytes with an inhibitor of P53 (Pifithrin-α) (Figures 5C and 5D). These results confirm that P53 can transcriptionally regulate *Neat1* expression in cardiac cells.

Silencing of *Neat1* Stalled Cell Cycle and Induced Apoptosis

We further characterized downstream effects of *Neat1* in fibroblasts. GapmeR-mediated knockdown of *Neat1* led to an ~80% reduction in expression levels (Figure S5A). As we observed a strong effect of cell loss after *Neat1* knockdown *in vitro*, we decided to investigate apoptotic pathways in cardiac cells first. Indeed, *Neat1* inhibition increased caspase-3/caspase-7 activity in both fibroblasts (Figure 5I) and cardiomyocytes (Figure S6). Thus, we hypothesized an involvement of *Neat1* in signaling pathways during apoptosis events. Next to these observations, a transcriptome analysis of fibroblasts after *Neat1* silencing was performed to gain insights into genetic changes mediated by *Neat1* knockdown. We detected a massive deregulation of transcripts, and Gene ontology (GO) term enrichment analysis revealed multiple pathways fitting into the picture that *Neat1* is a central regulator of DNA damage and repair as well as cell cycle and apoptotic pathways (Figures 5E and S7). To assess an effect on the transcriptional regulation of apoptosis-related genes, expression of *P53* and P53 targets (*Bak* and *P21*) was analyzed and revealed increased levels after *Neat1* silencing (Figure 5F). As activated P53 has pleiotrophic effects such as cell-cycle signaling pathways and RNA sequencing (RNA-seq) showed an involvement in cell-cycle regulation, we tested a potential effect of *Neat1* silencing on cell-cycle progression. Fluorescence-activated cell sorting (FACS)-based detection validated the decreased level of cells in G_0/G_1 phase and increased levels in S and G_2/M phase following *Neat1* inhibition, indicating impaired mitosis (Figure 5G). To obtain information on regulatory genes, mRNA levels of cell-cycle regulators such as *CDK1*, *CDK2*, and *P27* were measured. *Neat1* silencing resulted in decreased levels of the cell-cycle inhibitor *P27* and increased levels of cyclin-dependent kinase (CDK) *CDK1* and *CDK2* mRNA levels involved in cell-cycle progression (Figure 5F). To validate whether *Neat1* functions as a downstream effector of P53 and whether P53 inhibition can rescue the pro-apoptotic effect, we conducted *Neat1* modulation experiments with p53-deficient mouse embryonic fibroblasts (MEFs). In this cellular model, *Neat1* is also highly abundant, and its expression is, therefore, not entirely dependent on P53 abundance. Additionally, P53 deficiency did not completely attenuate apoptosis

fibroblasts transfected with GapmeR control and GapmeR *Neat1* after exposure to hypoxic conditions for 24 h following 2 h reoxygenation or 26 h normoxic conditions. (I) Caspase-3/caspase-7 activity after treatment of 3T3 cells with GapmeR *Neat1* and GapmeR control for 48 h. (J) Representative pictures of fibroblasts treated with GapmeR *Neat1* or GapmeR control and scratched to determine migration capacity. Images show time points 0 h and 6 h after scratching. Left: migration index was calculated according to: (area [0 h] − area [6 h])/area (0 h). Scale bars, 500 μm. Data are presented as mean ± SEM. n = 3 independent experiments with 3 biological replicates per independent experiment (exception: *Neat1* expression after GapmeR treatment. n = 3 different wells). *$p < 0.05$; **$p < 0.01$; ***$p \leq 0.001$; ****$p \leq 0.0001$, Student's t test (for A–D, F, and H–J) or one-way ANOVA for three groups with post hoc Tukey's multiple comparison test (for G). FC, fold change to control.

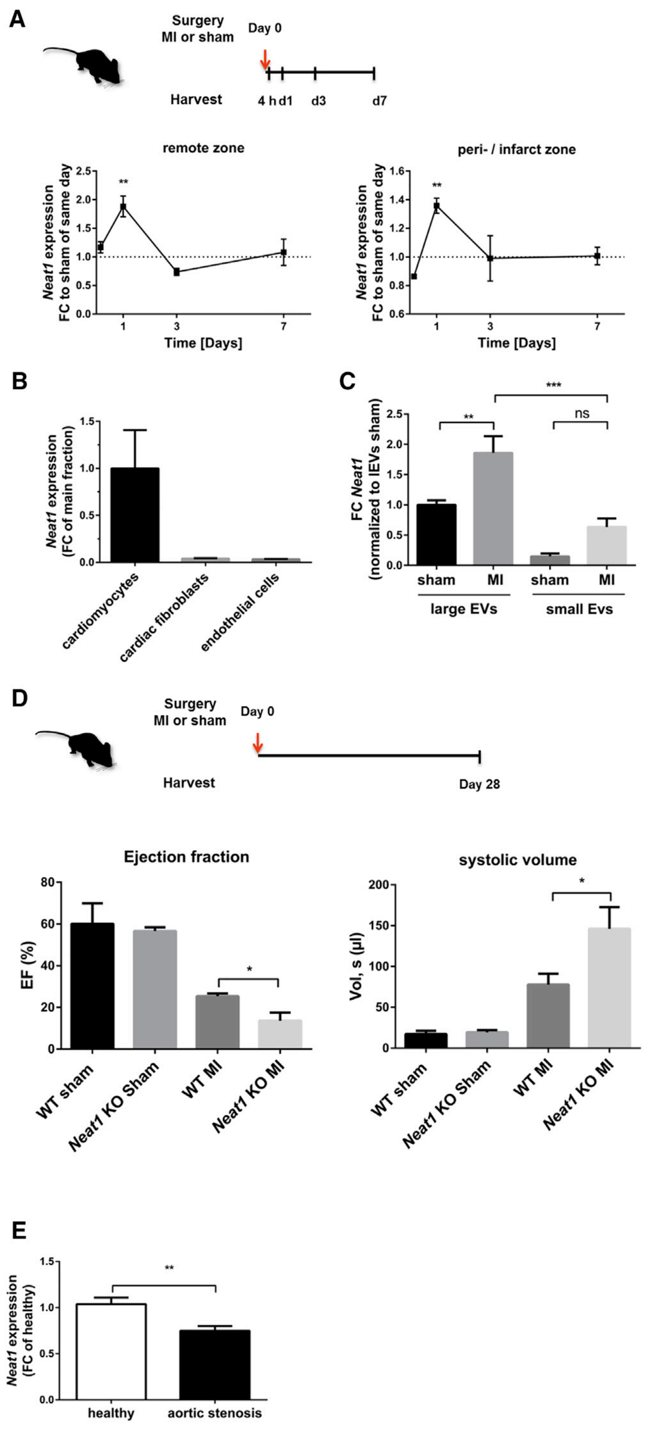

Figure 6. *Neat1* Is Transported via Large Vesicles *In Vivo*

(A) Murine hearts from C57BL6/N mice were removed 4 h to 7 days after permanent left anterior descending artery (LAD) ligation and dissected into the remote and the peri-/infarct zones. Expression level of *Neat1* was measured in both zones. Data are presented as fold change (FC) to sham-operated mice of the same day ± SEM. n = 5–6 animals per group. (B) *Neat1* expression levels in fractionated hearts from C57BL6/N mice (n = 6). Data are presented as FC of main fraction ± SEM. (C) Expression level of *Neat1* in lEVs and sEVs isolated from mouse hearts 15 h after MI. n = 3–5 animals per group. Data are presented as fold change normalized to lEVs derived from sham hearts. (D) Murine hearts of *Neat1* KO or WT mice were removed 28 days after permanent LAD ligation or sham operation, and echocardiographic parameters were assessed. n = 3–5 animals per group. EF, ejection fraction. (E) Gene expression of *Neat1* in human heart tissue of aortic stenosis patients. n = 23. Healthy patients served as controls (n = 23). $^*p \leq 0.05$; $^{**}p \leq 0.01$; $^{***}p \leq 0.001$; ns, not significant, Student's t test.

induction after *Neat1* inhibition, indicating that this is, in part, independent of P53 abundance in fibroblasts (Figure S8). In addition to genetic loss-of-function experiments, we targeted P53 with Pifithrin-α at the pharmacological level. This intervention also did not completely rescue the apoptotic effects (Figure S9) but underlined that therapeutic P53 targeting could mildly compensate for *Neat1* loss. Taken together, the data show that *Neat1* modulated the expression of P53 target genes, cell-cycle regulators and promoted cellular survival.

Neat1 Inhibition Decreased Profibrotic Genes

The key role of fibroblasts is to maintain the homeostasis of the extracellular matrix (ECM) and to control the production of ECM components.[13] Global RNA-seq experiments indicated crucial participation of *Neat1* in fibrosis development, as we found deregulated levels of fibrotic-related genes such as *AKT3*, *collagens*, *matrix metalloproteinases*, *Timp2*, *transforming growth factor* (*TGF*)*-beta receptor*, *Lox*, or *Thrombospondin 2*. Thus, we investigated putative changes in the expression of fibrotic-related markers in fibroblasts after *Neat1* inhibition and knockdown of *Neat1* led to a significant decrease in the expression levels of *Col1a1*, *Col3a1*, and *MMP2*, indicating that *Neat1* is essential for fibrosis development (Figure 5H). Another prominent feature of fibroblasts is an endogenous migration capacity involved in wound healing. Applying a scratch assay revealed decreased migration capacity after *Neat1* inhibition (Figure 5J).

Neat1 Is Shuttled via Cardiomyocyte-Derived lEVs *In Vivo*

To translate our *in vitro* findings, we studied *Neat1* abundance in different rodent models. We found *Neat1* highly enriched in cardiac myocytes (Figure 6B) and analyzed alterations in *Neat1* expression *in vivo* in a mouse model of MI. Consistent with our *in vitro* findings, *Neat1* is also deregulated in infarcted mouse hearts with a peak induction after a short time of ischemia (Figure 6A). Moreover, we confirmed that *Neat1* is also enriched in lEVs mainly originating from cardiomyocytes[14] *in vivo* and upregulated in cardiac lEVs originated from infarcted hearts compared to sham-operated conditions (Figure 6C).

In Vivo Relevance of *Neat1*

Next to the profiling of *Neat1* expression during cardiac ischemia, we studied the effects of a genetic *Neat1* loss in a murine setting of MI. Echocardiographic parameters in *Neat1* knockout (KO) mice in the context of MI underlined that knockdown of *Neat1* led to an impaired cardiac function with reduced ejection fraction and increased volume, presumably due to cardiomyocyte dysfunction 28 days (Figure 6D), as well as 14 days (Figure S10), post-MI. In addition, we assessed the

percentages of fibrotic area in *Neat1* KO versus WT mice 28 days post-MI as well as sham-operated mice via Picrosirius red staining of left ventricles. We observed a strong trend toward higher fibrosis levels in *Neat1*-deficient mice compared to those in wild-type (WT) animals (Figure S11A). Supporting this finding, gene expression of fibrosis genes such as *Col1a1*, *Col3a1*, and *CTGF* was increased (Figures S11B–S11D). In agreement with these observations, we found that knockdown of *Neat1* displayed reduced contractile force recovery after hypoxia/reoxygenation in a humanized *ex vivo* model of engineered heart tissue (Figure S12).

Finally, we investigated whether human *Neat1* is of clinical relevance and measured its expression in hypertrophic myocardial tissue of aortic stenosis patients and showed that *Neat1* is significantly decreased compared to its expression in healthy hearts, suggesting that *Neat1* may serve as a future cardiovascular therapeutic target (Figure 6E).

DISCUSSION

Within the past years, small, nanosized vesicles (extracellular vesicles) have been identified as a new facet of microcommunication between different organs or cells.[10,15] Those vesicles gain more and more attention as paracrine effectors and are considered to serve both as novel clinical biomarkers and as novel targets for therapeutic drug development. Evidence is accumulating that EVs not only contain cellular degradation products but also are able to shuttle cell-specific signature cargoes such as ncRNAs to target cells under various physiological and pathological conditions, which highlights even more their therapeutic potential in various disease settings.[16–18] Remarkably, a number of studies suggested that the unique cargo of proteins and microRNAs secreted extracellularly is required for the initiation and progression of cardiac remodeling and is capable of improving heart function. In addition, many studies reported that lncRNAs are actively secreted into the circulation during cardiac remodeling.[19–21] Whether or not such lncRNAs are actively carried by myocardial secreted vesicles in the context of cardiac ischemia remains unknown.

In the present study, we identified a novel intercellular communication route between cardiomyocytes and fibroblasts mediated by the release of EVs in the context of hypoxia. This study also demonstrates that long non-coding RNAs are incorporated into vesicles and shuttled between cardiomyocytes and fibroblasts post-infarction and identifies an indispensable role for lncRNA *Neat1* in cardiac fibroblasts. These findings strengthen future perspectives for EVs to serve as endogenous carriers for therapeutic drugs or molecules such as lncRNAs enabling personalized treatment regimes.

Specifically, we showed that cardiomyocytes are able to secrete small and larger EVs and that hypoxia triggered the release of EVs. LncRNA profiling unraveled a large amount of dysregulated lncRNAs in cardiomyocytes, lEVs, and sEVs in response to hypoxia/reoxygenation. Notably, only a small amount was packed in both large and small vesicles, indicating a selective and not random sorting process of a specific lncRNA pattern into different vesicle populations. Finally, we identified two lncRNAs (*Neat1* and *ENSMUST00000122745*) enriched in different vesicle subtypes originating from cardiomyocytes. Whereas *ENSMUST00000122745* was rather sorted into small EVs, *Neat1* was almost exclusively transported via lEVs. In line with our *in vitro* results, we also evaluated that both lncRNAs are deregulated *in vivo* in infarcted mouse hearts and confirmed that, *in vivo*, *Neat1* is also shuttled via lEVs and that *ENSMUST00000122745* is shuttled via small vesicle transfer. Although evidence is accumulating that lncRNAs can be incorporated into EVs, the mechanism for how cells select specific lncRNAs for extracellular release remains unclear.

Neat1 is a well-characterized lncRNA and was proven to play a major role in the formation of nuclear paraspeckles.[22] In addition, several studies showed altered expression levels in human malignant diseases, including gastric cancer, lung cancer, and breast cancer.[11,23] As *Neat1* induction is associated with cellular stress conditions, it has been speculated that *Neat1* is involved in the cellular stress response. In our study, we identified *Neat1* to be a hypoxia-sensitive lncRNA in cardiomyocytes, in agreement with studies of human *Neat1* in cancer cells. Recent studies have identified a large number of lncRNAs such as *H19* and *HOTAIR* to be regulated by hypoxia, specifically by the transcription factor HIF.[24,25] In line with our results, a recent study reported *Neat1* regulation by HIF2A.[11] Mechanistically, we could show here that *Neat1* is a downstream target of HIF2A under hypoxic conditions. Although both isoforms have the same binding site in the *Neat1* promoter region and are binding together in a complex to the HIF response elements, the hypoxic induction of *Neat1* is predominantly regulated by HIF2A. At the basal level, *Neat1* is also highly abundant. Recently, some reports explored a P53 dependency of *Neat1* expression for the human homolog and provided evidence for the binding of P53 to the promoter region.[12,26] Consistent with these studies, we observed that inactivation of P53 decreased *Neat1* expression and activation of elevated *Neat1* levels in murine cardiac cells. Some studies already provided evidence that lncRNAs not only are activated or suppressed by P53 but can also mediate the downstream effects of P53 by transcriptional regulation of target genes in the P53 pathways.[27,28] Therefore, we speculated on participation in P53 signaling routes. Indeed, we found higher levels of apoptosis in fibroblasts and cardiomyocytes as well as an increased gene expression of the proapoptotic factor *Bak* after *Neat1* knockdown. We next evaluated whether P53 deficiency could attenuate the intrinsic apoptotic signaling induced by *Neat1* loss. However, P53 loss did not completely rescue for the phenotype, indicating also independent regulatory mechanisms. Besides the pro-apoptotic consequences after *Neat1* silencing, we identified an involvement in cell-cycle progression. Cardiac cells arrest in the G_2/M phase and do not complete or enter into mitosis, presumably via higher expression of cell-cycle regulators such as *CDK1* and *CDK2*.

We also evaluated the function of *Neat1* in fibroblasts via RNA-seq and confirmed that its inhibition strongly affects key fibroblast features such as response to DNA damage, DNA repair, apoptosis signaling, and fibrosis. We further validated the main characteristics

and found that fibroblasts lose their ability to migrate and repress fibrotic-related genes upon silencing *Neat1*, suggesting that *Neat1* is essential for the expression of ECM components and the maintenance of heart function. In accordance with our results, recent studies observed that *Neat1* affects ECM protein secretion in mesangial cells[29] and collagen expression in liver fibrosis.[30] In-depth analysis of RNA-seq data revealed a key cell regulator, Akt, which can also be connected to fibrosis, as one of the top 10 deregulated genes after *Neat1* silencing. In line with this, we also detected several other genes that can be linked to AKT pathways to be deregulated, including phosphatidylinositol 3-kinase (PI3K) components and PTEN, indicating that *Neat1* controls subsequent downstream responses including cell survival, growth, fibrosis, proliferation, and migration via modulation of AKT activation. In agreement with this hypothesis, a recent work demonstrated that *Neat1* repression led to decreased proliferation and fibrosis in diabetic nephropathy via activation of the Akt/mTOR signaling pathway.[31]

To further investigate whether cardiomyocyte-derived vesicles are crucially involved in the paracrine action, we validated that the conditioned medium of hypoxic cardiomyocytes triggered a profibrotic response and that lncRNA-enriched cardiomyocyte-derived vesicles are taken up into fibroblasts. Whether the uptake and subsequent release of EVs to the cytoplasm contribute to cytoplasmic NEAT1 function still needs to be determined.

Finally, we evaluated the systemic function of *Neat1 in vivo* in a mouse model of MI. Cardiomyocyte apoptosis is a major event directly after MI, and, indeed, *Neat1* loss-of-function caused cardiomyocyte apoptosis *in vitro*. In line with this, *Neat1* expression was mainly increased 24 h after MI, indicating an involvement in both apoptosis and necrosis. Because we observed global impact on cardiac remodeling, including alteration of cardiomyocyte and fibroblast biology, we suggest studying a cardiomyocyte-specific KO for EV generation and outcome. Recently, it was also shown that *Neat1* can modulate immune cell function post-MI,[32] highlighting another aspect of cardiac remodeling. Genetic loss of *Neat1* triggered a worsening of heart function in *Neat1* KO animals post-MI. A future therapeutic strategy would not be lowering cardiac *Neat1* level but, rather, overexpressing this lncRNA and applying *Neat1*-enriched EVs to the heart in a model of MI. Despite the fact that the research field of EVs has emerged in previous years from initial *in vitro* studies and pre-clinical reports to early clinical trials, we are currently still in the infancy of understanding the precise mechanism and their exact role. In addition, several milestones have to be overcome before entering clinical settings, such as optimizing isolation techniques, heart-specific delivery, and finding a suitable source of vesicles for large-scale production. However, as EVs have been shown to be capable of improving heart function as well as heart regeneration and are released into the circulation in patients, the use of vesicles as biomarkers or the therapeutic modulation by, e.g., drug loading into vesicles is very promising. In addition, several studies reported that *Neat1* can serve as a predictor of poor clinical outcome and might be an important prognostic biomarker in different cancer types.[33,34] We identified that human *Neat1* is of interest in clinical scenarios of cardiovascular diseases, as we detected decreased levels in aortic stenosis patients. Despite these findings, one has to keep in mind that these studies are only performed in a small cohort and have to be validated in larger settings. It would be also of great importance to determine the expression during the progression of the disease in order to evaluate the potential to serve as a predictor of poor survival or overall clinical outcome.

Collectively, this study provides a new concept of a paracrine cardiac communication system during hypoxic stress conditions mediated by lncRNA-enriched vesicles and opens a wide range of future diagnostic and therapeutic options.

MATERIALS AND METHODS

A detailed description of methods can be found in the Supplemental Materials and Methods.

Cell-Culture Experiments

HL-1 cells, a murine atrial cardiac muscle cell line, were cultured in Claycomb medium (Sigma Aldrich, Munich, Germany) supplemented with 10% fetal bovine serum (FBS) (Sigma Aldrich, Munich, Germany), 1% penicillin/streptomycin (100 U/mL; 100 μg/mL; Sigma Aldrich), 0.1 mM norepinephrine (Sigma Aldrich, Munich, Germany), and 2 mM L-glutamine (Sigma Aldrich, Munich, Germany). NIH 3T3 mouse fibroblasts were cultured in DMEM supplemented with 10% FBS (Sigma Aldrich, Munich, Germany) and 1% penicillin/streptomycin (100 U/mL; 100 μg/mL; Sigma Aldrich, Munich, Germany). The cells were cultured in a humidified incubator with 21% O_2 and 5% CO_2 at 37°C. For loss-of-function studies, LNA GapmeRs (Exiqon, part of QIAGEN, Venlo, the Netherlands) against *Neat1* (5′-TACCATCAGCCTTTAG-3′) or a negative control (5′-AACACGTCTATACGC-3′) were used. NIH 3T3 cells were transiently transfected with 50 nM GapmeR using X-tremeGENE HP Transfection Reagent (Sigma-Aldrich, Munich, Germany). In order to evaluate the upstream mechanisms of *Neat1* expression, HL-1 cells were transfected with siRNA against HIF1A (Santa Cruz Biotechnology, Santa Cruz, CA, USA, # sc-35562), HIF2A (Thermo Fisher Scientific, #4390771, Darmstadt, Germany), or the corresponding negative controls using Lipofectamine 2000 (Invitrogen, Karlsruhe, Germany). For hypoxia experiments, the HL-1 cells were cultured in media supplemented with 5% exosome-depleted FBS after incubation in fully supplemented medium (10% FBS) for 24 h. To induce hypoxia, the cells were grown in a humidified incubator with 5% CO_2 and 0.2% O_2 at 37°C for 24 h following 4 h reoxygenation under normoxic conditions with 21% O_2, if not indicated otherwise. The cells that served as a control group were cultured for the same time period at normoxic conditions.

Extracellular Vesicle Isolation

The supernatant of HL-1 cells was collected, and extracellular vesicles were purified by differential centrifugation steps and

ultracentrifugation. In brief, the conditioned medium was centrifuged at 300 × *g* for 10 min at 4°C to remove cellular debris, 2,000 × *g* for 20 min at 4°C to isolate apoptotic bodies, and 16,500 × *g* for 20 min at 4°C to purify lEVs. After the isolation of lEVs, the remaining supernatant was filtered through a 0.22-μm filter. For small EV isolation, the supernatant was additionally ultracentrifuged at 100,000 × *g* for 70 min. Protein content was measured using the MicroBCA protein assay (Thermo Fisher Scientific, Darmstadt, Germany). The lEVs and sEVs were analyzed by western blot as previously described, and nanoparticle tracking analysis was carried out using the LM10 unit (Nanosight). For uptake experiments, EVs were labeled with the PKH67 Green Fluorescent Cell Linker Kit (Sigma-Aldrich, Munich, Germany) according to the manufacturer's protocol, with minor modifications. Briefly, 7.5 μg of an EV subtype was mixed with 1 mL diluent C. Then, 4 μL PKH67 dye was added to 1 mL diluent C (2× dye solution) and gently mixed with the EV/diluent C solution and incubated for 4 min. Subsequently, 1 mL 0.5% BSA in PBS was added and incubated for 5 min to allow binding of the dye. The solution was centrifuged for 70 min at 100,000 × *g* (for sEVs) or 20 min at 16,500 × *g* (for lEVs), and the pellet was diluted in cell-culture medium. 7.5 μg EVs were used for further experiments.

Animal Studies

Animal studies were performed in accordance with the relevant guidelines and regulations of the responsible authorities (governmental animal ethics committee LAVES). For all animal experiments, we used 8- to 10-week-old male C57Bl6 mice. *Neat1* KO have been described previously.[35]

Permanent Ligation of LAD (Myocardial Infarction)

Mice were anesthetized by 2%–3% isoflurane mixed with O_2 in an induction chamber. The neck and chest area was shaved and disinfected with betadine and alcohol. Mice were intubated via intratracheal cannula and fixed in the supine position to a heating pad (temperature was maintained at 37°C) and under an operating microscope. The trachea cannula was then attached to a small animal respirator, and the animal was ventilated at 100/min with a 150-μL stroke volume. After adequate analgesia (Torbugesic Vet [Butorphanol] and Novalgin [Metamizol]), a horizontal skin incision approximately 0.5–1.0 cm in length was made laterally over the second and third ribs. After thoracotomy, the thyroid and lung were retracted, allowing for visualization of the anterior wall of the left ventricle under low-power magnification. A 7/0 silk suture was inserted in the myocardium and passed under the left anterior descending branch of the left anterior descending artery (LAD), and the suture was tied around. Significant color changes at the ischemic area were considered indicative of successful coronary occlusion. The sham procedure was identical, except that the coronary vessel was not ligated.

Statistics

All experiments were performed as described in the corresponding figure legends. In general, *in vitro* experiments were performed in 3 independent experiments with 3 biological replicates/wells per independent experiment (n = 3) unless stated otherwise. Data are presented as mean of independent experiments/independent samples ± SEM. GraphPad Prism 6 (GraphPad Software) was used to perform statistical analysis. For statistical comparison of two groups, unpaired two-tailed Student's t test was used. For comparison of three or more groups, a one-way ANOVA followed by Tukey's post-test was applied. A p value of 0.05 or lower was considered to be significant in all experiments.

Human Tissue Sampling

RNA was isolated from human cardiac tissue of the left ventricle from patients subjected to aortic valve replacement or from healthy donor hearts. For this study, expression of the gene of interest was measured in 23 aortic stenosis patients (male:female, 15:8; mean age, 69.65 ± 17.48) and 23 healthy patients (male:female, 14:9; mean age, 38.61 ± 12.96). Approval of the study was given by the institutional committees of the University of Würzburg, Würzburg, Germany, and the University of Hamburg, Hamburg, Germany.

SUPPLEMENTAL INFORMATION

Supplemental Information can be found online at https://doi.org/10.1016/j.omtn.2019.09.003.

AUTHOR CONTRIBUTIONS

T.T., J.F., and C.B.: conception, design and interpretation of the data, and revision of the manuscript; F.K.: conception, data analysis and interpretation, and drafting the manuscript; K.X., C.M.B., X.L., B.S., S.H.-B., A.F., M.N.H., T.E., S.F., C.G., K.S., A.J., A.P., S.D., S.M.R.-B., S.S., S.M., and K.S.: analysis and interpretation of data and final approval of the manuscript.

CONFLICT OF INTERESTS

T.T. is a founder of and holds shares in Cardior Pharmaceuticals. The other authors report no competing interests.

ACKNOWLEDGMENTS

We acknowledge the support of Stefanie Dimmeler and Nicolas Jaé (Frankfurt, Germany) for the nanoparticle tracking of vesicles. We also acknowledge Oliver Dittrich-Breiholz for the RNA-seq data. We acknowledge funding by the Deutsche Forschungsgemeinschaft (DFG 903/19-1 to T.T.), the ERC Consolidator Grant Longheart (to T.T.), and the French National Agency (ANR-16-CE92-0032-02 to C.M.B.).

REFERENCES

1. Dobaczewski, M., Gonzalez-Quesada, C., and Frangogiannis, N.G. (2010). The extracellular matrix as a modulator of the inflammatory and reparative response following myocardial infarction. J. Mol. Cell. Cardiol. *48*, 504–511.

2. Frantz, S., Bauersachs, J., and Ertl, G. (2009). Post-infarct remodelling: contribution of wound healing and inflammation. Cardiovasc. Res. *81*, 474–481.

3. Burchfield, J.S., Xie, M., and Hill, J.A. (2013). Pathological ventricular remodeling: mechanisms: part 1 of 2. Circulation *128*, 388–400.

4. Takeda, N., and Manabe, I. (2011). Cellular interplay between cardiomyocytes and nonmyocytes in cardiac remodeling. Int. J. Inflamm. *2011*, 535241.

5. Kakkar, R., and Lee, R.T. (2010). Intramyocardial fibroblast myocyte communication. Circ. Res. *106*, 47–57.
6. Kuwabara, Y., Ono, K., Horie, T., Nishi, H., Nagao, K., Kinoshita, M., Watanabe, S., Baba, O., Kojima, Y., Shizuta, S., et al. (2011). Increased microRNA-1 and microRNA-133a levels in serum of patients with cardiovascular disease indicate myocardial damage. Circ. Cardiovasc. Genet. *4*, 446–454.
7. Cheng, Y., Wang, X., Yang, J., Duan, X., Yao, Y., Shi, X., Chen, Z., Fan, Z., Liu, X., Qin, S., et al. (2012). A translational study of urine miRNAs in acute myocardial infarction. J. Mol. Cell. Cardiol. *53*, 668–676.
8. Bang, C., Batkai, S., Dangwal, S., Gupta, S.K., Foinquinos, A., Holzmann, A., Just, A., Remke, J., Zimmer, K., Zeug, A., et al. (2014). Cardiac fibroblast-derived microRNA passenger strand-enriched exosomes mediate cardiomyocyte hypertrophy. J. Clin. Invest. *124*, 2136–2146.
9. Théry, C., Witwer, K.W., Aikawa, E., Alcaraz, M.J., Anderson, J.D., Andriantsitohaina, R., Antoniou, A., Arab, T., Archer, F., Atkin-Smith, G.K., et al. (2018). Minimal information for studies of extracellular vesicles 2018 (MISEV2018): a position statement of the International Society for Extracellular Vesicles and update of the MISEV2014 guidelines. J. Extracell. Vesicles *7*, 1535750.
10. Bang, C., Antoniades, C., Antonopoulos, A.S., Eriksson, U., Franssen, C., Hamdani, N., Lehmann, L., Moessinger, C., Mongillo, M., Muhl, L., et al. (2015). Intercellular communication lessons in heart failure. Eur. J. Heart Fail. *17*, 1091–1103.
11. Choudhry, H., Albukhari, A., Morotti, M., Haider, S., Moralli, D., Smythies, J., Schödel, J., Green, C.M., Camps, C., Buffa, F., et al. (2015). Tumor hypoxia induces nuclear paraspeckle formation through HIF-2α dependent transcriptional activation of NEAT1 leading to cancer cell survival. Oncogene *34*, 4482–4490.
12. Blume, C.J., Hotz-Wagenblatt, A., Hüllein, J., Sellner, L., Jethwa, A., Stolz, T., Slabicki, M., Lee, K., Sharathchandra, A., Benner, A., et al. (2015). p53-dependent non-coding RNA networks in chronic lymphocytic leukemia. Leukemia *29*, 2015–2023.
13. Kong, P., Christia, P., and Frangogiannis, N.G. (2014). The pathogenesis of cardiac fibrosis. Cell. Mol. Life Sci. *71*, 549–574.
14. Loyer, X., Zlatanova, I., Devue, C., Yin, M., Howangyin, K.Y., Klaihmon, P., Guerin, C.L., Kheloufi, M., Vilar, J., Zannis, K., et al. (2018). Intra-cardiac release of extracellular vesicles shapes inflammation following myocardial infarction. Circ. Res. *123*, 100–106.
15. Adamiak, M., and Sahoo, S. (2018). Exosomes in myocardial repair: advances and challenges in the development of next-generation therapeutics. Mol. Ther. *26*, 1635–1643.
16. Viereck, J., and Thum, T. (2017). Circulating noncoding RNAs as biomarkers of cardiovascular disease and injury. Circ. Res. *120*, 381–399.
17. Valadi, H., Ekström, K., Bossios, A., Sjöstrand, M., Lee, J.J., and Lötvall, J.O. (2007). Exosome-mediated transfer of mRNAs and microRNAs is a novel mechanism of genetic exchange between cells. Nat. Cell Biol. *9*, 654–659.
18. Sahoo, S., and Losordo, D.W. (2014). Exosomes and cardiac repair after myocardial infarction. Circ. Res. *114*, 333–344.
19. Kumarswamy, R., Bauters, C., Volkmann, I., Maury, F., Fetisch, J., Holzmann, A., Lemesle, G., de Groote, P., Pinet, F., and Thum, T. (2014). Circulating long noncoding RNA, LIPCAR, predicts survival in patients with heart failure. Circ. Res. *114*, 1569–1575.
20. Vausort, M., Wagner, D.R., and Devaux, Y. (2014). Long noncoding RNAs in patients with acute myocardial infarction. Circ. Res. *115*, 668–677.
21. Yang, Y., Cai, Y., Wu, G., Chen, X., Liu, Y., Wang, X., Yu, J., Li, C., Chen, X., Jose, P.A., et al. (2015). Plasma long non-coding RNA, CoroMarker, a novel biomarker for diagnosis of coronary artery disease. Clin. Sci. (Lond) *129*, 675–685.
22. Hirose, T., Virnicchi, G., Tanigawa, A., Naganuma, T., Li, R., Kimura, H., Yokoi, T., Nakagawa, S., Bénard, M., Fox, A.H., and Pierron, G. (2014). NEAT1 long noncoding RNA regulates transcription via protein sequestration within subnuclear bodies. Mol. Biol. Cell *25*, 169–183.
23. Ma, Y., Liu, L., Yan, F., Wei, W., Deng, J., and Sun, J. (2016). Enhanced expression of long non-coding RNA NEAT1 is associated with the progression of gastric adenocarcinomas. World J. Surg. Oncol. *14*, 41.
24. Wu, W., Hu, Q., Nie, E., Yu, T., Wu, Y., Zhi, T., Jiang, K., Shen, F., Wang, Y., Zhang, J., and You, Y. (2017). Hypoxia induces H19 expression through direct and indirect Hif-1α activity, promoting oncogenic effects in glioblastoma. Sci. Rep. *7*, 45029.
25. Zhou, C., Ye, L., Jiang, C., Bai, J., Chi, Y., and Zhang, H. (2015). Long noncoding RNA HOTAIR, a hypoxia-inducible factor-1α activated driver of malignancy, enhances hypoxic cancer cell proliferation, migration, and invasion in non-small cell lung cancer. Tumour Biol. *36*, 9179–9188.
26. Adriaens, C., Standaert, L., Barra, J., Latil, M., Verfaillie, A., Kalev, P., Boeckx, B., Wijnhoven, P.W., Radaelli, E., Vermi, W., et al. (2016). p53 induces formation of NEAT1 lncRNA-containing paraspeckles that modulate replication stress response and chemosensitivity. Nat. Med. *22*, 861–868.
27. Huarte, M., Guttman, M., Feldser, D., Garber, M., Koziol, M.J., Kenzelmann-Broz, D., Khalil, A.M., Zuk, O., Amit, I., Rabani, M., et al. (2010). A large intergenic noncoding RNA induced by p53 mediates global gene repression in the p53 response. Cell *142*, 409–419.
28. Chaudhary, R., Gryder, B., Woods, W.S., Subramanian, M., Jones, M.F., Li, X.L., Jenkins, L.M., Shabalina, S.A., Mo, M., Dasso, M., et al. (2017). Prosurvival long noncoding RNA *PINCR* regulates a subset of p53 targets in human colorectal cancer cells by binding to Matrin 3. eLife *6*, e23244.
29. Wang, X., Xu, Y., Zhu, Y.C., Wang, Y.K., Li, J., Li, X.Y., Ji, T., and Bai, S.J. (2019). LncRNA NEAT1 promotes extracellular matrix accumulation and epithelial-to-mesenchymal transition by targeting miR-27b-3p and ZEB1 in diabetic nephropathy. J. Cell. Physiol. *234*, 12926–12933.
30. Yu, F., Jiang, Z., Chen, B., Dong, P., and Zheng, J. (2017). NEAT1 accelerates the progression of liver fibrosis via regulation of microRNA-122 and Kruppel-like factor 6. J. Mol. Med. (Berl.) *95*, 1191–1202.
31. Huang, S., Xu, Y., Ge, X., Xu, B., Peng, W., Jiang, X., Shen, L., and Xia, L. (2019). Long noncoding RNA NEAT1 accelerates the proliferation and fibrosis in diabetic nephropathy through activating Akt/mTOR signaling pathway. J. Cell. Physiol. *234*, 11200–11207.
32. Gast, M., Rauch, B., Haghikia, A., Nakagawa, S., Haas, J., Stroux, A., Schmidt, D., Schumann, P., Weiss, S., Jensen, L., et al. (2019). Long noncoding RNA NEAT1 modulates immune cell functions and is suppressed in early onset myocardial infarction patients. Cardiovasc. Res. cvz085.
33. Ning, L., Li, Z., Wei, D., Chen, H., and Yang, C. (2017). LncRNA, NEAT1 is a prognosis biomarker and regulates cancer progression via epithelial-mesenchymal transition in clear cell renal cell carcinoma. Cancer Biomark. *19*, 75–83.
34. Wu, Y., Yang, L., Zhao, J., Li, C., Nie, J., Liu, F., Zhuo, C., Zheng, Y., Li, B., Wang, Z., and Xu, Y. (2015). Nuclear-enriched abundant transcript 1 as a diagnostic and prognostic biomarker in colorectal cancer. Mol. Cancer *14*, 191.
35. Nakagawa, S., Naganuma, T., Shioi, G., and Hirose, T. (2011). Paraspeckles are subpopulation-specific nuclear bodies that are not essential in mice. J. Cell Biol. *193*, 31–39.

OMTN, Volume *18*

Supplemental Information

Long Noncoding RNA-Enriched Vesicles Secreted by Hypoxic Cardiomyocytes Drive Cardiac Fibrosis

Franziska Kenneweg, Claudia Bang, Ke Xiao, Chantal M. Boulanger, Xavier Loyer, Stephane Mazlan, Blanche Schroen, Steffie Hermans-Beijnsberger, Ariana Foinquinos, Marc N. Hirt, Thomas Eschenhagen, Sandra Funcke, Stevan Stojanovic, Celina Genschel, Katharina Schimmel, Annette Just, Angelika Pfanne, Kristian Scherf, Susann Dehmel, Stella M. Raemon-Buettner, Jan Fiedler, and Thomas Thum

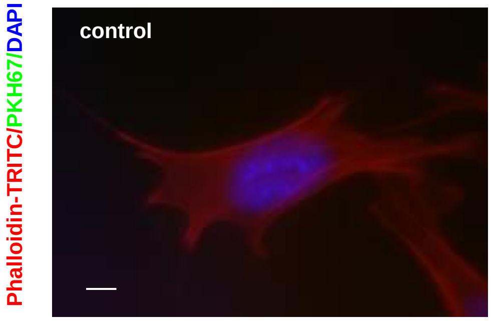

Figure 1: Microscope image of fibroblasts. Vehicle control (PBS) was stained with a green fluorescent dye (PKH67), co-cultured with fibroblasts for 20h at 37 °C and images were taken. Fibroblasts were stained with DAPI (blue) and Phalloidin-TRITC (red). Scale bar=5 µm

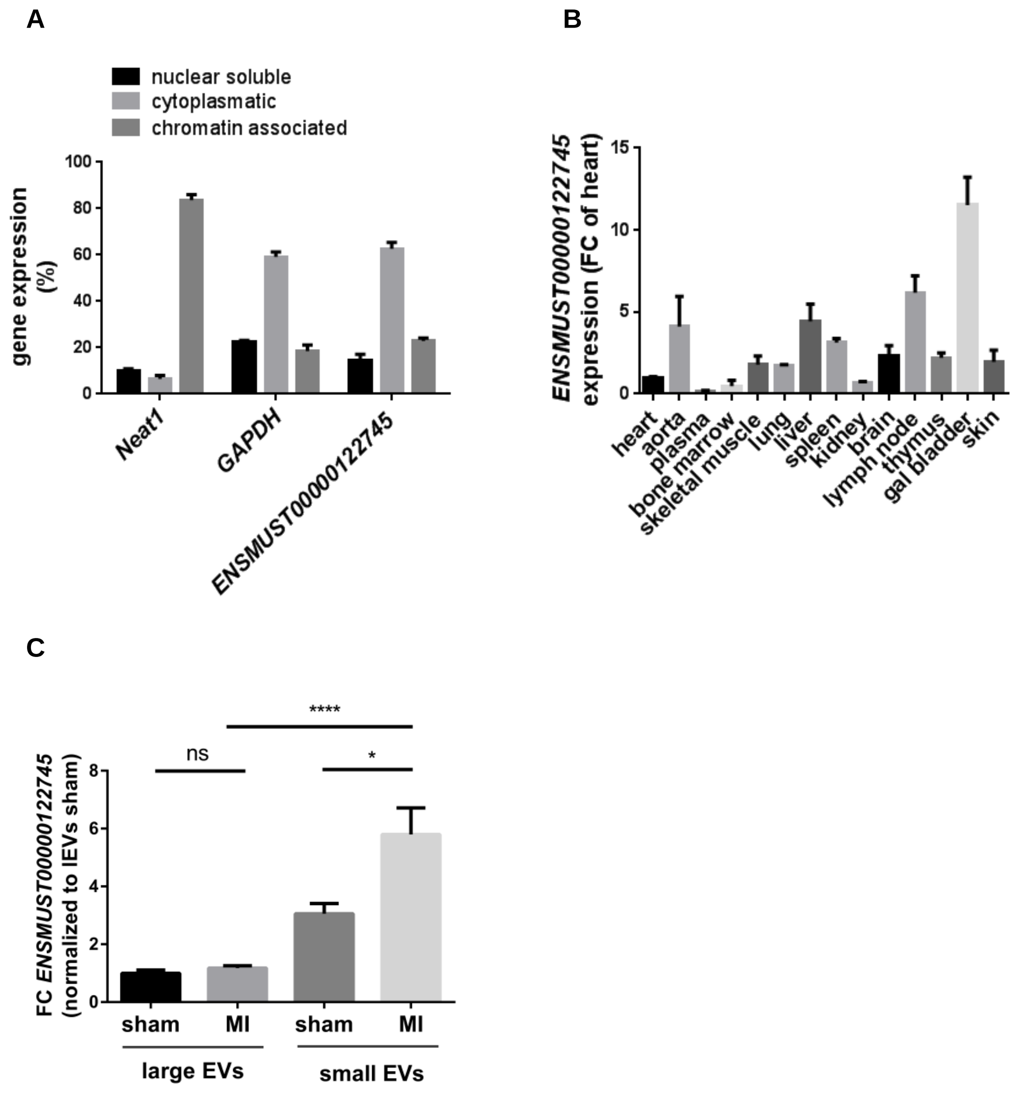

Figure 2: Characterization of lncRNA *ENSMUST00000122745*

(**A**) Distribution of *ENSMUST00000122745* expression levels in subcellular compartments of cardiomyoctes. Data are % distribution calculated to the complete amount of transcript in qRT-PCR analysis ± SEM (n=3 independent experiments). (**B**) Gene expression of *ENSMUST00000122745* in murine organs of C57BL6 mice. Data are presented as mean ±SEM. N=3 animals. (**C**) Gene expression of *ENSMUST00000122745* in large and small Evs isolated from mouse hearts 15 h after myocardial infarction (MI) was quantified by qRT-PCR. n=3 to 5 animals per group. Data are presented as fold change normalized to microvesicles derived from sham hearts. FC=foldchange * $p \leq 0.05$; **** $p \leq 0.0001$ Student´s t-test

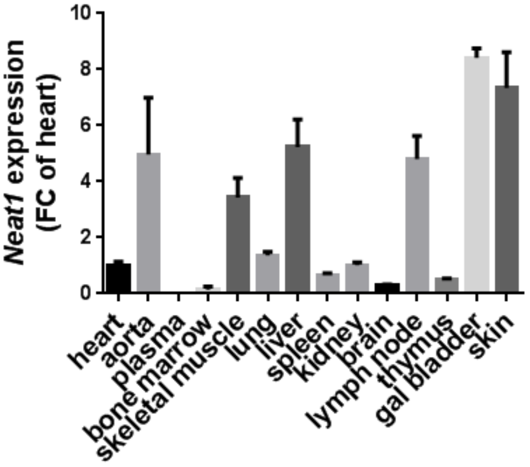

Figure 3: Characterization of *Neat1*

Distribution of *Neat1* in murine organs of C57BL6 mice. Data are presented as mean ±SEM. N=3 animals. FC=fold change of heart

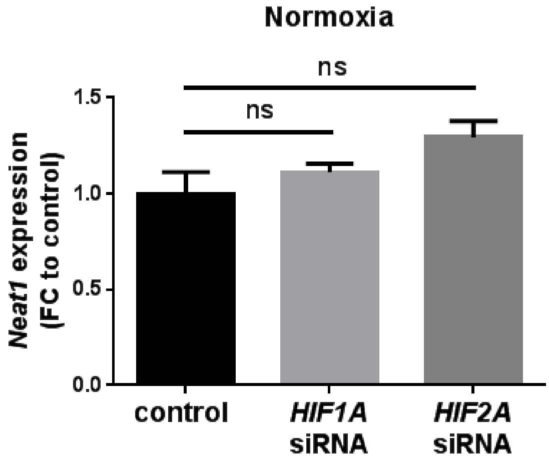

Figure 4: Regulation of *Neat1* expression

Gene expression level of *Neat1* following siRNA-mediated silencing of HIF1A or HIF2A under normoxic conditions (21% O_2) in cardiomyocytes. Data are presented as mean ±SEM. N=3

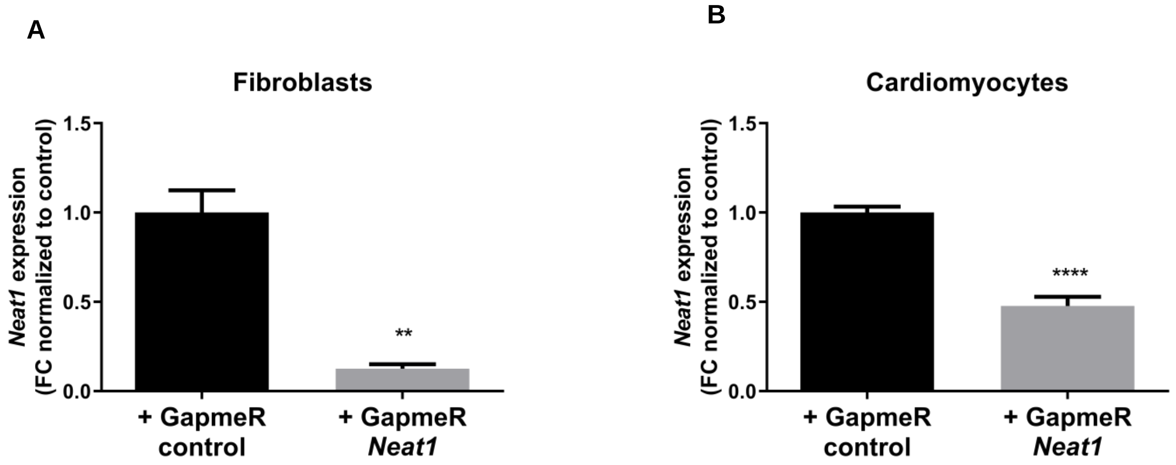

Figure 5: Knockdown efficiency of *Neat1*. Expression levels of Neat1 in cardiomyocytes (A) and fibroblasts (B) treated with GapmeR *Neat1* or control GapmeR. Data are presented as mean fold change ±SEM. N=3; ** $p \leq 0.01$; **** $p \leq 0.0001$ Student´s t-test

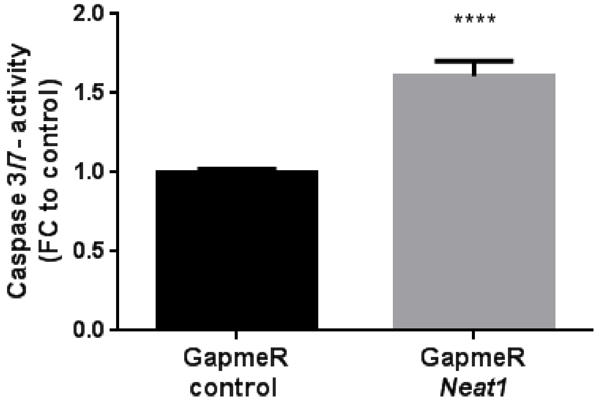

Figure 6: Apoptosis levels in cardiomyocytes
Caspase 3/7-activity was measured after treatment of cardiomyocytes with GapmeR *Neat1* and GapmeR control. Data are presented as mean ±SEM. N=3. **** $p \leq 0.0001$ Student´s t-test

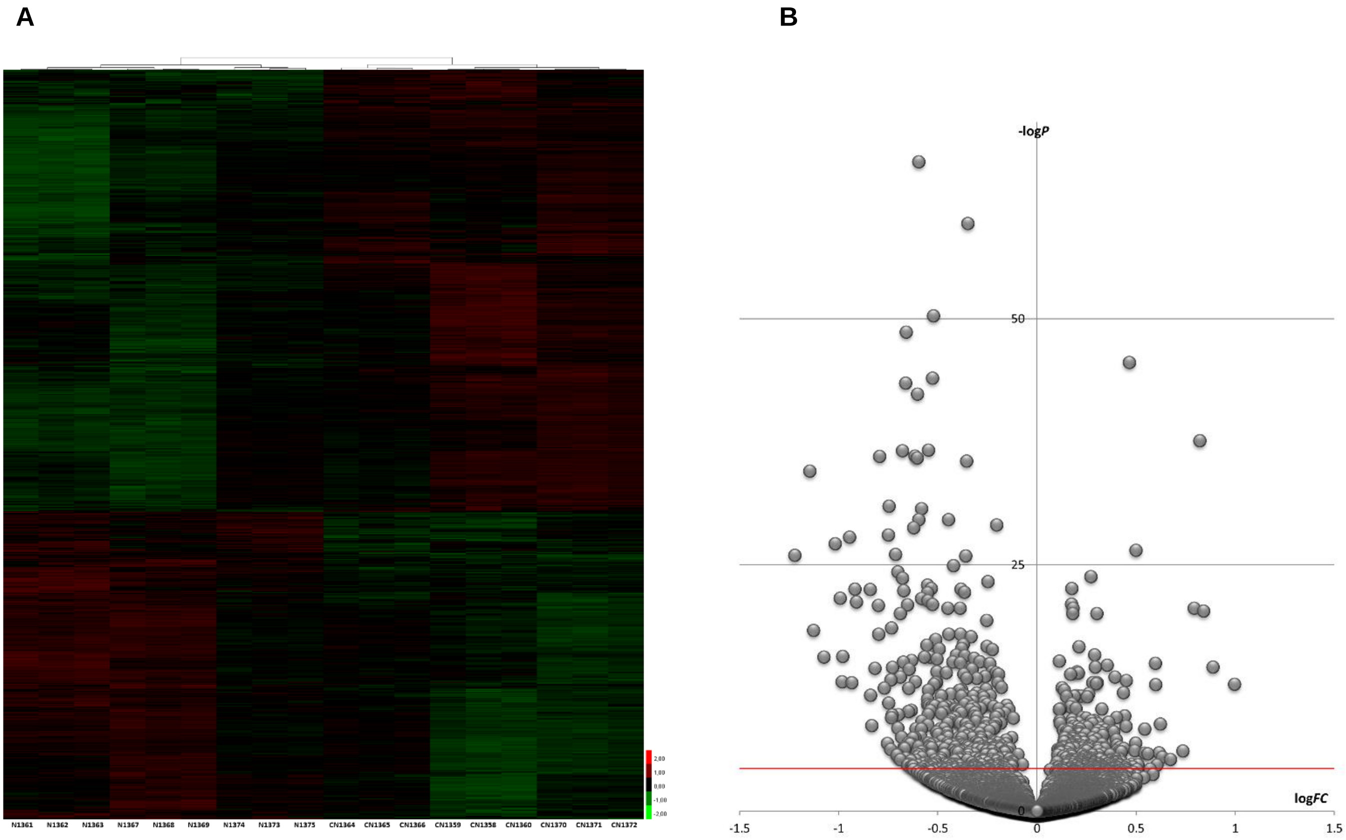

Figure 7: RNA-Sequencing data.

(A) Heatmap of the normalized expression level of the significantly regulated genes (padj<0.05) in fibroblasts treated with GapmeR control or GapmeR *Neat1*. n=3 independent experiment with 3 biological replicates. (**B**) Volcano plot of the differential expression analysis results from RNA-seq data. X-axis: log transformed fold change of the gene expression level between Gapmer *Neat1* treated samples and GapmeR controls. Y-axis: log transformed adjusted p-value.

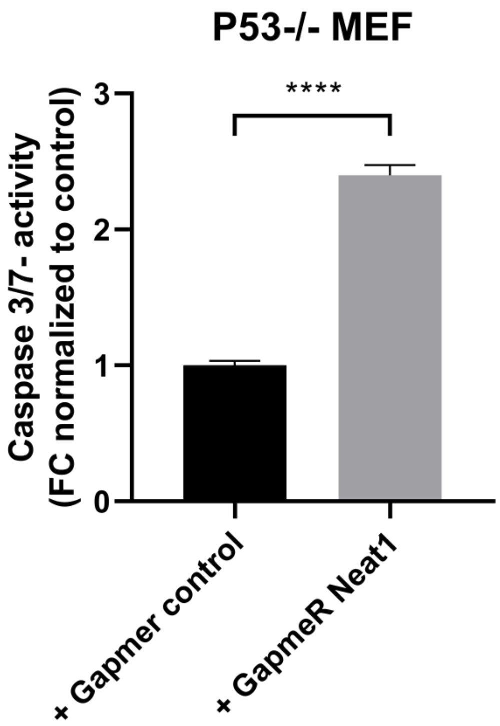

Figure 8: Apoptosis assessment in P53-/- MEFs
Caspase 3/7-activity was measured after treatment of P53-deficient mouse embryonic fibroblasts (MEF) with GapmeR *Neat1* and GapmeR control. Data are presented as mean ±SEM. N=3 independent experiments with 3 biological replicates. **** p≤0.0001; Student´s t-test

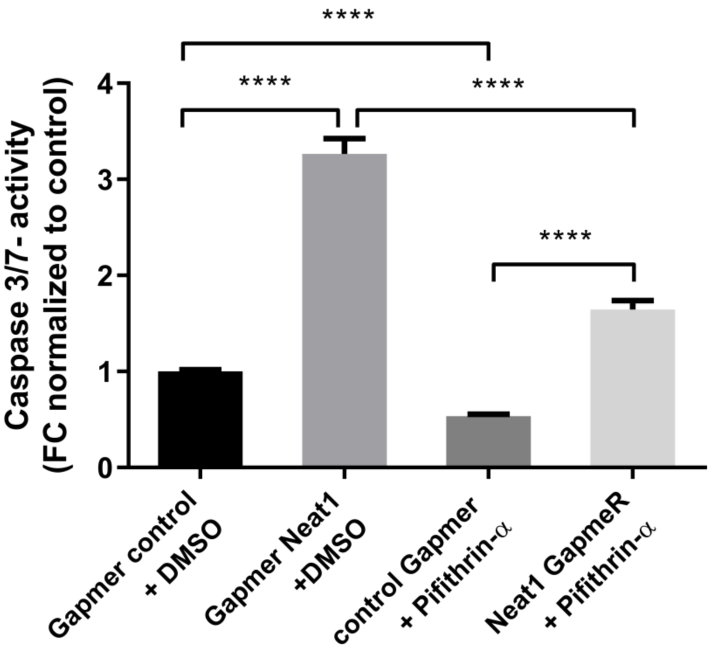

Figure 9: Apoptosis induction by *Neat1* loss is in part P53-independent

Caspase-3/7 activity was measured after treatment of 3T3 cells with GapmeR control and dimethyl sulphoxide (DMSO)/Pifithrin-α and GapmeR *Neat1* and DMSO/Pifithrin-α. Data are presented as mean ±SEM. N=3 independent experiments with 3 biological replicates. **** $p \leq 0.0001$; One-way ANOVA

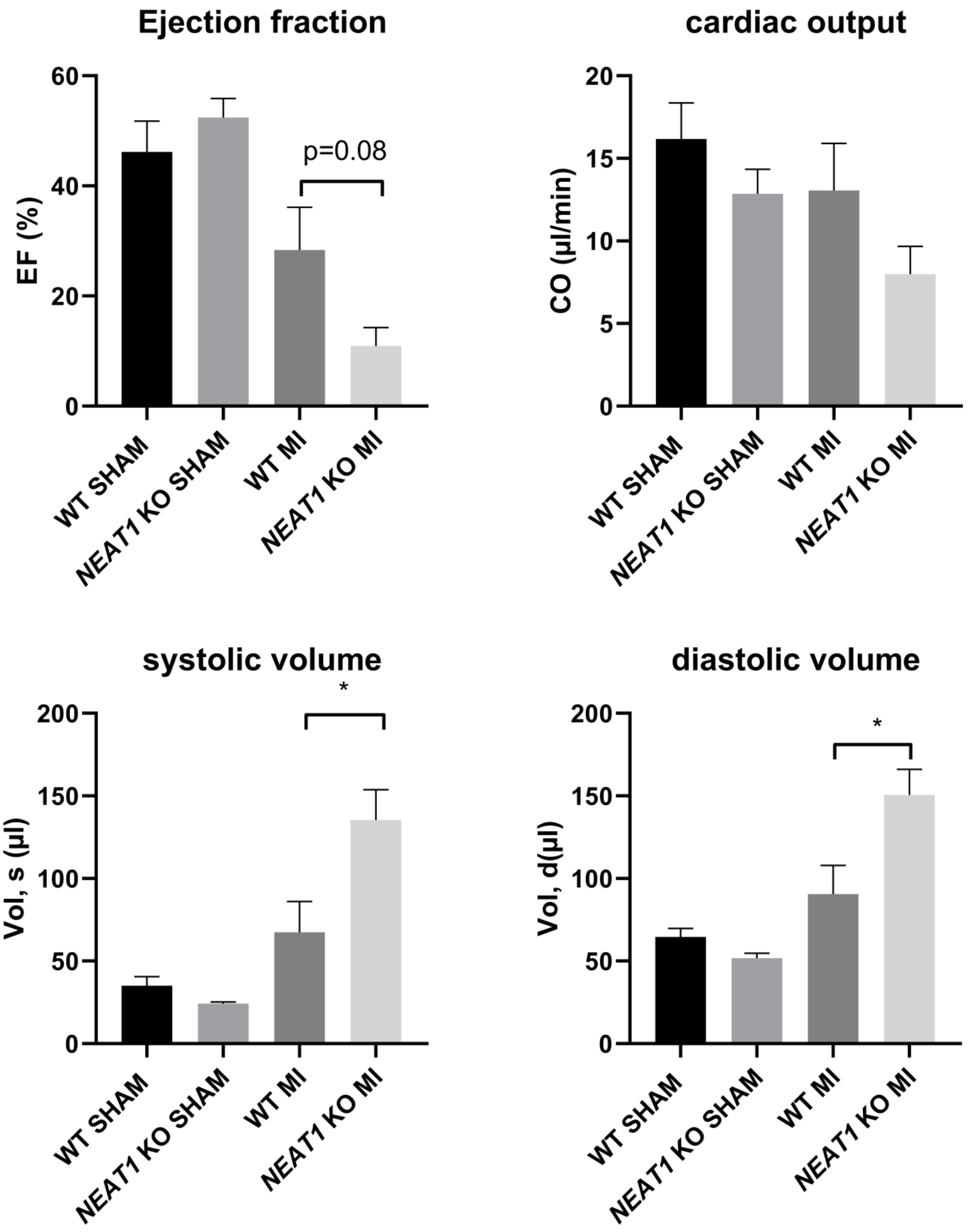

Figure 10: *Neat1* deficiency leads to impaired heart function after myocardial infarction.
Echocardiographic parameters of *Neat1* KO or wildtype (WT) mice were assessed 14 days after permanent LAD ligation or sham operation. EF=ejection fraction, CO=cardiac output. N=3-5 animals per group; Data are presented as mean ±SEM. * $p \leq 0.05$; Student´s t-test

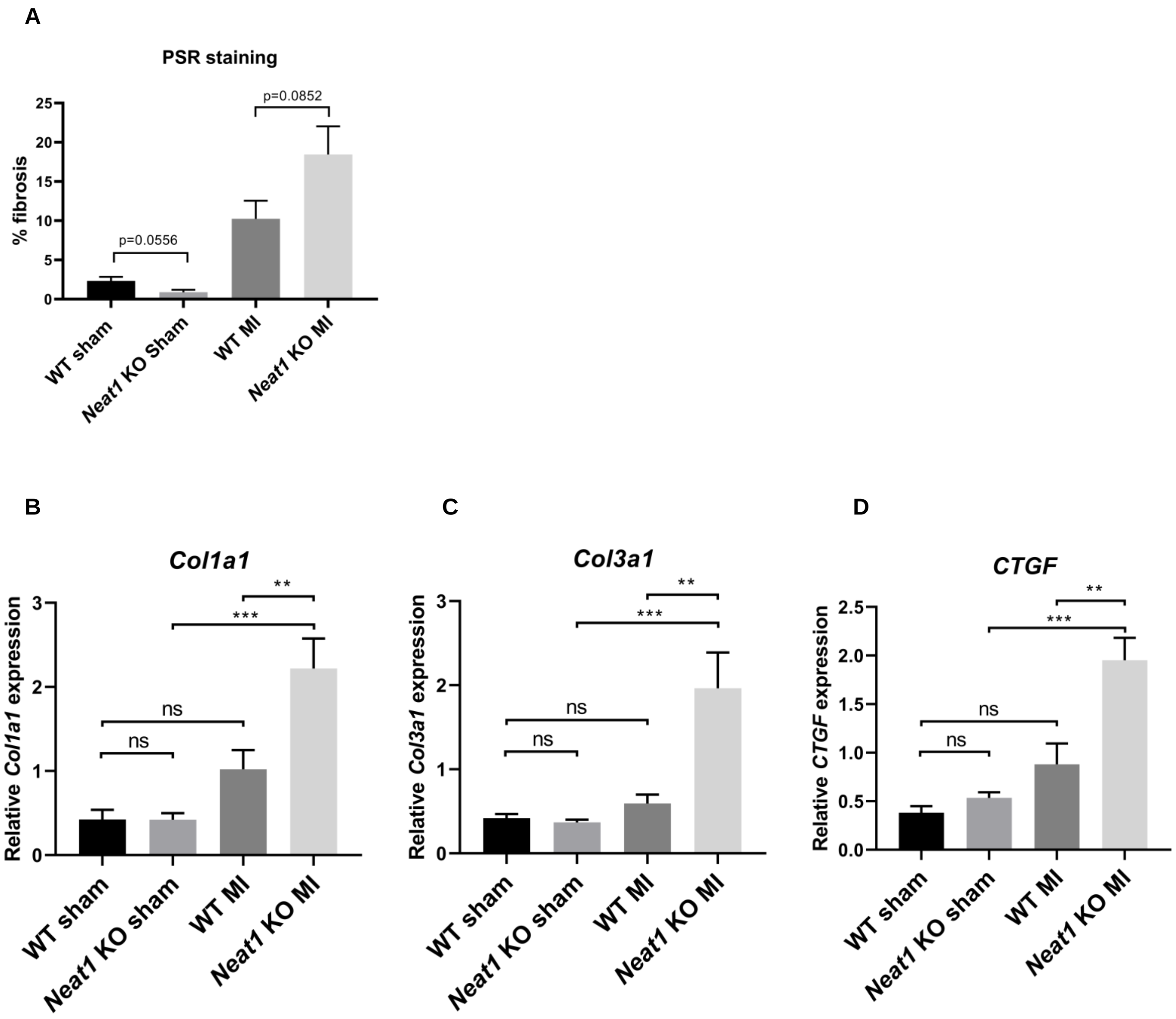

Figure 11: *Neat1* knockout elevates fibrosis.
(A) Picrosirius red staining (PSR) of left ventricles in *Neat1* knockout (KO) or wildtype (WT) mice 28 days after permanent left anterior descending artery (LAD) ligation (MI) or sham operation. **(B-D)** Murine hearts from *Neat1* KO or wildtype mice were removed 28 days LAD ligation and dissected into the remote and the peri-/infarct zone. Expression level of fibrotic markers *Col1a1*, *Col3a1* and *CTGF* was measured in the remote zone. of N=3-5 animals per group; Data are presented as mean ±SEM. ** $p \leq 0.01$;*** $p \leq 0.001$; ns=not significant. One-way ANOVA with Tukey´s multiple comparison test

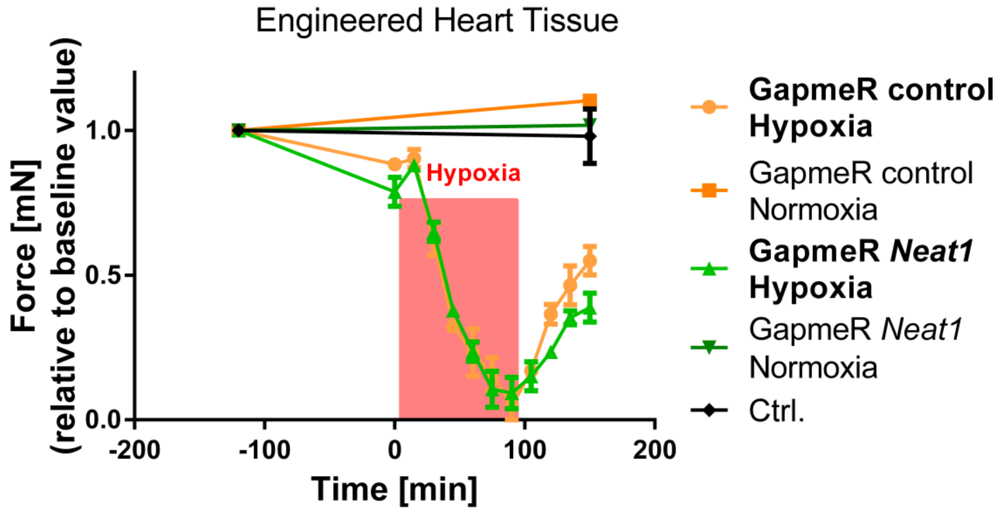

Figure 12: *Neat1* influences force recovery in an hypoxia/reoxygenation *ex vivo* model
Measurement of force in human engineered heart tissue under basal and hypoxic/reoxygenation conditions after treatment with GapmeR *Neat1* or GapmeR control. n=4

Supplements:

Methods:

Treatment of cells

To prevent HIF degradation, cells were treated with an inhibitor for the prolyl-hydroxylase which normally degrades the HIF protein. Therefore HL-1 cells were grown to a confluence of ~80 % and then incubated in medium supplemented with 1 mM DMOG (Dimethyloxalylglycine, Sigma) for 24h.

To deactivate P53 activity, cells were treated with Pifithrin-α (Enzo Life Sciences). Therefore cells were cultured in DMEM supplemented with 10 % FBS, 1 % P/S and 10 µM Pifithrin-α for 48h. As Pifithrin-α is dissolved in DMSO, the same volume of DMSO was used for the control group.

To induce P53 activity, cells were treated with 10 µM Nutlin-3 (Sigma Aldrich) for 48 h. As Nutlin-3 is dissolved in methanol, the same volume of methanol was used for the control group.

Preparation of EV production medium

To avoid the influence of exosomes present in the serum, FBS has to be pre-depleted of the contaminating exosomes. Therefore, FBS was ultracentrifuged over night for at least 10 h at 100,000xg and 4 °C.

Co-culture assay

For co-cultivation experiments, HL-1 cells were exposed to hypoxic (5 % CO2, 0.2 % O2 and 37 °C) or normoxic (5 % CO2, 21 % O2 and 37 °C) conditions for 24 h following 4 h reoxygenation and conditioned medium was collected. Next, 3T3 fibroblasts were incubated with HL-1 cardiomyocyte conditioned medium containing extracellular vesicles (in a ratio 1:3 fibroblast medium + HL-1 supernatant).

Scratch assay

NIH 3T3 cells were cultured in a 6-well plate and after 24 h transiently transfected with 50 nM GapmeR Neat1 or GapmeR control. On the next day, cells were trypsinized and 70 µl of cell suspension was transferred into each IBIDI insert (Thermo Fisher Scientific #80209). After additional 24 h, a horizontal scratch was performed, inserts were removed and images were immediately taken (=starting point t0). The migration of the cells was further tracked by taking images after 4-6 h. Afterwards, the area between the scratch was measured at each timepoint and the migration index was calculated according to: (area t0h-area t6h) / area t0.

Caspase3/7 assay

To assess the level of apoptosis, a Caspase 3/7 assay (Promega) was performed according to the manufacturer´s recommendations. Briefly, 48 h after the desired treatment, the same amount of Caspase-Glo 3/7 reagent as the culture medium was added to each well of a 96-well plate and carefully mixed by pipetting up and down several times. Afterwards, the plate was kept at room temperature in the dark for 1 h. Then, the supernatant (180 µl) was transferred to a white-walled 96-well plate and the developed luminescence was acquired in a luminometer (Synergy HT Reader, BioTek). Cells treated with 2 µM staurosporin for 3h served as a positive control.

Cell cycle assay

For analysis of the distribution of cells in cell cycle phases the Guava cell cycle flow cytometry based assay (Millipore) was used. Therefore, the cells were treated with the desired method, the medium was collected in a falcon and the cells harvested using the normal trypsinization protocol. Then, the cells were washed with PBS, transferred to the falcon containing the medium and centrifuged for 5 min at 300xg and 4 °C. After discarding the supernatant, the pellets were resuspended in 200 µl PBS and the suspension was transferred to a 96-well U-bottom plate before being centrifuged at 450xg for 5 min at RT with the brake on low. The supernatant was carefully removed and the remaining pellet was washed with 200 µl PBS and centrifuged as above. The supernatant was again carefully discarded leaving a small drop of PBS to resuspend the cells before adding dropwise 200 µl ice-cold 70 % ethanol to fixate the cells. Then, the plate was kept for at least 1 h at 4 °C. In order to stain the cells with propidium iodide (PI), which intercalates into nucleic acids and therefore gives a correlation with the DNA content of the cells, the cells were first again centrifuged at 450xg for 5 min at RT with the brake on low. After removing the supernatant the remaining pellet was resuspended in 200 µl PBS and incubated for 1 min at RT before being centrifuged as above. The supernatant was carefully discarded and 200 µl Guava Cell Reagent (containing PI to stain the cells) was added and mixed by pipetting up and down. The suspension was transferred to a new tube and incubated for 30 min at RT in the dark. Afterwards, the mixture was analyzed by flow cytometry measurements with Guava easyCyte (EMD Millipore).

RNA Isolation

The isolation of total RNA was performed using the TriFast (Peqlab) method according to manufacturer's instructions. Briefly, cells were washed with PBS and samples were homogenized directly from cell culture plates or flasks using 1 ml TriFast. After transferring to an Eppendorf tube, shaking vigorously and incubation for 5 min at RT for the complete dissociation of nucleoproteins, 200 µl chloroform was added to the samples. After inverting the tube and incubation for 3-10 min at RT, the different phases were separated by centrifugation at 12000 x g at RT for 5 min. 500 µl of the upper phase was transferred to a new tube and mixed with 500 µl Isopropanol and incubated for 10 min on ice to precipitate the RNA. A centrifugation step at 12000 x g for 10 min was followed, the supernatant was removed and the RNA pellet was washed twice with 1 mL of 75 % (v/v) ethanol by inverting the tube for 15 s. The RNA pellet was dried and dissolved in usually 20-30 µl RNase-free water and stored at -80 °C. The RNA-concentration was measured at 260 nm and 280 nm and the ratio was calculated using the Synergy HT multi-mode Reader (Biotek).

RNA Isolation of EVs

For the isolation of total RNA of EVs, supernatant, RNA microarray analysis and subcellular fractionation experiments the miRNeasy Mini Kit (Qiagen) was applied according to manufacturer's instructions. Briefly, samples were homogenized using 5 volumes of QIAzol Lysis reagent by vortexing. After an incubation time of 5 min, 5 µl of 1 fmol/µl spike-In-RNA (cel-miR-39 (Sigma-Aldrich)) was added followed by addition of 200 µl chloroform and 15 s of vortexing for immediate phase separation. The samples were incubated for 2–3 min and centrifuged for 15 min at 12,000xg at 4 °C. After transferring the upper aqueous phase (300 µl) into a new tube, 450 µl of 100 % ethanol was added and the samples were mixed by inverting the tubes. 700 µl of the sample were pipetted into an RNeasy Mini spin column in a 2 ml collection tube and centrifuged at 9600xg for 15 s at RT. Then the

flow-through was discarded and 700 µl Buffer RWT was added to the column, followed by 15 s of centrifugation at 9600xg at RT. Again the flow-through was discarded and the column was washed two times with 500 µl Buffer RPE and centrifuged at 9600xg for 15 s at RT. After placing the RNeasy Mini spin columns into a new 2 ml collection tube, the columns were again centrifuged at full speed for 2 min at RT to dry the spin column membrane and to ensure that no ethanol is carried over during RNA elution. The RNeasy Mini spin column was placed into a new 1.5 ml collection tube and 15 µl RNase-free water was added directly onto the RNeasy Mini spin column membrane and incubated for 5 min at RT and then centrifuged at 9600xg for 1 min at RT. To obtain a higher RNA concentration this step was repeated. The samples were stored at -80° C.

DNAse digestion

For detection of Neat1 or subcellular fractionation experiments total RNA was DNase treated with the on-column RNase-free DNase Set (Qiagen) prior to elution of RNA. After washing the column with RWT buffer, the flow-through was discarded and 10 µl DNase I stock solution (2.7 U/µl) was mixed with 70 µl RDD buffer and directly pipetted onto the column. After an incubation time of 15 min at RT 500 µl Buffer RWT (prepared with isopropanol) were added and the samples were centrifuged for 15 s at 9600xg. The flow-through was discarded and the protocol was continued according to section 4.2.2 with adding buffer RPE into the column.

Reverse transcription

For gene expression analysis, cDNA synthesis was performed using iScript select cDNA synthesis Kit (Bio-Rad) according to the manufacturer´s instructions. 5 µl of total RNA (100-1000 ng) was reverse transcribed using 2 µl Oligo (dT)20 or random primer, 4 µl iScript reaction mix and 1 µl iScript reverse transcriptase. The reaction mixture was filled up to 20 µl with dH20. For the cDNA synthesis using Oligo (dT)20 primers the protocol was the following: 42 °C for 90 min, followed by inactivation at 85 °C for 5 min. Reverse transcription using random primer reaction mix was performed at 25 °C for 5 min, 42 °C for 30 min and 85 °C for 5 min. The samples were stored at -20 °C.

Quantitative Real-Time PCR (qRT-PCR)

Quantification of the amount of specific gene expression was performed via real-time quantitative PCR (qPCR) using iQ SYBR Green Supermix (Bio-Rad). The qPCR was carried out in a 384-well plate using the ViiA™ 7 Real-Time PCR System (Thermo Fisher Scientific). The reaction mixture was the following: 5 µl iQ SYBR Green Supermix (Bio-Rad), 0.5 µl of a primer set (10 µM of each primer) or 1 µl of QuantiTect pimer assay (10x), 2 µl of cDNA (1:3 pre-diluted in nuclease-free water) and 2.5 µl or 2 µl nuclease-free water. The qPCR was run at 95 °C for 3 min to activate Taq polymerase, followed by 45 cycles of denaturation at 95 °C for 15 s, annealing at 60 °C or 58 °C for 30 s and elongation at 72° C for 40 s. After the amplification, a melting curve was generated by measuring fluorescence every 0.5 °C from 95 °C to 55 °C for 10 s to ensure a single PCR product. The qPCR data were analysed and gene expression was calculated using either the ΔΔct method or software-calculated starting quantities (SQ). These values were calculated based on the measurement of a standard curve generated from pooled cDNA samples (undiluted to 1:125 diluted). The relative gene expression was calculated by dividing each SQ mean value of a gene to the respective sample SQ mean value of a reference gene. As reference genes 18S ribosomal RNA (18S) or Gapdh were used.

Subcellular fractionation

In order to measure the subcellular distribution of lncRNAs and mRNAs in cytoplasmic, nuclear-soluble and chromatin-associated RNA fractions, fragmentation of HL-1 cardiomyocytes was performed as previously described 132. Therefore, 1 x 106 cells were harvested and centrifuged at 168xg at room temperature for 5 min. After discarding the supernatant, the resulting cell pellet was lysed in 175 µl cold sample buffer (50 mM Tris-HCl pH 8, 140 mM NaCl, 1.5 mM MgCl2, 0.5% Igepal NP-40, 2 mM Vanadyl Ribonucleoside complex) and incubated for 5 min. on ice. Then, the cell suspension was centrifuged at 300xg at 4 °C and the resulting supernatant (cytoplasmic fraction) was transferred into a new tube and stored on ice. The nuclear pellet was resuspended in 175 µl cold RLN2 solution (50 mM Tris-HCl pH 8, 500 mM NaCl, 1.5 mM MgCl2, 0.5% NP-40, 2 mM Vanadyl Ribonucleoside complex) and incubated for 5 min. on ice. After centrifugation for 2 min. at 16,360xg at 4 °C, the resulting pellet (chromatin-associated fraction) and supernatant (nuclear-soluble fraction) were stored on ice. Afterwards, 1 ml of Qiazol was added to all the fractions and RNA isolation was performed using the miRNeasy Mini Kit (Qiagen) including DNA digestion steps.

LncRNA profiling

For microarray analysis, total RNA was isolated from cardiomyocyte-derived microvesicles and exosomes as well as HL-1 cells using the miRNeasy Mini Kit (n=3) and the RNA quality was assessed using the Agilent Bioanalyzer 2100. These samples were globally profiled using the Arraystar Mouse LncRNA Microarray v 3.0 (Arraystar Inc, Rockville, MD) that allows a simultaneous detection of 35,923 lncRNAs and 24,881 coding transcripts. The microarray was performed according to the Agilent One-Color Microarray-Based Gene Expression Analysis protocol (Agilent Technology). Briefly, ribosomal RNA was separated from mRNA using the mRNA-ONLY™ Eukaryotic mRNA Isolation Kit (Epicentre) before amplifying and transcribing each sample into fluorescent cRNA along the entire length of the transcripts without 3' bias utilizing a random priming method (Arraystar Flash RNA Labeling Kit, Arraystar). Then, the labelled cRNAs were hybridized onto the Mouse LncRNA Array v3.0 (8 x 60K, Arraystar), the slides were washed and the arrays were scanned by the Agilent Scanner G2505C. Agilent Feature Extraction software (version 11.0.1.1) was used to analyze the acquired array images.

After quantile-normalization of the raw data with the GeneSpring GX v12.1 software package (Agilent Technologies), lncRNAs that at least have 6 out of 18 samples flags in Present or Marginal ("All Targets Value") were chosen for further data analysis. Dysregulated lncRNAs were identified through Volcano Plot filtering ($p \leq 0.05$, $FC \leq 0.5$ or ≥ 2).

RNA Seq

Library generation, quality control, and quantification:

500 ng of total RNA per sample were utilized as input for mRNA enrichment procedure with 'NEBNext® Poly(A) mRNA Magnetic Isolation Module' (E7490L; New England Biolabs) followed by stranded cDNA library generation using 'NEBNext® Ultra II Directional RNA Library Prep Kit for Illumina' (E7760L; New England Biolabs). All steps were performed as recommended in user manualE7760 (Version 1.0_02-2017; NEB) except that all reactions were downscaled to 2/3 of initial volumes. Furthermore, one additional purification step was introduced at the end of the standard

procedure, using 1x 'Agencourt® AMPure® XP Beads' (#A63881; Beckman Coulter, Inc.) and fragmentation time was set to 12 minutes.
cDNA libraries were barcoded by single indexing approach, using 'NEBNext Multiplex Oligos for Illumina – Set 1 and 2' (Index Primer 1-12, 18-23). All generated cDNA libraries were amplified with 6 cycles of final pcr.
Fragment length distribution of individual libraries was monitored using 'Bioanalyzer High Sensitivity DNA Assay' (5067-4626; Agilent Technologies). Quantification of libraries was performed by use of the 'Qubit® dsDNA HS Assay Kit' (Q32854; ThermoFisher Scientific).

Library denaturation and Sequencing run:

Equal molar amounts of eighteen individually barcoded libraries were pooled. Accordingly, each analyzed library constitutes 5.6% of overall flowcell capacity. The library pool was denatured with NaOH and was finally diluted to 1.5pM according to the Denature and Dilute Libraries Guide (Document # 15048776 v02; Illumina). 1.3 ml of denatured pool was loaded on an Illumina NextSeq 550 sequencer using a High Output Flowcell for paired-end reads (20024907; Illumina). Sequencing was performed with the following settings: Sequence reads 1 and 2: 81bp each; Index read 1: 6bp.

BCL to FASTQ conversion:

BCL files were converted to FASTQ files using bcl2fastq Conversion Software version v2.20.0.422 (Illumina).

Raw data processing and quality control:

Raw data processing was conducted by use of nfcore/rnaseq (version 1.5dev) which is a bioinformatics best-practice analysis pipeline used for RNA sequencing data at the National Genomics Infrastructure at SciLifeLab Stockholm, Sweden. The pipeline uses Nextflow, a bioinformatics workflow tool. It pre-processes raw data from FastQ inputs, aligns the reads and performs extensive quality-control on the results. The genome reference and annotation data were taken from GENCODE.org (Mus musculus; GRCm38.p6; release M17).

Normalization and differential expression analysis:

Normalization and differential expression analysis was performed with DESeq2 (Galaxy Tool Version 2.11.40.2) with default settings except for "Output normalized counts table" which was set to "Yes".

Gene enrichment analysis

Ensembl gene ID list of the 745 significant genes (padj<0.05) were submitted to DAVID functional annotation tool (1) for gene set enrichment analysis. GOTERM biological process were used as annotation resource.

RNA FISH

In order to detect and localize lncRNAs within the cell, RNA fluorescent in situ hybridization was performed. Therefore, cells were seeded on 18 mm round coverglass slips in a 12-well plate and cultured for 24 h before be exposed to normoxic (21 % O2) or hypoxic (0.1 % O2) condtions for additional 24 h. For fixation, the cells were washed with PBS and 1 ml fixation buffer (3.7 % (v/v) formaldehyde in 1xPBS) was added. After incubation for 10 min at RT, the cells were washed twice with 1xPBS and permeabilized in 1 ml 70 % ethanol for 1 h at 4 °C. The ethanol was removed and 1 ml wash buffer A (10% formamide in Stellaris RNA FISH Wash Buffer A (Biosearch Technologies)) was added. After incubation for 5 min at RT, a 100 µl drop of hybridization buffer (10 % formamide in Stellaris RNA FISH Hybridization Buffer (Biosearch Technologies)) containing the probe (125 nM) was added onto a parafilm. The coverslip was transferred to the drop with cells facing down an incubated in the dark for 16 h at 37 °C in a humidified chamber. On the next day, the coverglass slips were transferred to a new 12-well plate containing 1 ml wash buffer A and incubated in the dark at 37 °C for 30 min. For staining the nuclei, the wash buffer was aspirated and 1 ml DAPI solution (wash Buffer A consisting of 5 ng/ml DAPI) was pipetted in each well and incubated for additional 30 min in the dark at 37 °C. After removing the staining solution, 1 ml wash buffer B (Biosearch Technologies) was added and incubated for 5 min at RT. Afterwards, the coverslips were mounted in Vectashield mounting medium and images were taken.

Electron microscopy

Purified cardiomyocyte-derived vesicles were used for electron microscopic analysis as described previously 61. In brief, EVs were isolated using the above mentioned protocol and resuspended in 50 µl fixation solution (0.1 % glutaraldehyde, 4 % PFA in 0.2 mol HEPES buffer). Then, the fixed EVs were loaded onto formvar carbon-coated grids and dried for 20 min at 40 °C. The loaded grids were again fixed in 1 % glutaraldehyde (Agar Scientific Limited) in HEPES buffer for 5 min and subsequently transferred to a drop of 100 µl distilled water several times. After contrasting the grids in 4 % uranyl-acetate solution for 5 min at RT, the grids were finally embedded in a mixture of uranyl acetate (4 %) and methyl cellulose (2 %) on ice for 5 min. For removing excess fluid, the grids were placed on filter paper and dried for 10 min before they were stored in a grid storage box. Ultra-thin sections of cardiomyocyte-derived vesicles were analyzed with an electron microscope (Morgagni 268, FEI) and micrographs of representative areas were taken by a digital camera (Veleta TEM camera, Olympus Europa Holding GMBH).

Fluorescence confocal microscopy

To investigate a potential vesicle uptake into fibroblasts, immunofluorescence confocal microscopy measurements were performed. Therefore, cover slips were placed into wells of a 24-well plate and fibroblasts were seeded accordingly. On the next day, labelled vesicles were incubated with fibroblasts for 30 min, 2 h and 20 h at 37 °C or 20 h at 4 °C. After washing the cells carefully with PBS, the cells were fixed in 4% paraformaldehyde (PFA) for 15 min at RT. Then, the cells were washed again twice with PBS and permeabilized with 0.1 % Triton X-100 for 10 min at RT following two washing steps. In the next step, the cells were stained with Dapi (1:1000, Sigma) and TRITC-Phalloidin (1:100, Sigma) in 5 % Donkey serum in PBS for 30 min at RT in the dark. After washing with PBS, slides

were transferred to a microscope slide and embedded with Prolong Gold Antifade reagent (life technologies). Confocal imaging was performed with a Zeiss LSM 780 microscope using a Plan-Apochromat 63x/1.40 Oil immersion objective. In all cases z-stacks were taken covering the entire cell volume.

Cardiac cell fractionation

To assess the cardiac cell-specific gene expression of Neat1, cardiac cells were isolated from C57BL/6N mice. Therefore, mice were anaesthetized via inhalation of 3 % isoflurane in a chamber and injected with 100 µl heparin (500 I.E./ml, i.p.). After cutting the skin between two tracheal trabeculae, the thorax was opened and the heart was cannulated through the aorta. The heart was then excised and immediately retrograde perfused with pre-warmed perfusion buffer for 6 min (113 mM NaCl, 4.7 mM KCl, 0.6 mM KH2PO4, 0.6 mM Na2HPO4, 1.2 mM MgSO4-7H2O, 0.032 mM Phenol Red, 12 mM NaHCO3, 10 mM KHCO3, 10 mM HEPES, 30 mM Taurine, 0.1 % Glucose, 10 mM 2,3-Butanedione monoxime) followed by pre-warmed digestion buffer (113 mM NaCl, 4,7 mM KCl, 0.6 mM KH2PO4, 0.6 mM Na2HPO4, 1.2 mM MgSO4-7H2O, 0.032 mM Phenol Red, 12 mM NaHCO3, 10 mM KHCO3, 10 mM HEPES, 30 mM Taurine, 0.1 % Glucose, 10 mM 2,3-Butanedione monoxime, 12.5 µM CaCl2, 700 U/ml Collagenase II) for 20 minutes. After removing the atria, cardiac ventricles were further dissociated mechanically by cutting into small pieces using digestion buffer and shearing through a 1 ml syringe. Collagenase II digestion was stopped by adding stop buffer (perfusion buffer supplemented with 10 % FBS and 12.5 µM CaCl2). The obtained cell suspension was filtered through a 100 µm cell strainer and mixed with AMCF medium containing 1 % FBS (10.8 g MEM, 10 % NaHCO3, 2ng/ml vitamin B12, 100 U/ml penicillin/100 µg streptomycin). To separate cardiomyocytes and non-myocytes, the cell suspension was sedimented for 10 min on ice. CMC pellets were washed in PBS, centrifuged for 5 min at 3000 rpm at 4 °C, frozen in liquid nitrogen and stored at -80 °C. The remaining supernatants containing the non-myocyte fraction were centrifuged at 1500 rpm for 5 min at RT and the resulting cell pellets dissolved in plating medium containing 1 % FBS and pre-plated for 1 h on a 10 cm petri dish in a 1 % CO2 incubator. The attached cells were washed with PBS and harvested with a cell scraper. After centrifugation at 3000 rpm for 5 min at 4 °C, the pellets were frozen in liquid nitrogen and stored at -80 °C. The remaining pre-plating medium, containing non-attached endothelial cells, was then centrifuged at 1500 rpm for 5 min at 4 °C. Afterwards, the resulting pellets were resuspended in MACS buffer (MACS bovine serum albumin stock solution diluted 1:20 in auto-MACS rinsing solution, both from Miltenyi Biotec) and mixed with CD146 MACS beads (Miltenyi Biotec) and incubated for 15 min at 4 °C. Then, additionally MACS buffer was added. After a centrifugation step at 1500 rpm for 5 min at 4 °C, the resulting pellets were suspended in MACS buffer and applied on a MACS separating column. The columns were washed 3 times with MACS buffer and then removed from the magnetic field. The resulting ECs were collected in MACS buffer and centrifuged at 3000 rpm for 5 min at 4 °C. The resulting pellets were frozen in liquid nitrogen and stored at -80 °C.

In vivo vesicle isolation

Cardiac EVs were isolated from the left ventricule of C57Bl6 mice with sham or infarcted hearts by our cooperation partners in Paris (Silvestre, Loyer, Boulanger, Cardiovascular research centre, INSERM) as published recently (2). Those vesicles were isolated between 0 and 14 days after

coronary artery ligature according to the method previously established for ischemic skeletal muscle 133. Briefly, murine hearts were perfused with saline solution, excised and rinsed. The left ventricle was isolated and minced mechanically for 30 s. Homogenates were centrifuged to remove tissue, before pelleting MVs and Exo via sequential centrifugation and filtration steps. The different EV subtypes were further characterized using NTA and the cellular origin of EVs was determined by high sensitive flow cytometry.

References

1. Huang DW, Sherman BT, Lempicki RA. Systematic and integrative analysis of large gene lists using DAVID bioinformatics resources. Nature protocols 2009;4(1):44–57.

2. Loyer X, Zlatanova I, Devue C, et al. Intra-Cardiac Release of Extracellular Vesicles Shapes Inflammation Following Myocardial Infarction. Circulation research 2018;123(1):100–6.
